# Digging deeper into the immunopeptidome: characterization of post-translationally modified peptides presented by MHC I

**DOI:** 10.1007/s42485-021-00066-x

**Published:** 2021-06-04

**Authors:** Kiran K. Mangalaparthi, Anil K. Madugundu, Zachary C. Ryan, Kishore Garapati, Jane A. Peterson, Gourav Dey, Amol Prakash, Akhilesh Pandey

**Affiliations:** 1 Department of Laboratory Medicine and Pathology, Mayo Clinic, 200 First ST SW, Rochester, MN 55905 USA; 2 Institute of Bioinformatics, International Technology Park, Bangalore, 560066 India; 3 Manipal Academy of Higher Education (MAHE), Manipal, 576104 Karnataka India; 4 Center for Molecular Medicine, National Institute of Mental Health and Neurosciences (NIMHANS), Hosur Road, Bangalore, 560 029 India; 5 Proteomics Core, Medical Genome Facility, Mayo Clinic, Rochester, MN 55905 USA; 6 Optys Tech Corporation, Shrewsbury, MA USA; 7 Center for Individualized Medicine, Mayo Clinic, Rochester, MN 55905 USA

**Keywords:** Immunopeptidome, MHC class I, MHC binding motifs, Post-translational modifications, Spliced peptides

## Abstract

Peptides presented by MHC molecules on the cell surface, or the immunopeptidome, play an important role in the adaptive arm of the immune response. Antigen processing for MHC class I molecules is a ubiquitous pathway present in all nucleated cells which generates and presents peptides of both self and non-self-origin. Peptides with post-translational modifications represent one category of peptides presented by MHC class I molecules. However, owing to the complexity of self-peptides presented by cells, the diversity of peptides with post-translational modifications is not well-studied. In this study, we carried out MHC Class I immunopeptidomics analysis of Loucy T-cell leukemia and A375 malignant melanoma cell line to characterize the diversity of post-translational modifications of MHC class I-bound peptides. Using high resolution mass spectrometry, we identified 25,761 MHC-bound peptides across both cell lines using Bolt and Sequest search engines. The enrichment method was highly specific as ~ 90% of the peptides were of typical length (8–12 amino acids long) and the motifs were expected based on previously reported motifs for MHC I alleles. Among the MHC-bound peptides, we identified phosphorylation as a major post-translational modification followed by deamidation. We observed site-specific localization of these post-translational modifications, at position P4 for phosphorylated peptides and position P3 for deamidated peptides. We identified a smaller number of peptides with acetylated and methylated lysine, possibly due to very low stoichiometric levels of these PTMs compared to phosphorylation and deamidation. Using PEAKS de novo sequencing algorithm, we identified spliced peptides that accounted for ~ 5–7% of MHC-bound peptides that were otherwise similar in their features as normal MHC-bound peptides. We validated the identity of several post-translationally modified peptides and spliced peptides through mass spectrometric analysis of synthetic peptides. Our study confirms post-translationally modified peptides to be present at low stoichiometric levels along with unusual spliced peptides through unbiased identification using high resolution mass spectrometry.

**Supplementary Information:**

The online version contains supplementary material available at 10.1007/s42485-021-00066-x.

## Introduction

The immunopeptidome presented by MHC class I molecules on the cell surface represents a unique set of intracellularly processed peptides in all nucleated cells. Circulating immune cells monitoring these MHC-bound peptides for the presence of foreign antigens form a crucial part of adaptive immunity. In the MHC class I antigen presentation pathway, endogenous proteins are degraded by proteasome machinery in the cytoplasm and products are transported to the endoplasmic reticulum before being further processed by endoplasmic reticulum resident proteases to a desired length. The peptides then associate with MHC class I molecules and are transported to the surface of the cell (Neefjes et al. 2011).

Because of the polymorphic nature of genes encoding the MHC molecules, notably class I, thousands of MHC class I alleles are known to exist (Robinson et al. 2017). This leads to an extraordinary diversity in peptides presented by these MHC alleles. Additional diversity is introduced through post translational modifications, defective ribosomal products (Yewdell et al. 2001), proteasome catalyzed spliced peptides (Vigneron et al. 2017) and peptides encoded by genomic variants. Peptides derived from genomic variation can include those that are specific to sequence alterations in tumor cells, commonly referred to tumor neoantigens, as they are not observed in the immunopeptidome of normal cells. Mass spectrometry has been the major analytical workflow used for the unbiased identification of thousands of peptides presented by MHC molecules (Bassani-Sternberg et al. 2016). Hitherto, the tumor neoantigens have largely been identified using next generations sequencing approaches followed by bioinformatics predictions and immunogenicity analysis (Linnemann et al. 2015; Tran et al. 2015). Large-scale identification of these tumor specific antigens using mass spectrometry has remained challenging thus far due to low expression of the variant proteins. Unlike tumor specific antigens, tumor-associated antigens represent proteins enriched in tumors, such as differentiation antigens and overexpressed proteins including proteins with post-translational modifications (Buonaguro et al. 2011). Several post-translational modifications identified among the MHC-bound peptides include phosphorylation, deamidation, citrullination, etc. (Engelhard et al. 2006). However, identification of these modified peptides has been challenging due to low stoichiometric levels of these PTMs. Another limiting factor has been the requirements for compute-intensive infrastructure for database searching with non-enzymatic cleavage sites along with additional post-translational modifications. For these reasons, published reports of unbiased searches for the post-translational modifications among immunopeptidome data sets are limited. In contrast to peptides with post-translational modifications, spliced peptides constitute a unique class of peptides generated post-translationally by the proteasome complex in the cytoplasm (Vigneron et al. 2017). Several recent studies have focused on identification and characterization of these spliced peptides (Liepe et al. 2016; Faridi et al. 2018), which are clinically relevant because of tumor-infiltrating CD8^+^ T lymphocyte responses (Mishto and Liepe 2017).

Here, we employ high resolution mass spectrometry to study the diversity of the MHC class I immunopeptidome from Loucy T-cell leukemia and A375 cell lines to identify several post-translationally modified peptides including spliced peptides.

## Methodology

### Culturing of cell lines

Loucy and A375 cell lines were obtained from ATCC. A375 cell line was cultured in DMEM medium and Loucy cell line was cultured in RPMI-1640 medium supplemented with 10% fetal bovine serum and 1% penicillin/streptomycin. The cell lines were maintained in CO_2_ incubator with 5% CO_2_ level. Upon reaching confluence, cell lines were washed with phosphate buffered saline (PBS) three times, flash frozen in liquid nitrogen and stored at – 80 °C.

### Crosslinking of W6/32 clone MHC class I antibody to protein A-sepharose 4B beads

Pan-specific MHC Class I antibody (W6/32 clone) was ordered from BioXCell. 5 mg of antibody was loaded on 1 mL of Protein A-Sepharose 4B beads (Invitrogen) packed in a polypropylene column (BioRad) and incubated for 30 min. Antibody-bound beads was washed with borate buffer (pH 9) and incubated with 20 mM dimethyl pimelimidate (DMP) linker for 45 min. Crosslinking reaction was stopped by incubating the column with ethanolamine for 2 h. Then the column was washed with phosphate buffer saline and stored in PBS with 0.02% sodium azide at 4 °C. On the day of experiment, column was washed with 0.1 N acetic acid and equilibrated with 100 mM Tris–HCl, pH 8.0.

### Enrichment of MHC–peptide complexes

MHC–peptide complexes were enriched using previously described protocol (Bassani-Sternberg et al. 2016). Loucy and A375 cell lines were lysed in buffer containing 0.25% sodium deoxycholate, 0.2 mM IAA, 1 mM EDTA, 1 mM PMSF, 1% Octyl-B-glucopyranoside, 1:200 protease inhibitor cocktail for 1 h on ice. Cell debris was separated by centrifugation of the lysate at 25,000×*g*, 4 °C for 50 min. The supernatant was loaded onto an MHC class I crosslinked affinity column and incubated for 1 h with gentle rotation. The column was washed with 150 mM NaCl in 20 mM Tris HCl pH 8.0, 400 mM NaCl in 20 mM Tris HCl pH 8.0 followed by 150 mM NaCl in 20 mM Tris HCl pH 8.0 and 20 mM Tris HCl pH 8.0. MHC-bound peptide complexes were eluted using 1% TFA and the eluate was subjected to C_18_ cleanup to purify the eluted peptides. The peptides were dried using a speedvac concentrator prior to LC–MS/MS analysis.

### LC–MS/MS analysis

LC–MS/MS analysis was carried out on an Orbitrap Eclipse Tribrid mass spectrometer (Thermo Scientific, San Jose, CA) connected online to a Dionex RSLC3000 liquid chromatography system (Thermo Scientific, San Jose, CA). Peptides were suspended in solvent A (0.1% formic acid) and loaded on a trap column (PepMap C_18_ 2 cm × 100 µm, 100 Å) followed by high resolution separation column (EasySpray 50 cm × 75 µm, C_18_ 1.9 µm, 100 Å, Thermo Scientific, San Jose, CA). The mass spectrometer was operated in data dependent mode with a cycle time of 2 s. Survey MS scan was acquired in Orbitrap mass analyzer with 120 K resolution, 4×e5 AGC target and 50 ms injection time. Monoisotopic precursor ions with charge state 2–4 were subjected to MS/MS with top scan priority followed by precursor with charge 1. Precursor ions (*z* = 2–4) were fragmented with 28% HCD normalized collision energy and acquired in orbitrap mass analyzer with 15 K resolution. Precursor ions (*z* = 1) with a mass range of 700–1400 *m/z* were fragment with 32% HCD NCE and analyzed in orbitrap analyzer. Dynamic exclusion was enabled with 30 s exclusion duration. Additional filters included monoisotopic precursor selection and intensity threshold of 2.5 × 10^4^.

### Database searching

Raw files were processed using Sequest in Proteome Discoverer 2.4 software and Bolt in Pinnacle software (v99.0) platforms. Database searching was performed using UniProt human canonical protein sequences with no enzyme specificity. Peptide length of 7–25 amino acids was considered for database searching with a precursor ion tolerance of 10 ppm, and fragment ion tolerance of 0.05 Da. Oxidation (methionine), phosphorylation (serine, threonine and tyrosine), deamidation (asparagine) and acetylation (protein N-terminus) were set as dynamic modifications in Sequest search engine. In addition to these modifications, two other modifications—acetylation (lysine) and methylation (lysine)—were included in the search using Bolt search algorithm. False discovery rate was maintained at 1% peptide level for both search engines.

### De novo sequencing for the identification of spliced peptides

De novo sequencing was performed using PEAKS Studio 10 software using search settings as described above. De novo search results (up to 5 sequence candidates for each spectrum) were filtered for an average local confidence (ALC) score for ≥ 80%. The resulting scans were further filtered for the scans without a peptide spectral match in the traditional database search. Scans were then filtered out if any of the de novo candidate sequences matched to the canonical human protein sequences. Only scans which passed all the above-mentioned criteria were considered for the identification of spliced sequences. De novo candidate sequences from these scans were mapped against all human protein sequences by partial sequence match. Candidate peptide sequences that completely map to a protein by splicing (cis-splicing) were identified using an in-house Python script. Finally, only the peptides which contained at least two amino acids in both spliced fragments were considered as confident spliced peptides.

### Data analysis

Unsupervised clustering of the peptides was performed using GibbsCluster server 2.0 using default parameters (Andreatta et al. 2017). The number of clusters with highest Kullbach Leibler distance was considered from the clustering results. Binding prediction of the MHC-bound peptides was carried out using NetMHC pan 4.1 server (Reynisson et al. 2020). Hydrophobicity indices of the spliced peptides were predicted using SSRCalc Version Q.O tool (Krokhin and Spicer 2009).

## Results and discussion

### Summary of the immunopeptidomics analysis

**Fig. 1:**
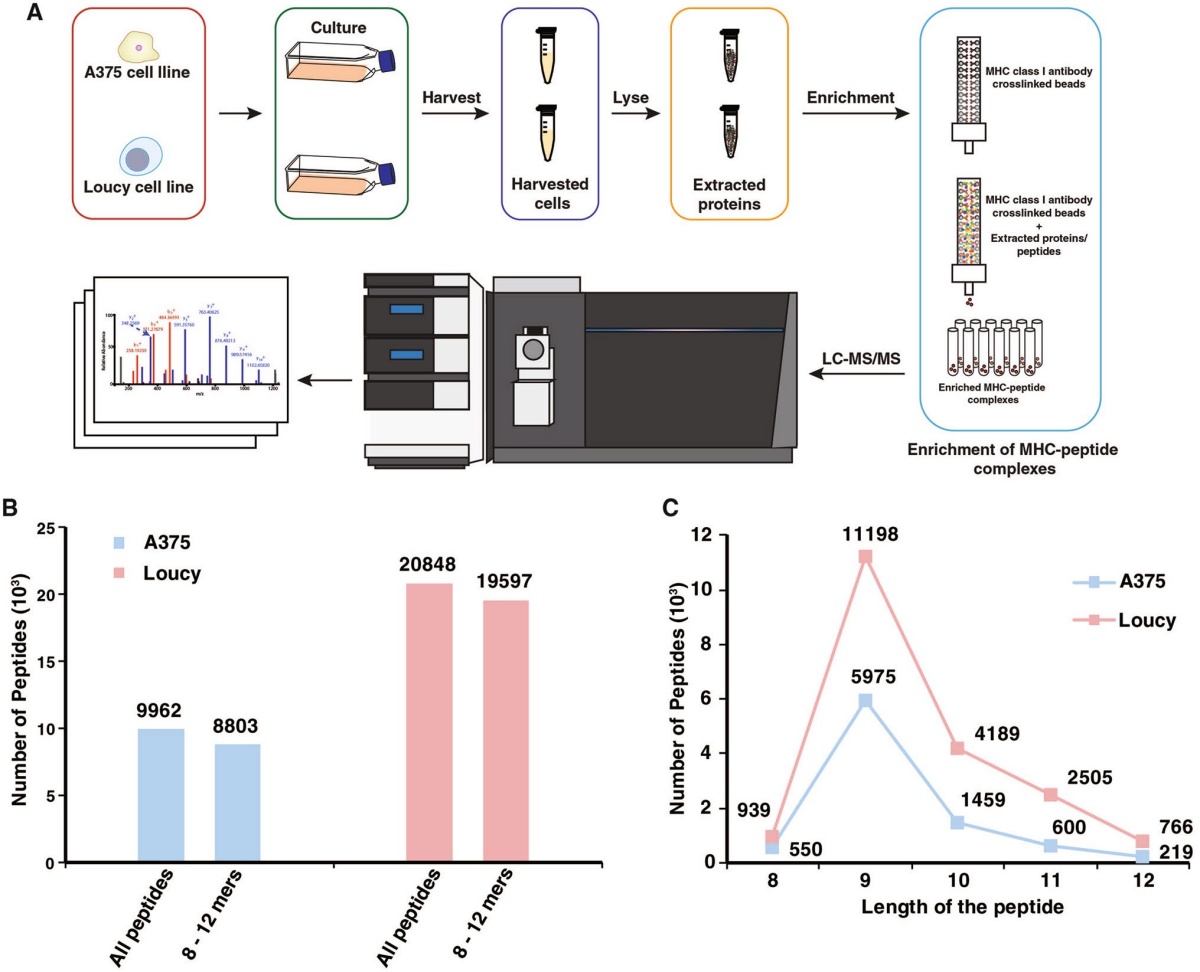
Summary of the immunopeptidomics analysis of Loucy and A375 cell lines. **A** Sample preparation workflow involved in the purification of MHC–peptide complexes followed by LC–MS/MS analysis. **B** Non-redundant number of peptides identified by LC–MS/MS analysis in A375 and Loucy cell lines using both Sequest and Bolt search engines. **C** Distribution of the number of peptides identified across 8–12 amino acids range in Loucy and A375 cell lines

In this study, immunoprecipitation of MHC complexes was carried out on Loucy T-cell leukemia cell line (1 × 10^9^ cells) and A375 malignant melanoma cell line (4 × 10^8^ cells) using pan-MHC-specific class I antibody (W6/32 clone). The workflow employed for the immunopeptidomics analysis is outlined in Fig. 1A. Cell pellets were lysed in a detergent lysis buffer followed by the immunoprecipitation and elution of MHC–peptide complexes. MHC-bound peptides were further purified from MHC molecules and β-2-microglobulin and subjected to LC–MS/MS analysis in technical replicates.

**Fig. 2:**
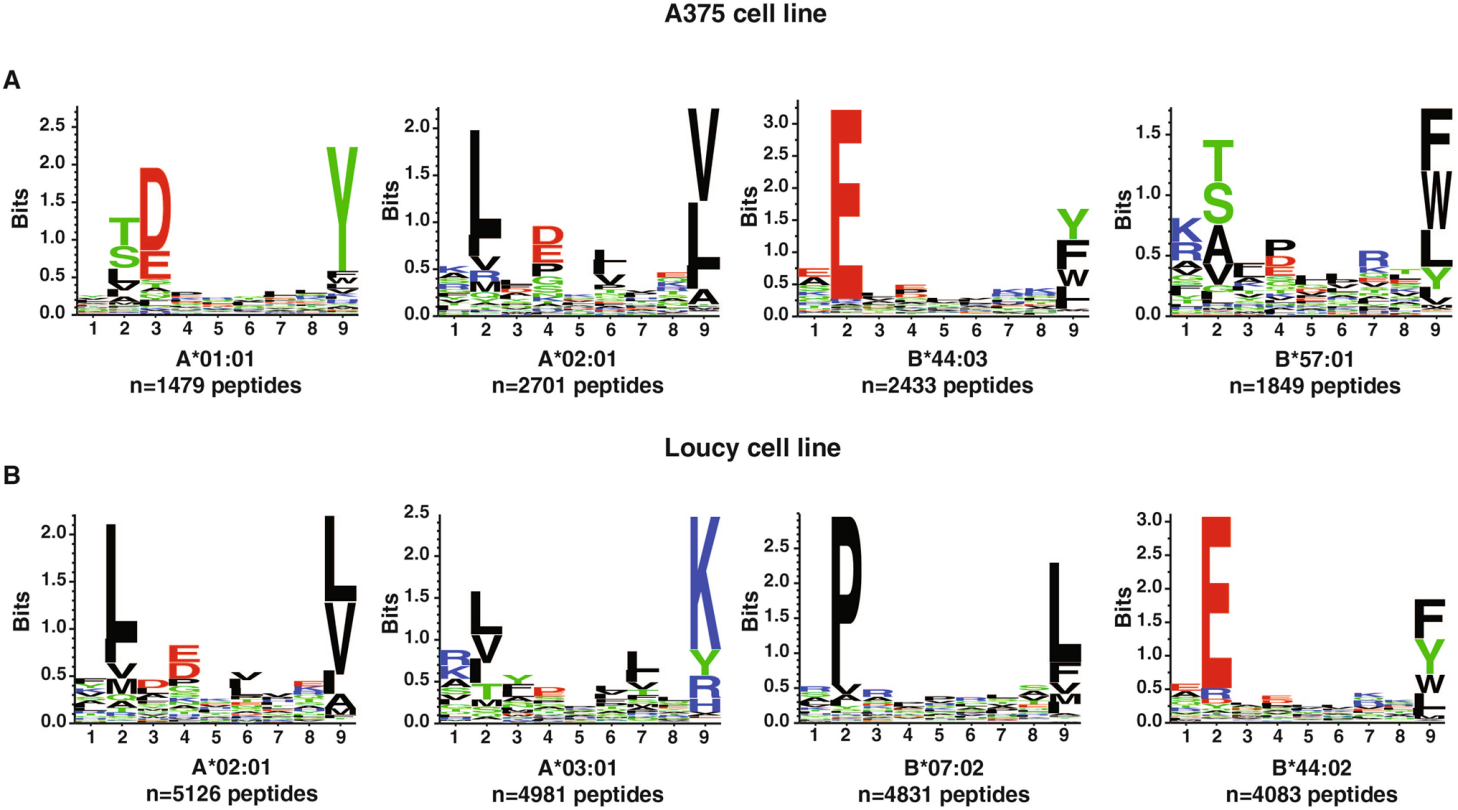
Unsupervised clustering of the identified peptides using GibbsCluster sever 2.0. Binding motifs enriched among the identified peptides with 8–12 aa length in A375 and Loucy cell lines

LC–MS/MS analysis was carried out using Orbitrap Eclipse mass spectrometer and raw files were processed using two different search engines, Sequest and Bolt. In all, 25,761 peptides were identified across both cell lines (19,597 in Loucy and 8803 in A375) with a limited overlap of ~ 10% peptide identifications across cell lines (Fig. 1B) (Supplementary Tables 1–4). Database searching using Bolt led to an increase in the number of peptides identified in both Loucy and A375 cell lines with ~ 65% of the peptides identified being common to both search engines (Supplementary Fig. 1). Peptides of conventional length for MHC class I-bound peptides (8–12 amino acids) represented ~ 90% of peptides identified from both cell lines (Fig. 1B). 9-mer peptides constituted the majority of the immunopeptidome across both the cell lines followed by 10-mer peptides (Fig. 1C). Motif enrichment analysis was performed using unsupervised clustering of peptides to identify the binding motifs among these peptides. Motifs identified from the clustering process corresponded to the binding motifs expected for the MHC alleles present in these cell lines (Fig. 2). In addition, binding affinity predictions of identified peptides with their respective MHC alleles showed that ~ 90% of the peptides were expected to be strong binders to the known MHC haplotype of Loucy and A375 cell lines. These results indicate that enrichment of MHC–peptide complexes followed by high resolution mass spectrometry reveals high specific and in-depth analysis of the MHC class I immunopeptidome.

### Identification of post-translationally modified peptides in immunopeptidome

Because of the large search space and prolonged database search time, only phosphorylation, deamidation and protein N-terminus acetylation were included as dynamic modifications during the Sequest search as these are the most abundant post-translational modifications in cellular systems. However, due to unique architecture of Bolt software, which makes it suitable in terms of speed and capacity to handle large databases, additional post-translational modifications—acetylation (K) and methylation (K)—were included in Bolt search.

**Fig. 3:**
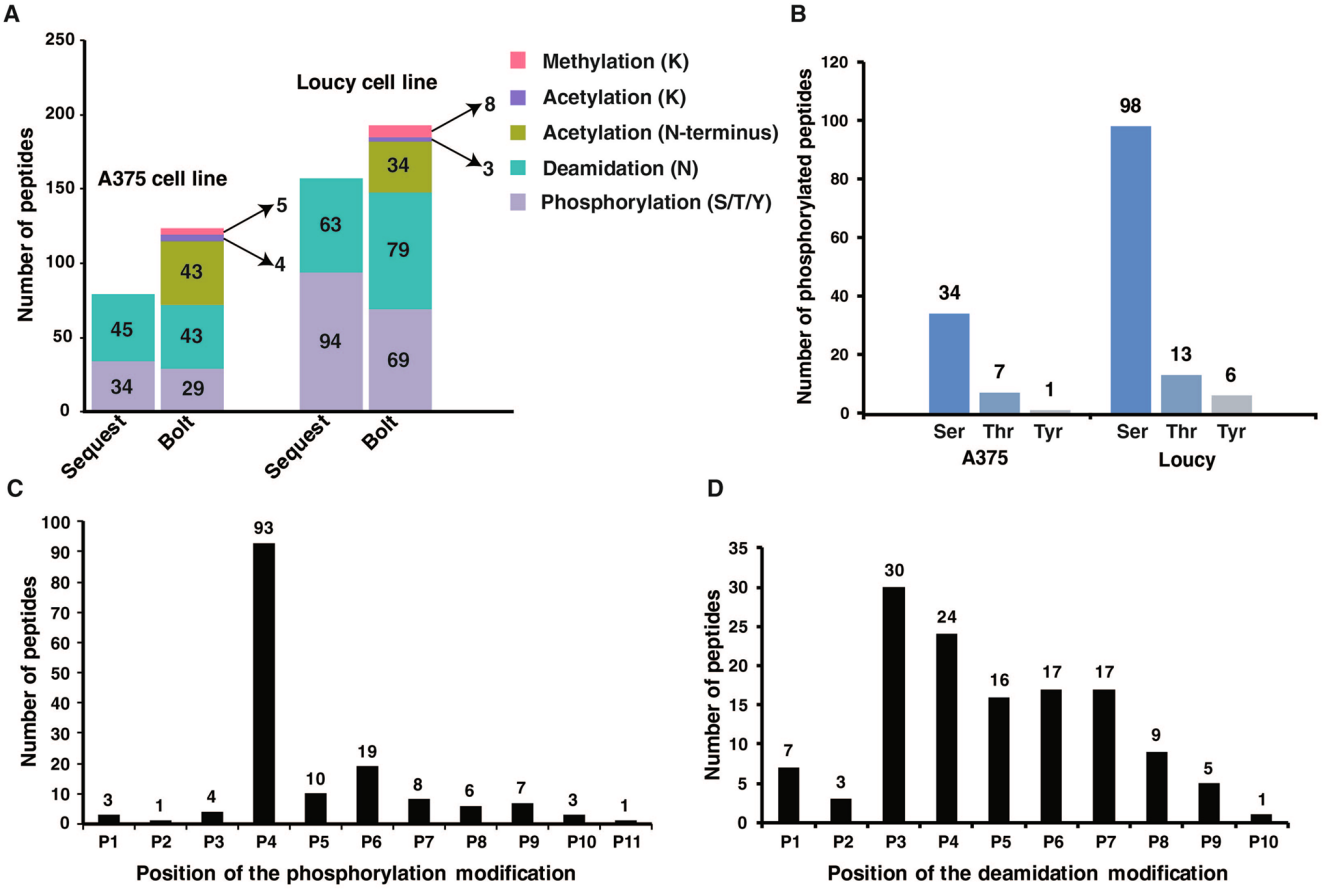
Identification of the post-translationally modified peptides. **A** Bar chart showing the number of post-translationally modified peptides by both Sequest and Bolt search engines in A375 and Loucy cell lines. **B** Number of peptides phosphorylated at serine, threonine and tyrosine residues in A375 and Loucy cell lines. **C**, **D** Localization of phosphorylation and deamidation modifications along the length of the peptides

Overall, we identified 277 and 153 post-translationally modified peptides (8–12 aa) in Loucy and A375, respectively (Fig. 3A). 42 and 117 phosphorylated peptides were identified in A375 and Loucy cell lines using both Sequest and Bolt search engines. Out of these peptides, 21 and 46 phosphorylated peptides were identified both by Sequest and Bolt. Among the phosphorylation sites, phosphorylation on serine was observed more frequently than threonine or tyrosine, which is keeping with their relative abundance in cells (Fig. 3B). We then compared the phosphosites identified in this study against PhosphoSitePlus and PhosphoNET databases to check whether the identified phosphosites have been previously reported. Notably, out of 149 phosphosites (155 phosphopeptides) identified from both cell lines, ~ 80% of the phosphosites were previously reported in these databases. Only 31 phosphosites (~ 20%) were previously not reported to be phosphorylated, and among these peptides, ~ 35% were predicted to be phosphorylated by P-Site Predictor algorithm (PhosphoNET). Further studies will be needed to characterize these novel phosphosites both in the context of signaling mechanisms and the immunopeptidome.

Similarly, 51 and 81 peptides were identified with deamidation modification in A375 and Loucy cell lines and only 3 peptides were common between these cell lines. One of the common peptides, KLNDTYVNV, contained deamidation modification at P3 position in both cell lines. Though deamidation modification can occur as an experimental artifact, deglycosylation of asparagine residue catalyzed by NGLY1 enzyme also leads to deamidation (Misaghi et al. 2004). Out of the 129 deamidated peptides identified from both cell lines, 57 peptides (~ 44%) have deamidation modification localized to *N*-glycosylation motif *N*-X(P)-S/T indicating deglycosylation of peptides before presentation by MHC molecules. Further gene ontology analysis of the proteins with deamidation sites indicated these proteins localize to different membrane compartments in the cell. Despite the deep coverage of the immunopeptidome, other modifications, such as lysine acetylation and lysine methylation, were found to be very low in our data sets. We identified 4 and 3 lysine-acetylated, 5 and 9 lysine-methylated peptides in A375 and Loucy cell lines, respectively. Further fractionation of the enriched MHC-bound peptides can increase the depth of identifying these post-translationally modified peptides.

### Localization of PTMs at P3–P8 position in the MHC-bound peptide

Next, we looked at the frequency of the phosphorylation and deamidation modifications at different amino acids along the length of MHC-bound peptides. We observed that these modifications were enriched on P3–P8 amino acid positions excluding the MHC class I binding anchor positions P2 and P9. This suggests that modification at binding anchor positions would interfere with binding to MHC molecules. Notably, we observed that the phosphorylation modification is strongly localized at P4 position in 93 peptides out of 155 phosphorylated peptides identified in both A375 and Loucy cell lines (Fig. 3C). Considering the amino acid properties in the MHC binding motif, the selectivity of phosphorylation modification at P4 is probably due to higher localization of acidic amino acids at this position in the respective MHC allele binding motifs. Similarly, deamidation modification is highly localized at P3 to P7 positions with P3 as the commonest position of deamidation in most peptides (Fig. 3D). Out of 129 deamidated peptides identified across both cell lines, 104 peptides were deamidated at positions P3–P7. Even though the number of peptides with acetylation and methylation was small, we observed that a majority of these have the modification localized at P4 position. As P3–P8 position of the peptide is protruding out of the MHC binding pocket and interacting with the T-cell receptor, high frequency of the PTMs at these locations indicates a possible role of interaction with the T-cell receptor.

### Identification of spliced peptides through de novo sequencing pipeline

Identification of spliced peptides poses another challenge due to non-genome encoded, post-translational de novo assembly of these sequences catalyzed by the proteasome. In addition, due to unpredictable branching points of the splicing reaction, identification of spliced peptides has been performed using de novo sequencing of the MS/MS spectra as described by Mylonas et al. (2018). Peptides identified by PEAKS de novo sequencing pipeline were filtered for − 10log*P* score of > 15 and average local confidence score of > 80%. Further processing of these peptides was performed using an in-house pipeline which identifies the splicing position of the de novo peptide by matching the fragments of the peptide against the human protein sequences. Furthermore, single amino acid spliced variants were removed from the final list of spliced peptides due to possibility of single nucleotide variants, or improper fragmentation of the peptides leading to loss of b1 or y1 ions. Our analysis focused on the identification of only cis-spliced peptides due to the controversial nature of the existence of trans-spliced peptides.

**Fig. 4:**
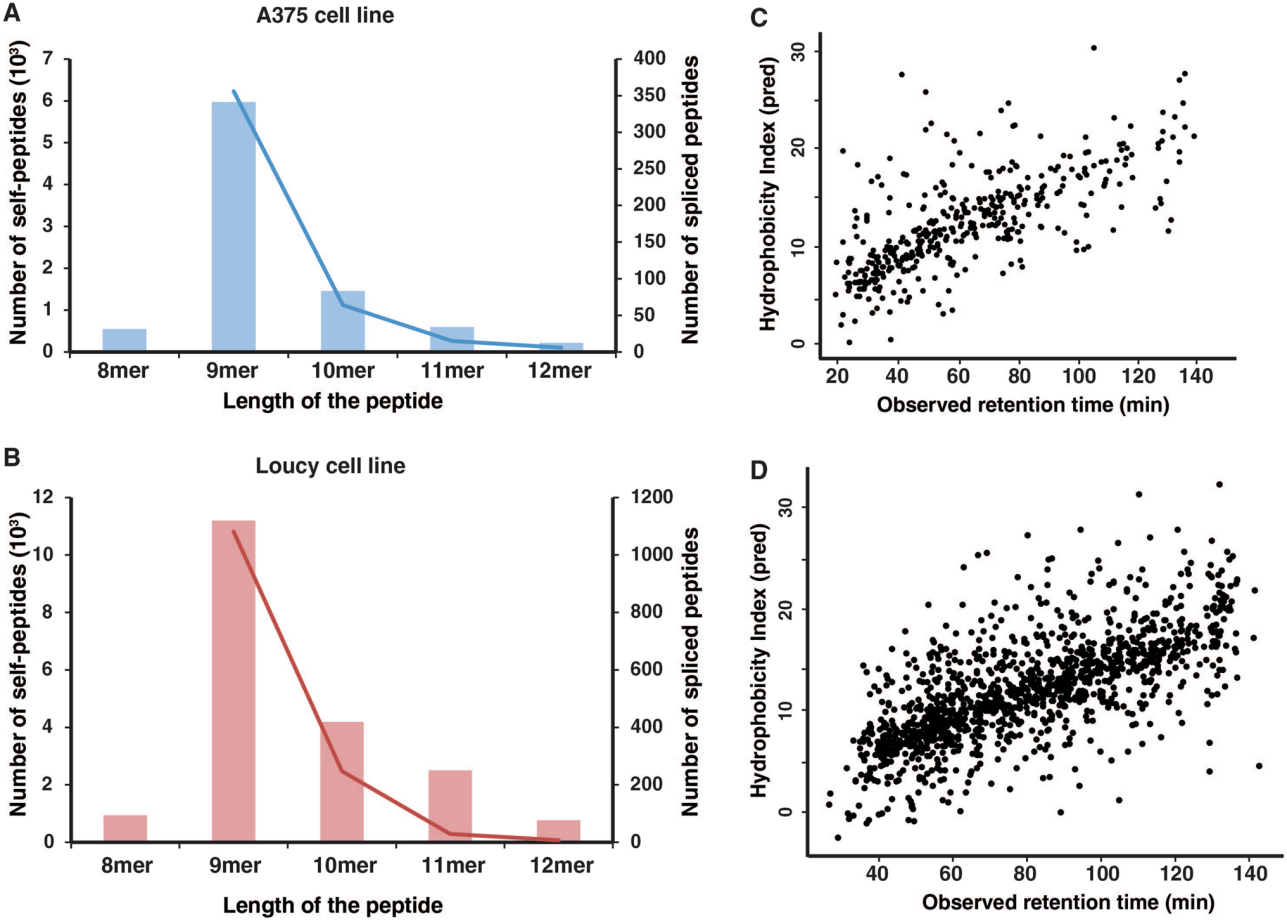
Identifications of spliced peptides. **A**, **B** Bar graph of the total number of identified MHC-bound peptides and spliced peptides in A375 and Loucy cell lines. **C**, **D** Scatter plots of the predicted hydrophobicity index (HI) calculated using SSRCalc and observed retention times (RT) of the spliced peptides. As the peptides are separated based on their hydrophobicity on reverse-phase resin-based separation columns, a positive correlation of HI with peptide retention time indicates the reliability of the peptide sequence assignment

Overall, we identified 415 and 1298 spliced peptides from A375 and Loucy representing ~ 5–7% of the MHC-bound peptides (Supplementary Tables 5–6). The relative contribution of the different lengths of the spliced peptides in both the cell lines followed similar trend to the non-spliced peptides identified with more 9-mer than 10-mer peptides (Fig. 4A, B). Our findings are in agreement with a previous report on the contribution of spliced peptides to the immunopeptidome (Mylonas et al. 2018). We further analyzed the spliced peptide sequence assignments by calculating the sequence-specific hydrophobicity index and correlated with the observed retention time of the spliced peptides. As expected, retention times were positively correlated with the predicted hydrophobicity indices indicating that majority of them are confident identifications (Fig. 4C, D). In addition, binding affinity predictions of these spliced peptides against MHC alleles of A375 and Loucy cell lines predicted ~ 50% of these peptides to be strong binders. To validate the identity of these peptides, raw data files were searched against combined human and spliced peptide databases and database searching was performed using similar settings as the original search. The identified peptides were filtered for 1% PSM-level false discovery rate. 234 out of 415 peptide sequences and 446 out of 1298 peptides were identified in A375 and Loucy, respectively. The missed identification of several peptides is probably due to low scoring of these identifications which fell below the 1% PSM-level FDR criteria.

### Validation of post-translationally modified peptides using synthetic peptides

**Fig. 5:**
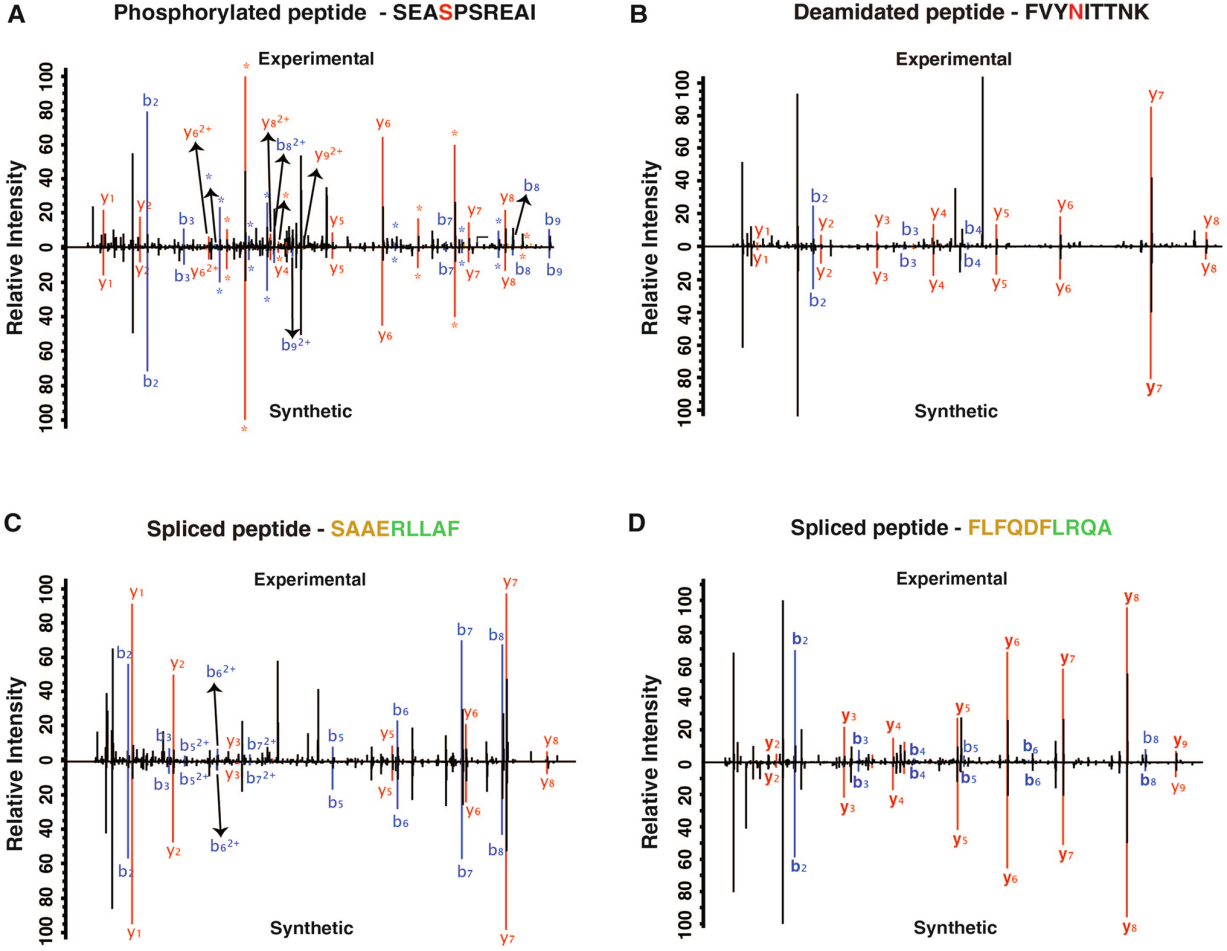
Validation of the identified peptides using synthetic peptide. Synthetic peptides were synthesized and analyzed by LC–MS/MS analysis. Peptides were validated by overlaying the experimental MS/MS spectrum with the synthetic MS/MS spectrum. Representative MS/MS spectrum was shown for **A** SEA**S**PSREAI (phosphorylated peptide), **B** FVY**N**ITTNK (deamidated peptide) and **C**, **D** spliced peptides (SAAERLLAF, FLFQDFLRQA)

Synthetic standard peptides were prepared to validate the identification of both post-translationally modified peptides and spliced peptides. We selected a total of 75 peptides for validation including 26 phosphorylated peptides, 10 peptides with deamidation modification and 39 spliced peptides (Supplementary Table 7). Synthetic peptides were analyzed by LC–MS/MS analysis on Orbitrap Eclipse mass spectrometer. MS/MS spectra of the synthetic peptides showed good correlation to the experimental spectrum for 25 phosphorylated, 10 deamidated and 34 spliced peptides validating the identification of these post-translationally modified MHC-bound peptides (Supplementary Fig. 2). Overlaid MS/MS spectrum of experimentally derived and synthetic peptides for SEASPSREAI (phosphorylated peptide), FVYNITTNK (deamidated peptide) and spliced peptides (SAAERLLAF, FLFQDFLRQA) are shown in Fig. 5.

## Conclusions

In this study, we profiled the post-translational landscape of the MHC class I immunopeptidome from 2 cell lines, i.e., A375 and Loucy using a highly sensitive mass spectrometer. Our results showed that peptides with post-translational modifications constitute ~ 1.5% of the total number of MHC-bound peptides. Gene ontology analysis and enrichment of N–X–S/T motif of the deamidated peptides suggests that these are indeed derived from glycosylated proteins. Excluding possible experimental artifacts, a fraction of these deamidated peptides could reveal novel glycosylation motifs as only 44% of the deamidated peptides identified in this study belonged to the known N-glycosylation motif of N–X–S/T. A recent study has shown that selective inhibition of the N-glycanase (PNGase) leads to a reduction in the levels of deamidated peptides among enriched MHC-bound peptides (Mei et al. 2020). Though only a small number of other modifications were identified, our results indicate a diverse PTM footprint of the MHC class I immunopeptidome. Across the different post-translational modifications, several peptides have modifications localized to P3–P7 positions possibly playing an important role in forming a stable TCR–peptide–MHC complex during the interaction with T-lymphocyte. Though phosphorylated and deamidated peptides were shown to have the potential to elicit the T-cell immune response (Zarling et al. 2006; Dalet et al. 2011), large scale characterization of diverse PTMs and their role in generating the immune response is very much needed to expand the scope of immunotherapy.

Proteasome-catalyzed peptide splicing (PCPS) further diversifies the peptides presented by MHC class I by producing non-contiguous peptides formed by combining two distant regions of a protein (Dalet et al. 2010). Identification of spliced peptides has been a challenging task as it is limited by the de novo sequencing capabilities and is further made complex by the possibility of improper fragmentation of precursor ions. Several studies have reported contrasting results on the contribution of spliced peptides using MHC class I immunopeptidome. For example, Liepe et al. (2016) and Faridi et al. (2018) have identified that spliced peptides contribute to ~ 30% of the MHC-bound peptides. Our findings that spliced peptides constitute ~ 5% of all MHC-bound peptides is supported by previous studies (Mylonas et al. 2018; Rolfs et al. 2019). Further filtering of the spliced peptides is possible as the source of these peptide sequences can also be derived from non-coding regions of the genome, falsely representing as spliced peptides. However, therapeutically, spliced peptide sequences pose a distinct advantage as they can be a unique source of non-self-antigens presented by MHC molecules crucial for the generation of immune response against the target cell.

In conclusion, our study demonstrated that enrichment of MHC–peptide complexes followed by mass spectrometry analysis enables an in-depth analysis of the immunopeptidome which is characterized by the presentation of several post-translationally modified peptides and spliced peptides under the huge background of non-modified peptides. Our study reiterates the need to further improve the methods, such as fractionation or enrichment of post-translational modifications to identify ultra-low stoichiometric PTMs.

## Supplementary Information

Below is the link to the electronic supplementary material.

Supplementary Figure 1: Overlap of the number of peptides identified in Sequest and Bolt search algorithms in (A) A375 malignant melanoma cell line (B) Loucy T-cell leukemia cell line (PDF 78 kb)

Supplementary Figure 2: Overlay of the synthetic and experimental MS/MS spectrum of the peptides validated using the synthetic peptides (PDF 3068 kb)

Supplementary file3 (XLSX 860 kb)

Supplementary file4 (XLSX 1156 kb)

Supplementary file5 (XLSX 1520 kb)

Supplementary file6 (XLSX 3222 kb)

Supplementary file7 (XLSX 223 kb)

Supplementary file8 (XLSX 266 kb)

Supplementary file9 (XLSX 11 kb)

## Data Availability

The mass spectrometry proteomics data have been deposited with the ProteomeXchange Consortium via the PRIDE partner repository with the data set identifier PXD024562.

## Abbreviations

MHC: Major Histocompatibility Complex
PTM: Post translational modifications
HCD: High energy collision-induced dissociation
PCPS: Proteosome-catalyzed peptide splicing

## References

[CR1] Andreatta M, Alvarez B, Nielsen M (2017). GibbsCluster: unsupervised clustering and alignment of peptide sequences. Nucleic Acids Res.

[CR2] Bassani-Sternberg M, Braunlein E, Klar R, Engleitner T, Sinitcyn P, Audehm S, Straub M, Weber J, Slotta-Huspenina J, Specht K, Martignoni ME, Werner A, Hein R, Busch HD, Peschel C, Rad R, Cox J, Mann M, Krackhardt AM (2016). Direct identification of clinically relevant neoepitopes presented on native human melanoma tissue by mass spectrometry. Nat Commun.

[CR3] Buonaguro L, Petrizzo A, Tornesello ML, Buonaguro FM (2011). Translating tumor antigens into cancer vaccines. Clin Vaccine Immunol.

[CR4] Dalet A, Vigneron N, Stroobant V, Hanada K, Van den Eynde BJ (2010). Splicing of distant peptide fragments occurs in the proteasome by transpeptidation and produces the spliced antigenic peptide derived from fibroblast growth factor-5. J Immunol.

[CR5] Dalet A, Robbins PF, Stroobant V, Vigneron N, Li YF, El-Gamil M, Hanada K, Yang JC, Rosenberg SA, Van den Eynde BJ (2011). An antigenic peptide produced by reverse splicing and double asparagine deamidation. Proc Natl Acad Sci USA.

[CR6] Engelhard VH, Altrich-Vanlith M, Ostankovitch M, Zarling AL (2006). Post-translational modifications of naturally processed MHC-binding epitopes. Curr Opin Immunol.

[CR7] Faridi P, Li C, Ramarathinam SH, Vivian JP, Illing PT, Mifsud NA, Ayala R, Song J, Gearing LJ, Hertzog PJ, Ternette N, Rossjohn J, Croft NP, Purcell AW (2018). A subset of HLA-I peptides are not genomically templated: Evidence for cis- and trans-spliced peptide ligands. Sci Immunol.

[CR8] Krokhin OV, Spicer V (2009). Peptide retention standards and hydrophobicity indexes in reversed-phase high-performance liquid chromatography of peptides. Anal Chem.

[CR9] Liepe J, Marino F, Sidney J, Jeko A, Bunting DE, Sette A, Kloetzel PM, Stumpf MP, Heck AJ, Mishto M (2016). A large fraction of HLA class I ligands are proteasome-generated spliced peptides. Science.

[CR10] Linnemann C, van Buuren MM, Bies L, Verdegaal EM, Schotte R, Calis JJ, Behjati S, Velds A, Hilkmann H, Atmioui DE, Visser M, Stratton MR, Haanen JB, Spits H, van der Burg SH, Schumacher TN (2015). High-throughput epitope discovery reveals frequent recognition of neo-antigens by CD4+ T cells in human melanoma. Nat Med.

[CR11] Mei S, Ayala R, Ramarathinam SH, Illing PT, Faridi P, Song J, Purcell AW, Croft NP (2020). Immunopeptidomic analysis reveals that deamidated HLA-bound peptides arise predominantly from deglycosylated precursors. Mol Cell Proteomics.

[CR12] Misaghi S, Pacold ME, Blom D, Ploegh HL, Korbel GA (2004). Using a small molecule inhibitor of peptide: N-glycanase to probe its role in glycoprotein turnover. Chem Biol.

[CR13] Mishto M, Liepe J (2017). Post-translational peptide splicing and T cell responses. Trends Immunol.

[CR14] Mylonas R, Beer I, Iseli C, Chong C, Pak HS, Gfeller D, Coukos G, Xenarios I, Muller M, Bassani-Sternberg M (2018). Estimating the contribution of proteasomal spliced peptides to the HLA-I ligandome. Mol Cell Proteomics.

[CR15] Neefjes J, Jongsma ML, Paul P, Bakke O (2011). Towards a systems understanding of MHC class I and MHC class II antigen presentation. Nat Rev Immunol.

[CR16] Reynisson B, Alvarez B, Paul S, Peters B, Nielsen M (2020). NetMHCpan-4.1 and NetMHCIIpan-4.0: improved predictions of MHC antigen presentation by concurrent motif deconvolution and integration of MS MHC eluted ligand data. Nucleic Acids Res.

[CR17] Robinson J, Guethlein LA, Cereb N, Yang SY, Norman PJ, Marsh SGE, Parham P (2017). Distinguishing functional polymorphism from random variation in the sequences of >10,000 HLA-A, -B and -C alleles. PLoS Genet.

[CR18] Rolfs Z, Solntsev SK, Shortreed MR, Frey BL, Smith LM (2019). Global Identification Of Post-Translationally Spliced Peptides With Neo-Fusion. J Proteome Res.

[CR19] Tran E, Ahmadzadeh M, Lu YC, Gros A, Turcotte S, Robbins PF, Gartner JJ, Zheng Z, Li YF, Ray S, Wunderlich JR, Somerville RP, Rosenberg SA (2015). Immunogenicity of somatic mutations in human gastrointestinal cancers. Science.

[CR20] Vigneron N, Ferrari V, Stroobant V, Abi Habib J, Van den Eynde BJ (2017). Peptide splicing by the proteasome. J Biol Chem.

[CR21] Yewdell JW, Schubert U, Bennink JR (2001). At the crossroads of cell biology and immunology: DRiPs and other sources of peptide ligands for MHC class I molecules. J Cell Sci.

[CR22] Zarling AL, Polefrone JM, Evans AM, Mikesh LM, Shabanowitz J, Lewis ST, Engelhard VH, Hunt DF (2006). Identification of class I MHC-associated phosphopeptides as targets for cancer immunotherapy. Proc Natl Acad Sci USA.

