# Supplementary figures and images for: Digging deeper into the immunopeptidome: characterization of post-translationally modified peptides presented by MHC I
Source: J Proteins Proteom. 2021 Jun 4;12(3):151–60. doi: 10.1007/s42485-021-00066-x (PMC9807509; doi:10.1007/s42485-021-00066-x)

# Supplementary Figure 1

1A.

A375 cell line

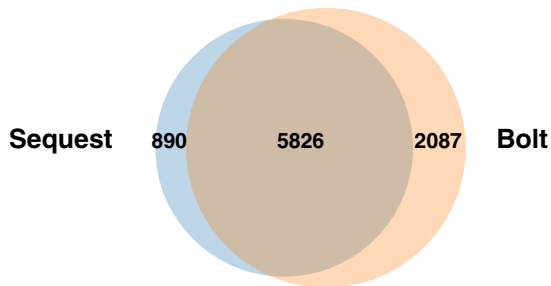

1B.

Loucy cell line

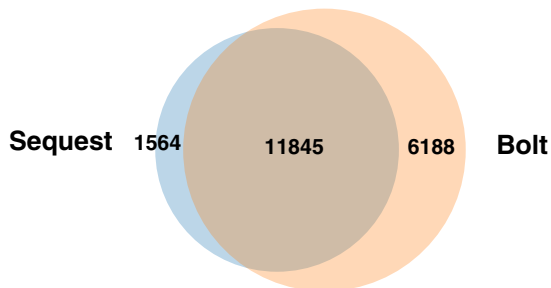

Supplement: Supplementary file 1 — Supplementary Figure 1: Overlap of the number of peptides identified in Sequest and Bolt search algorithms in (A) A375 malignant melanoma cell line (B) Loucy T-cell leukemia cell line (PDF 78 kb) [file 42485_2021_66_MOESM1_ESM.pdf]
