# Supplementary material for: Digging deeper into the immunopeptidome: characterization of post-translationally modified peptides presented by MHC I
Source: J Proteins Proteom. 2021 Jun 4;12(3):151–60. doi: 10.1007/s42485-021-00066-x (PMC9807509; doi:10.1007/s42485-021-00066-x)

Supplementary Figure 2

ILNGSDIRSLY – Deamidated

Synthetic

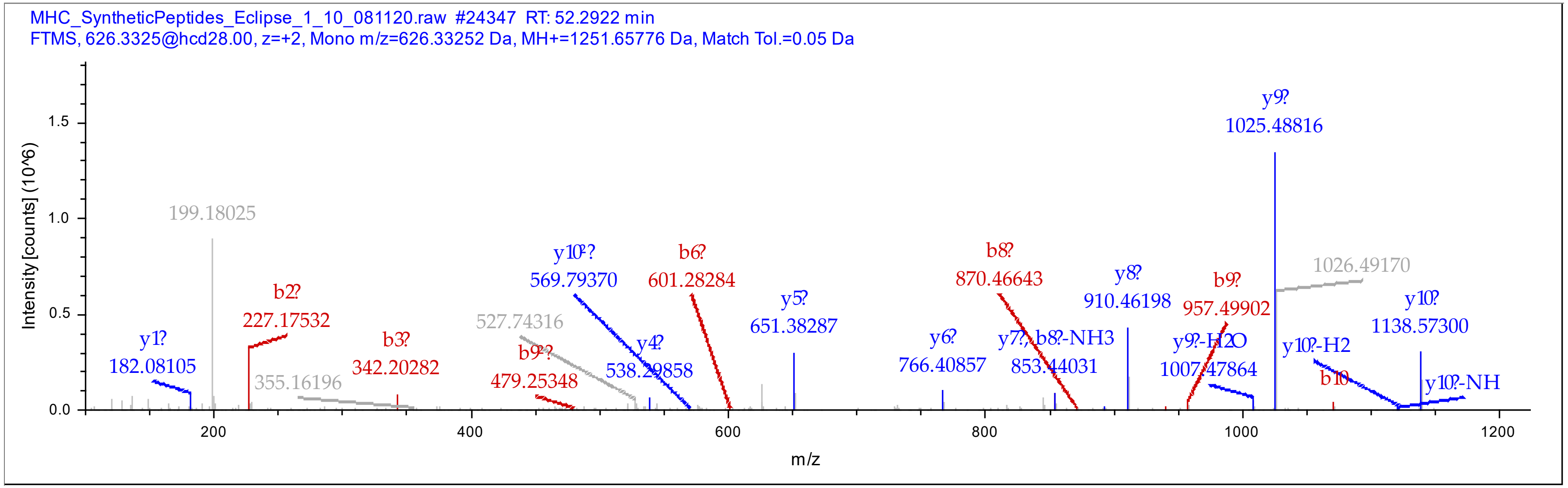

Experimental

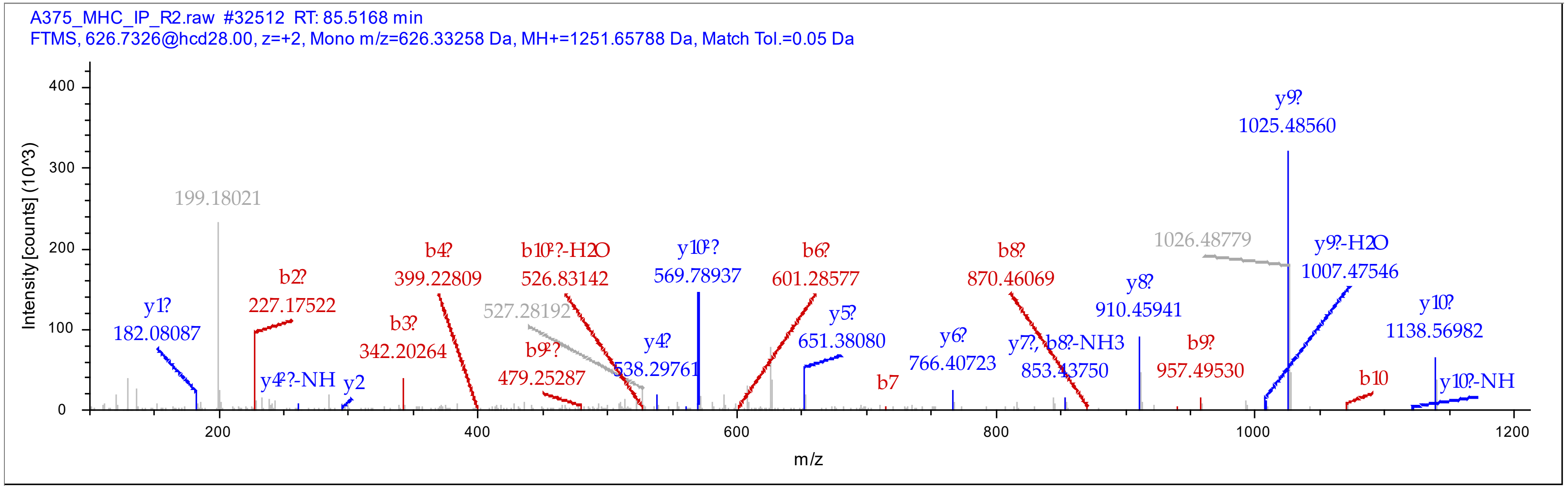

KLNDTYVNV – Deamidated

Synthetic

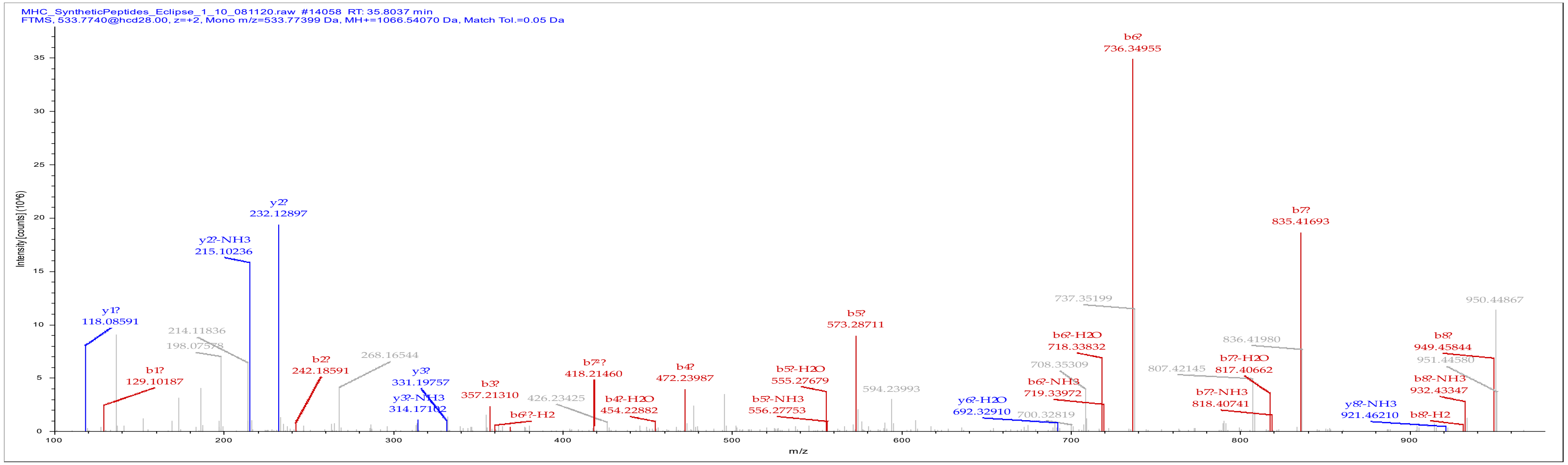

Experimental

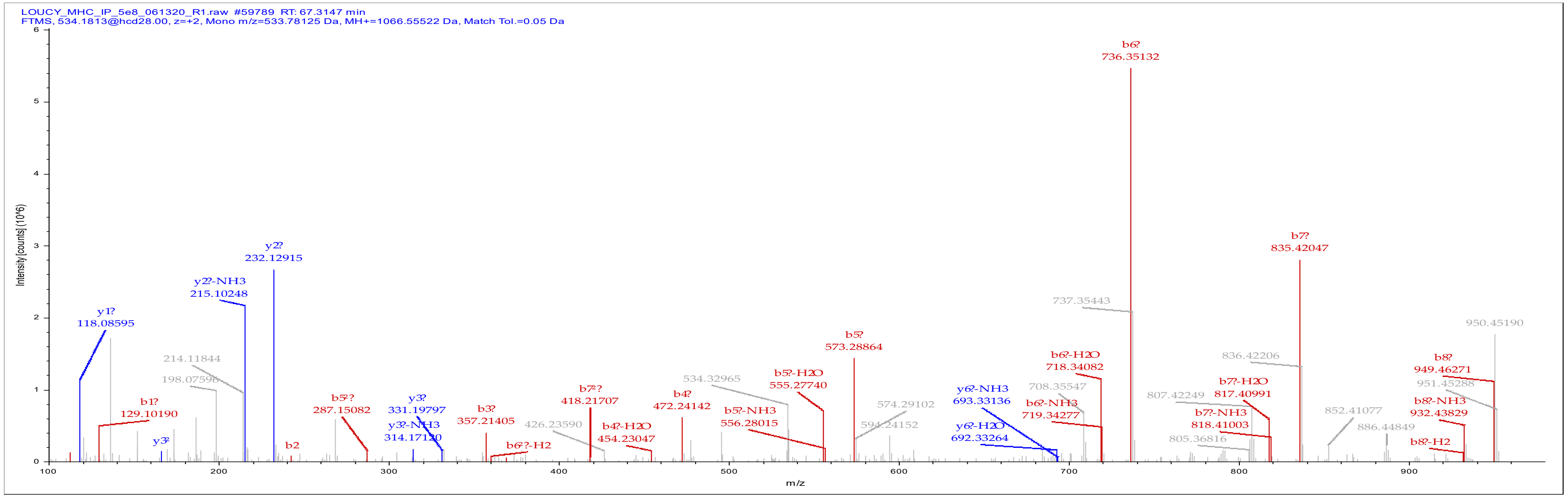

NLAENISRV – Deamidated

Synthetic

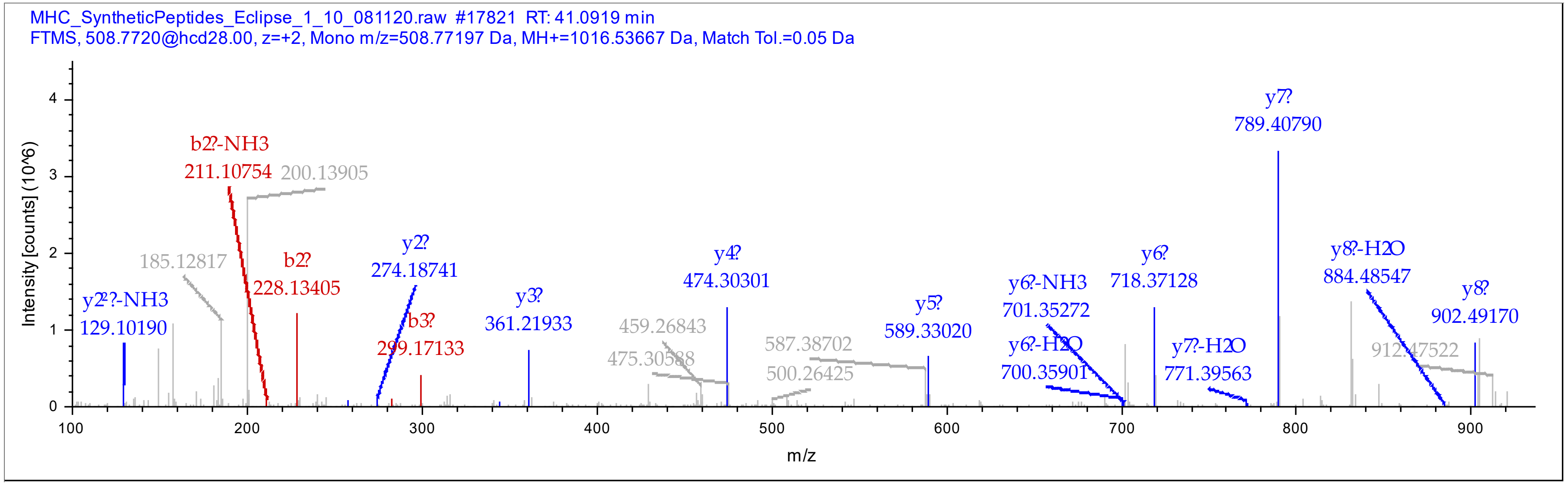

Experimental

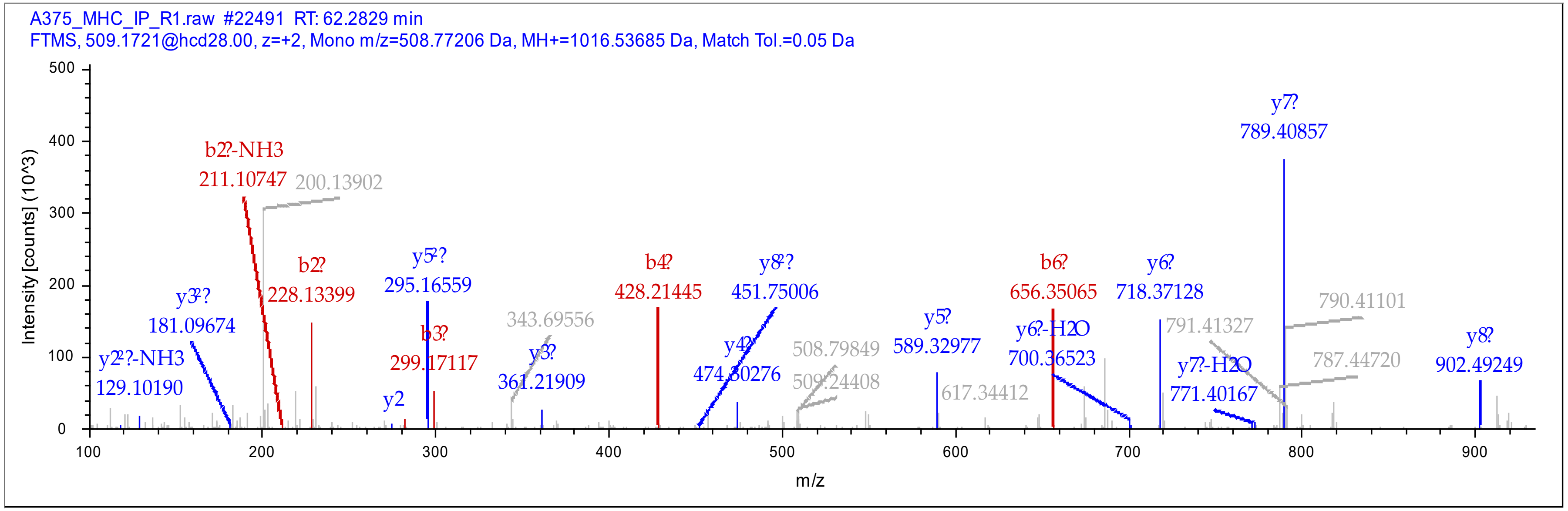

TSFKNSTKSW – Deamidated

Synthetic

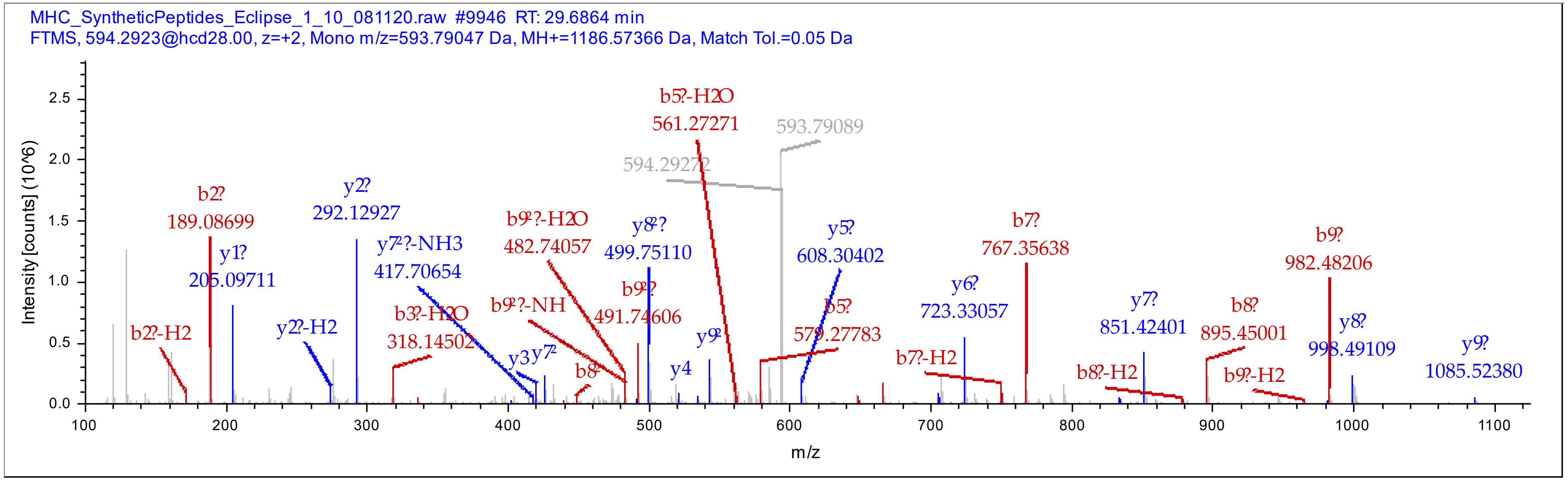

Experimental

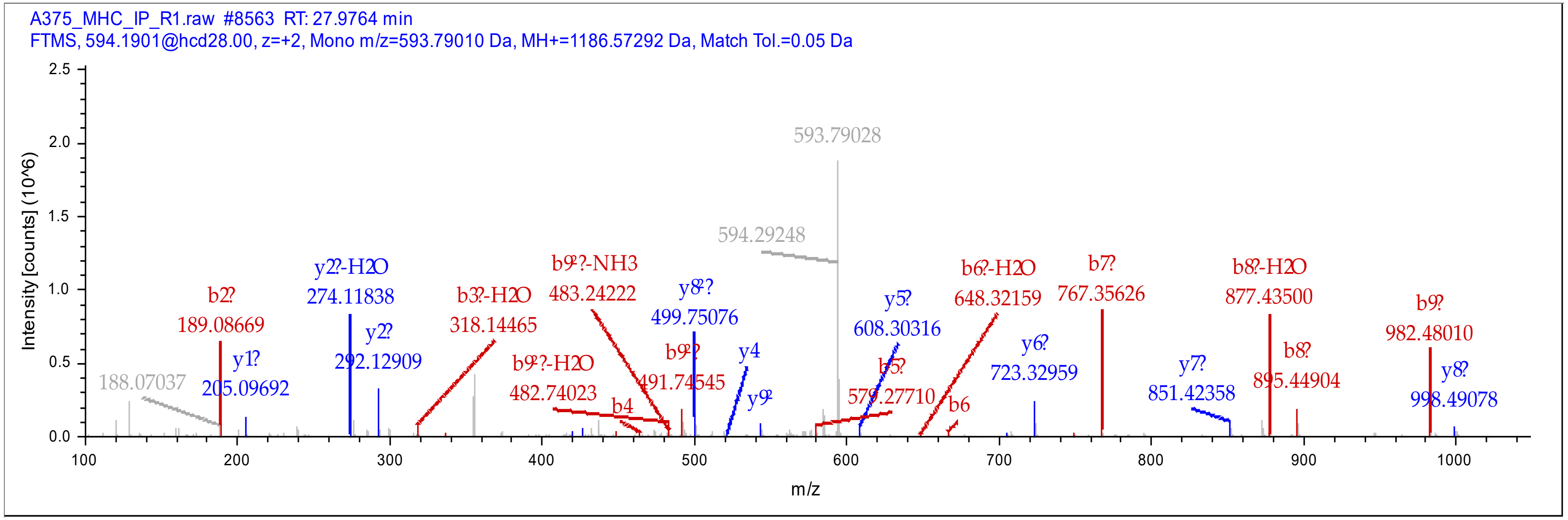

TTDTTNITKY – Deamidated

Synthetic

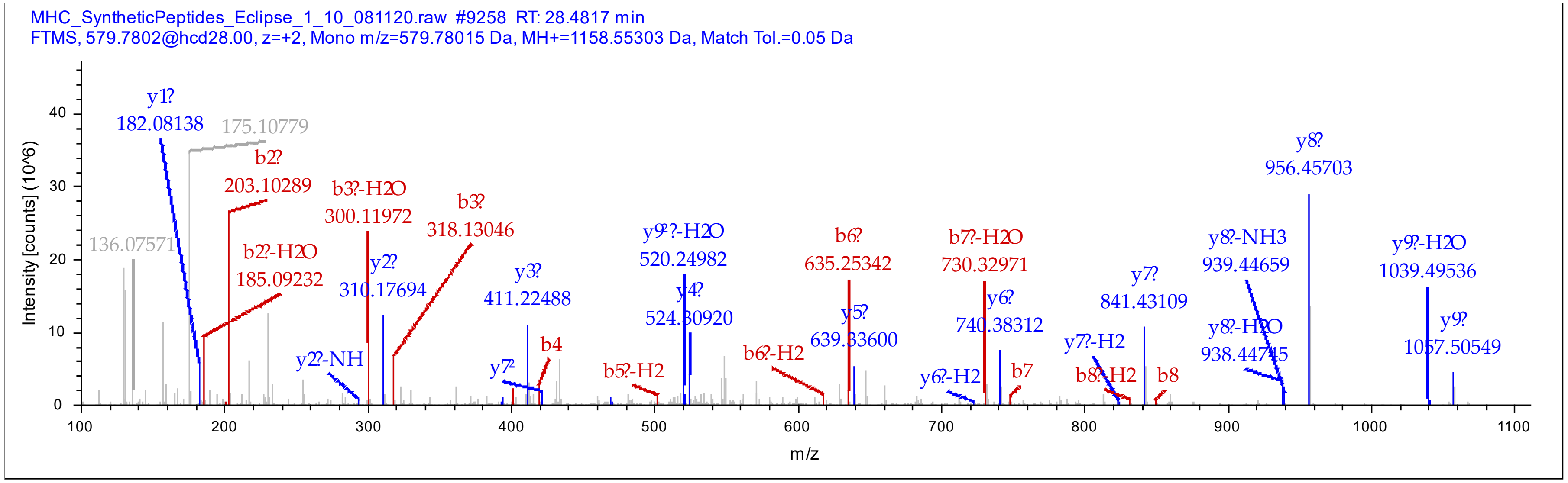

Experimental

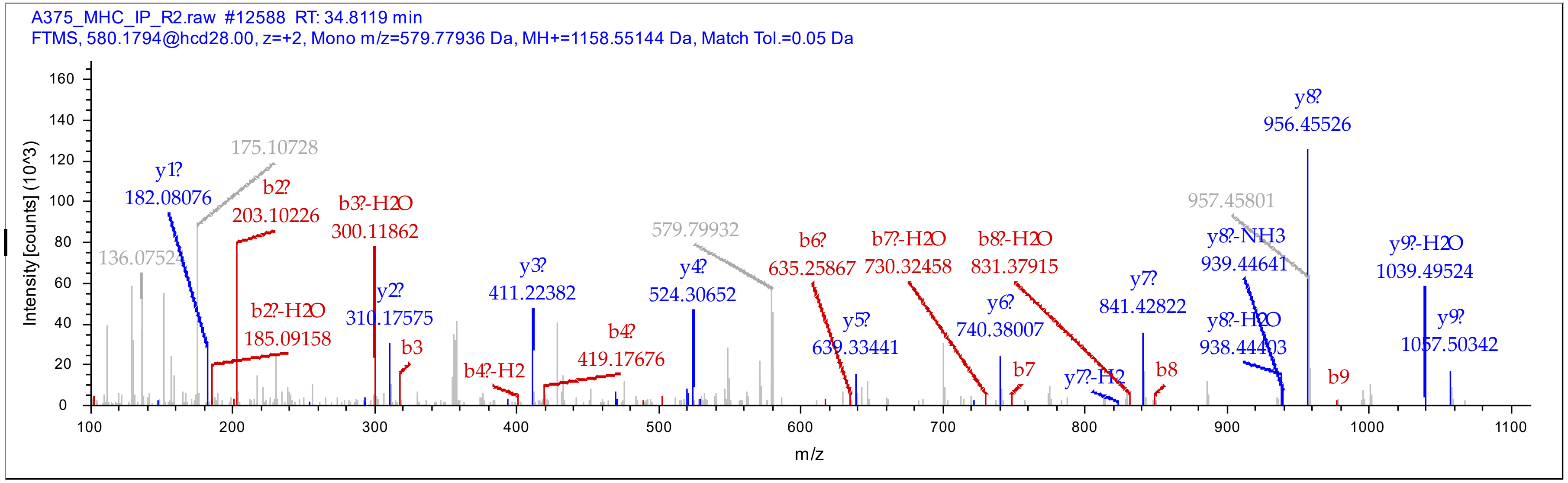

EELNLSGNKL – Deamidated

Synthetic

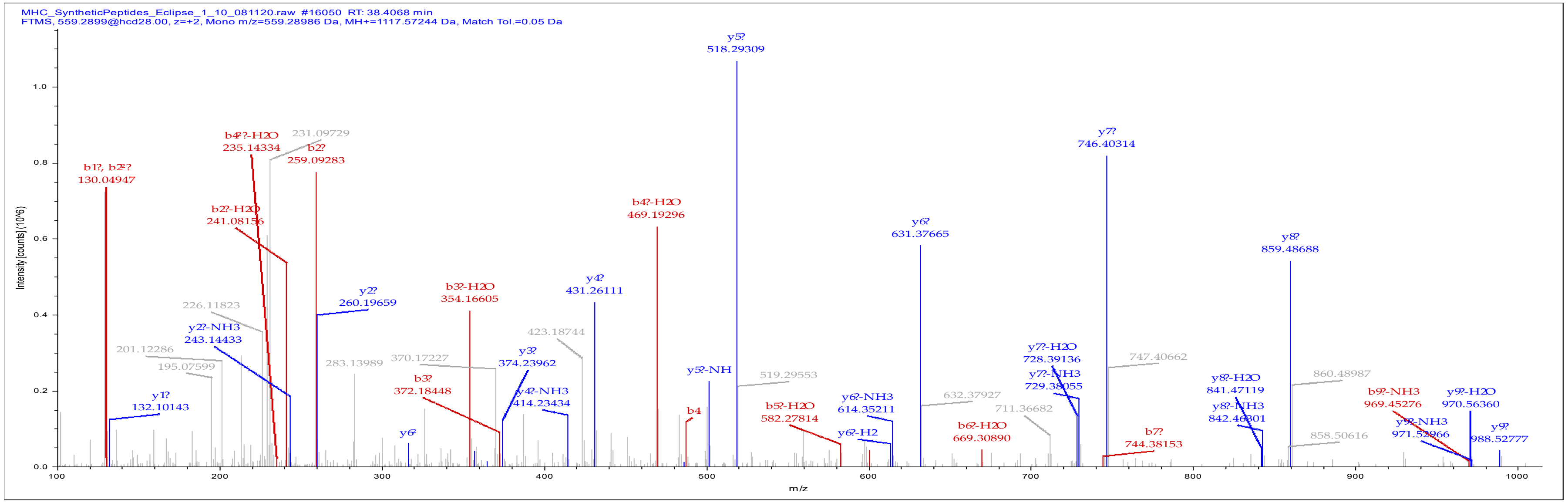

Experimental

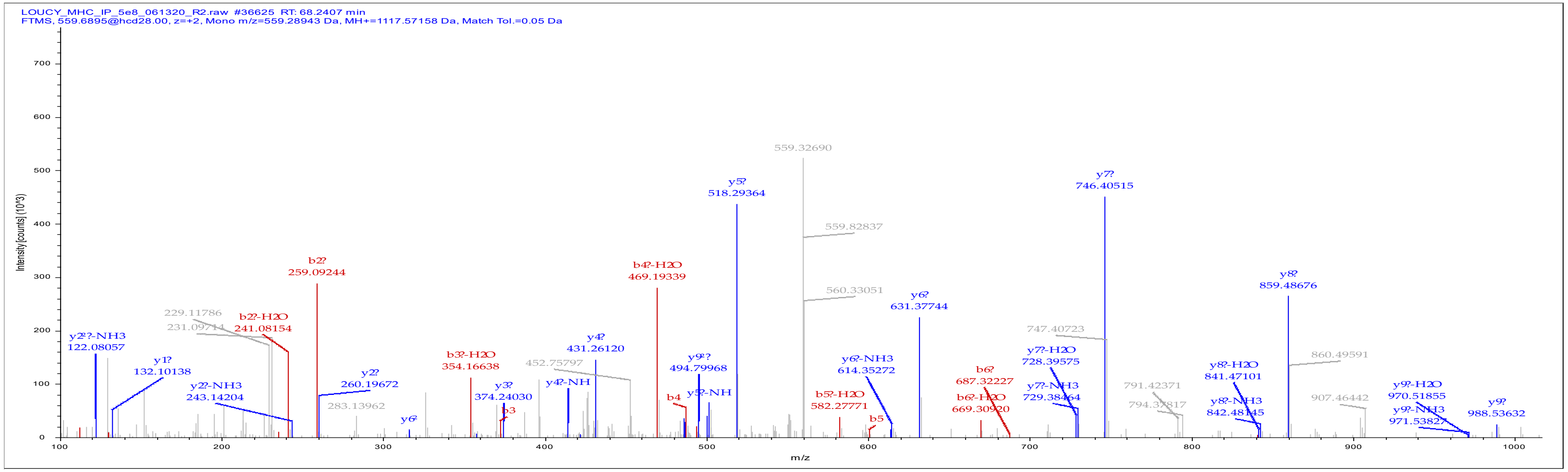

FVYNITTNK – Deamidated

Synthetic

Experimental

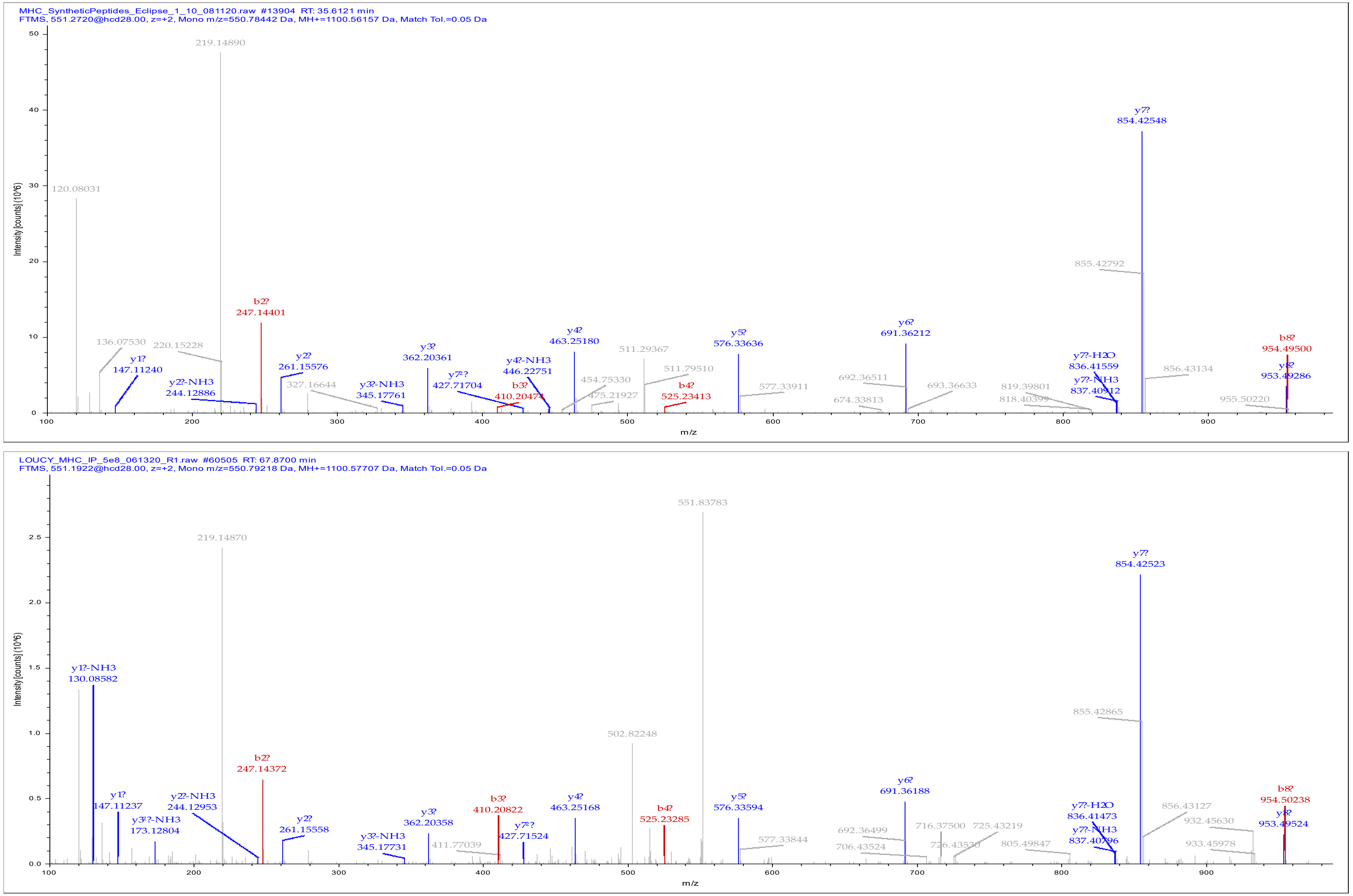

HTNATVSFL – Deamidated

Synthetic

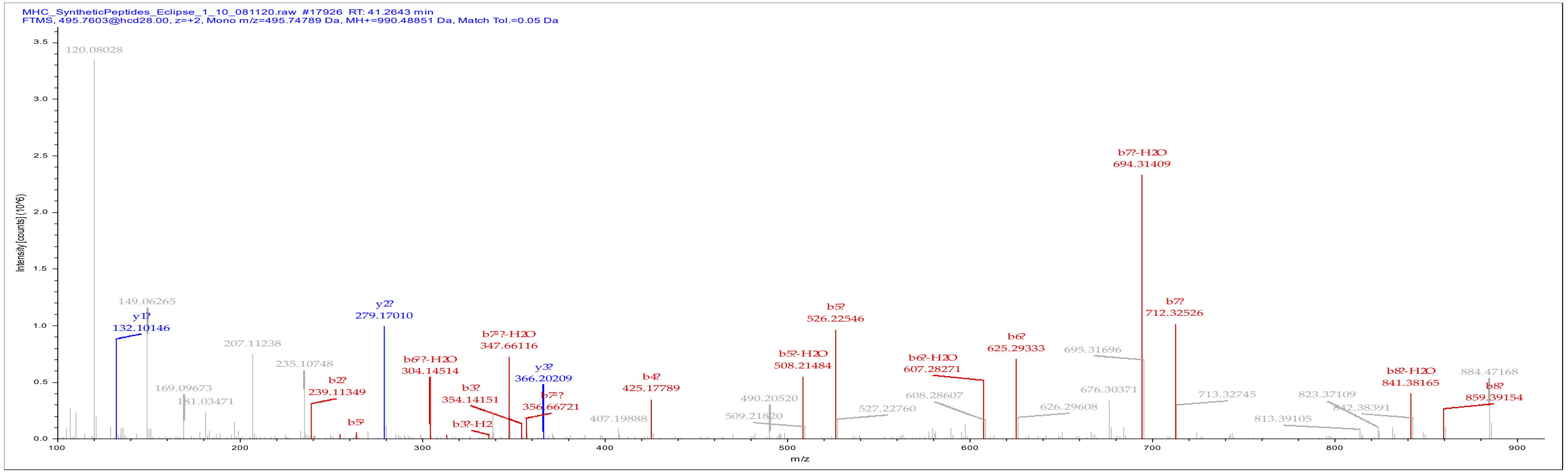

Experimental

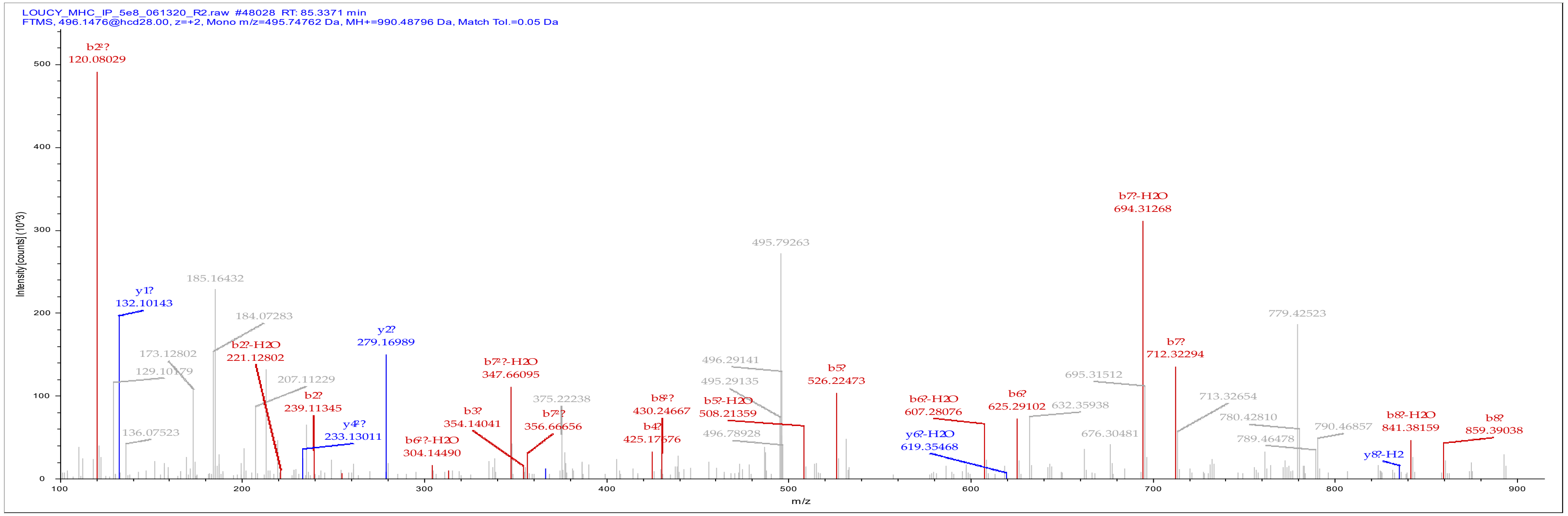

MPVEKNITL – Deamidated

Synthetic

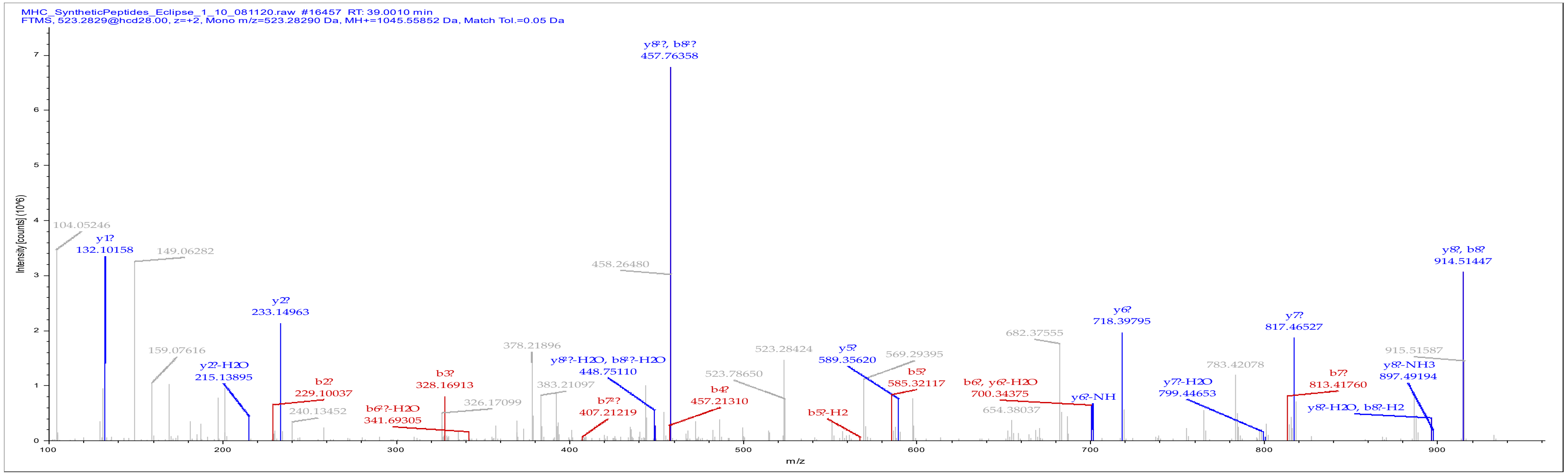

Experimental

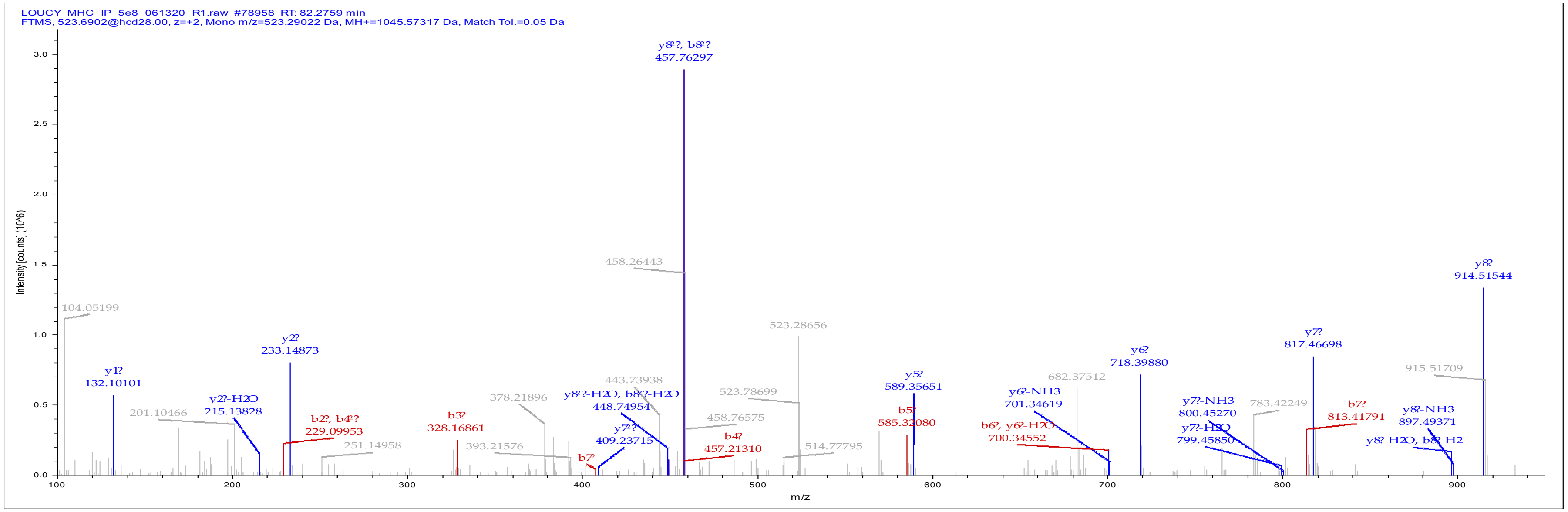

NESEYTVKW – Deamidated

Synthetic

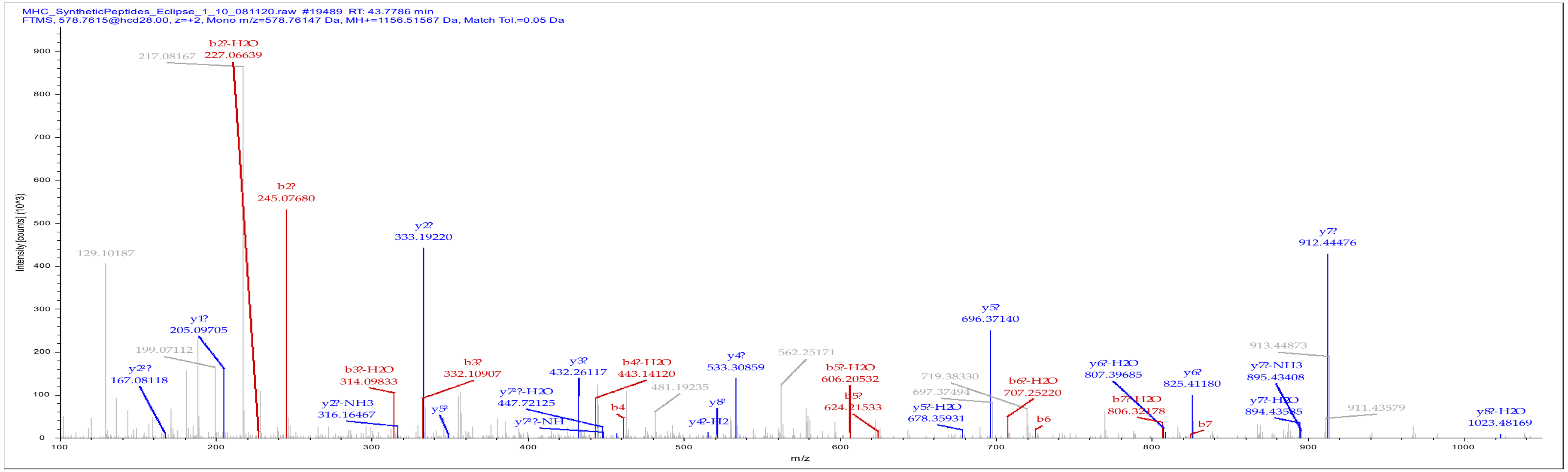

Experimental

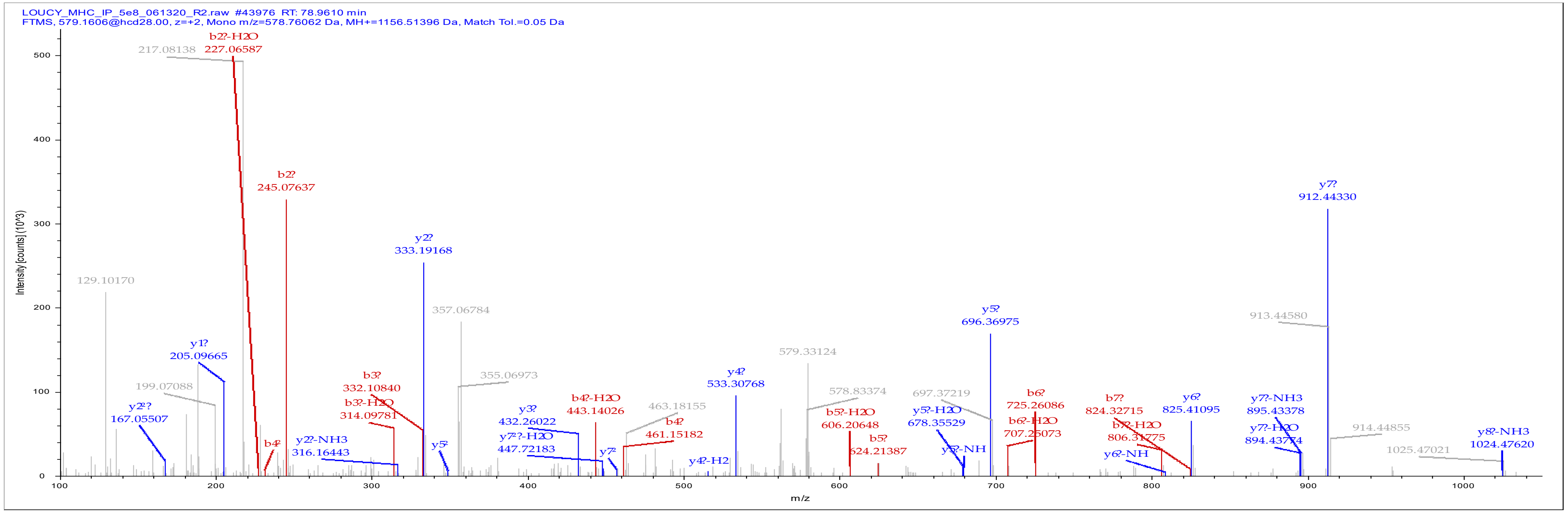

AENSPTRQQF – Phospho

Synthetic

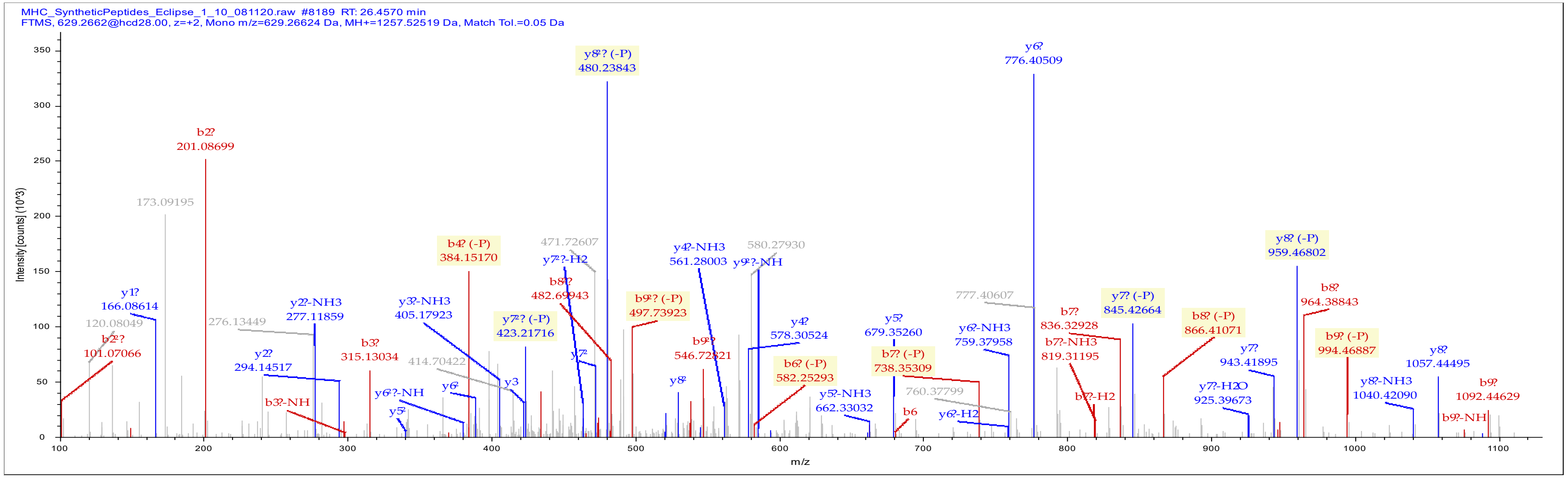

Experimental

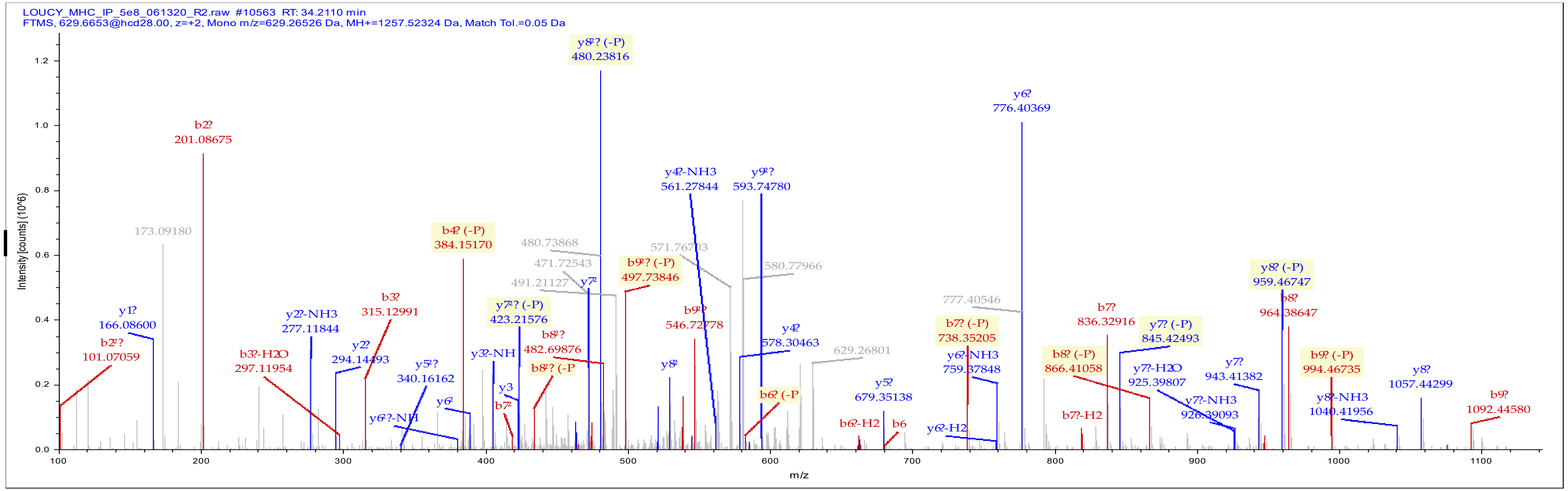

ATYTPQAPK – Phospho

Synthetic

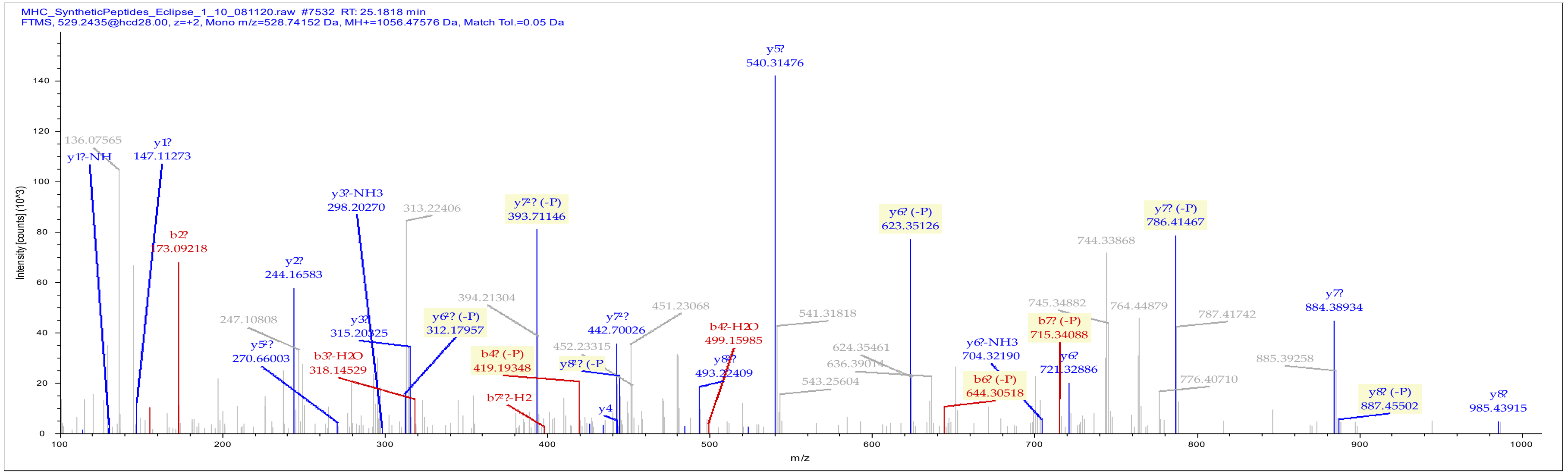

Experimental

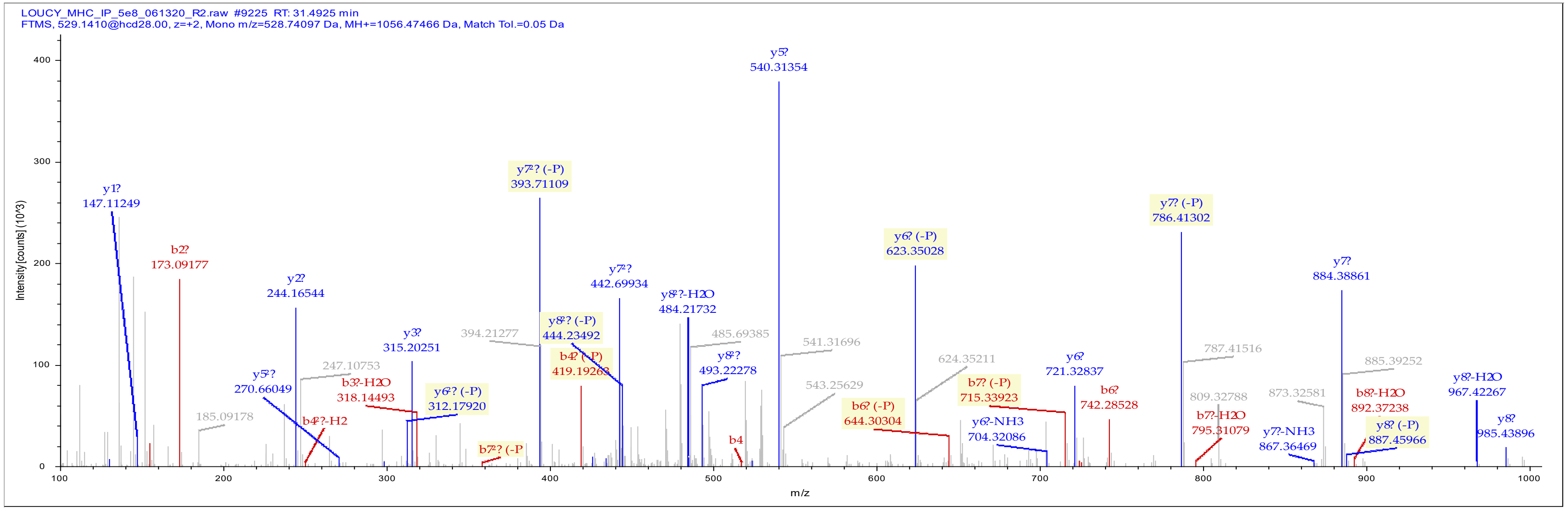

APVSPSSQKL – Phosspho

Synthetic

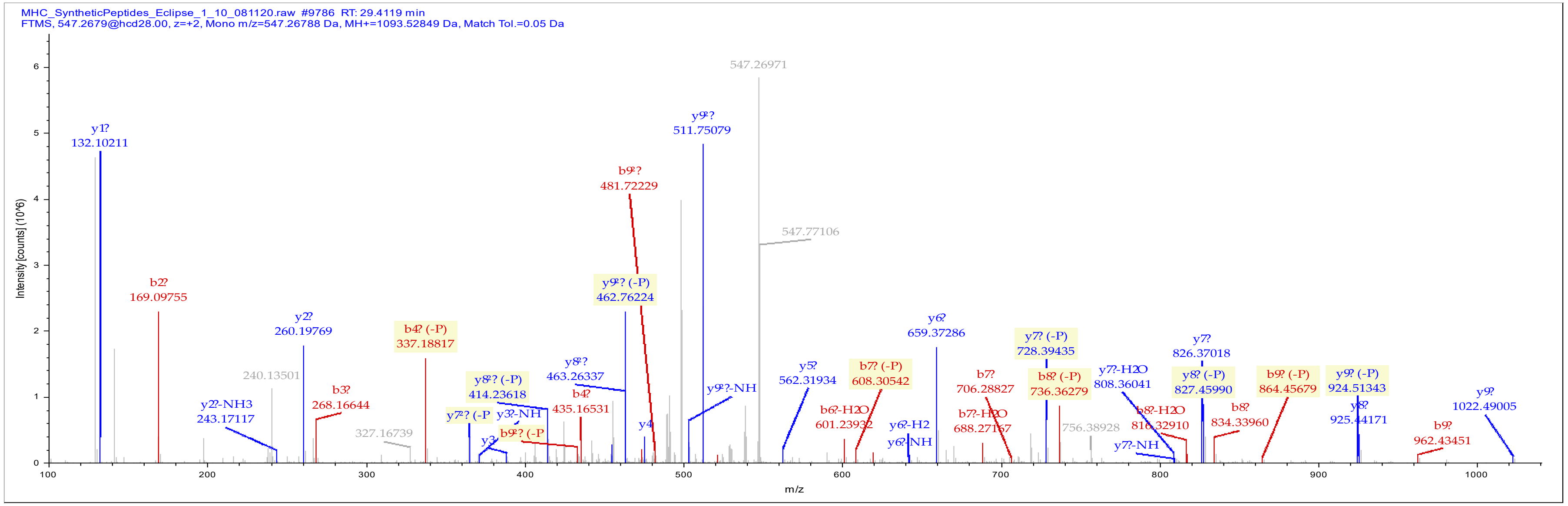

Experimental

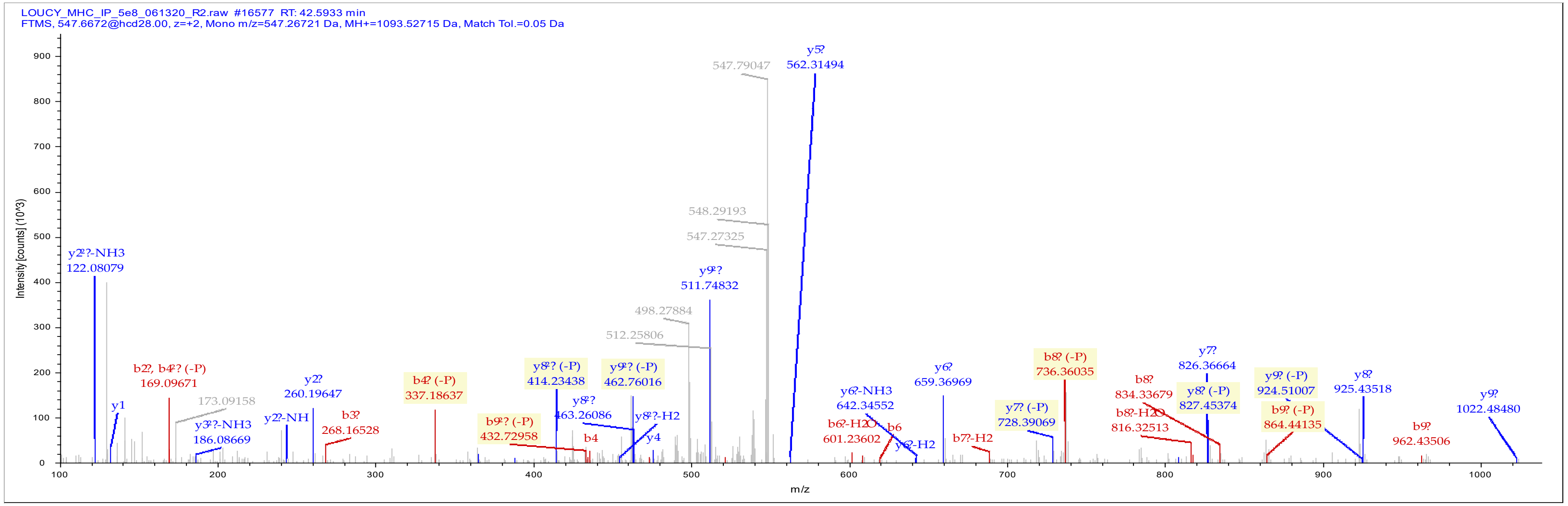

EELSPTAKF – Phospho

Synthetic

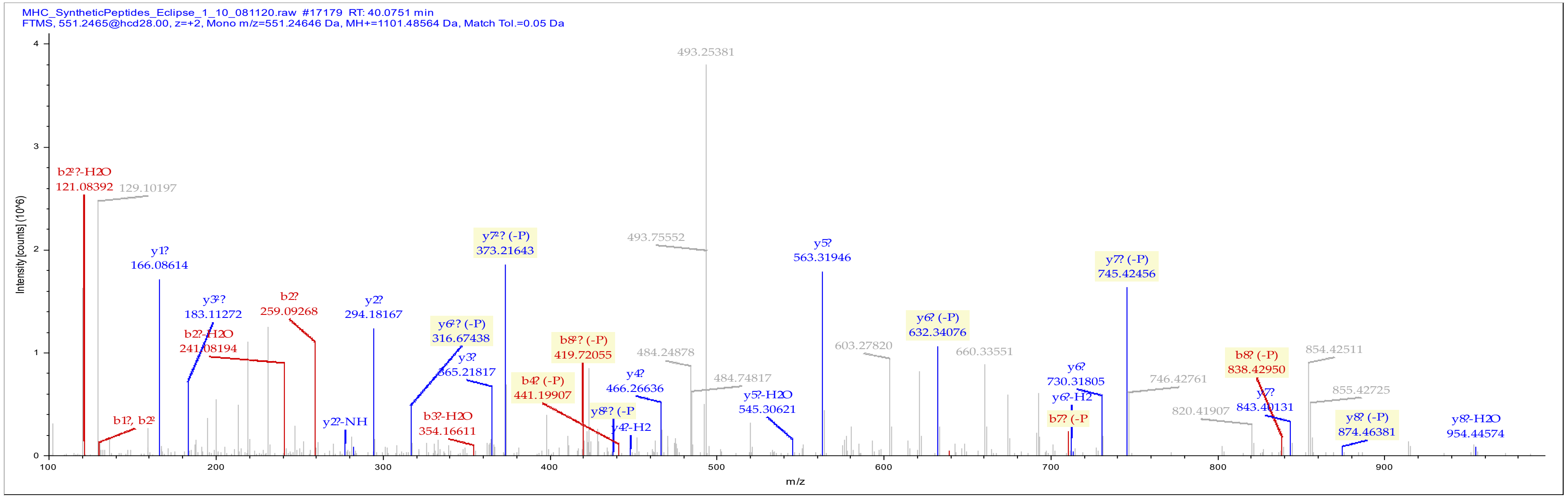

Experimental

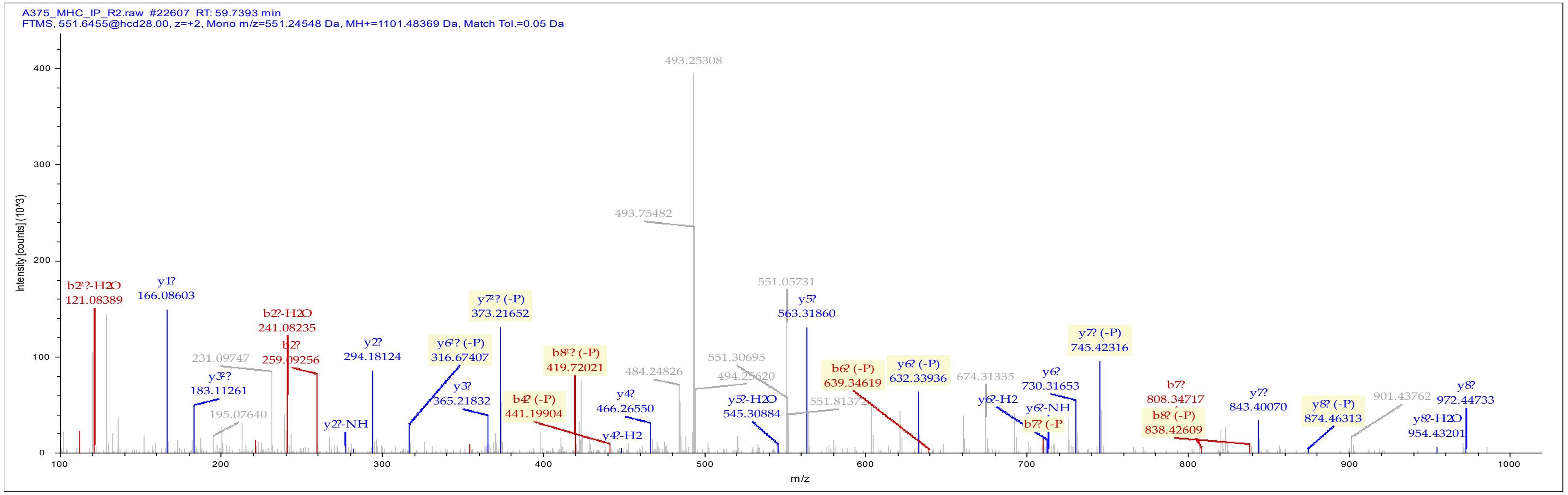

GENSGIGKLF – Phospho

Synthetic

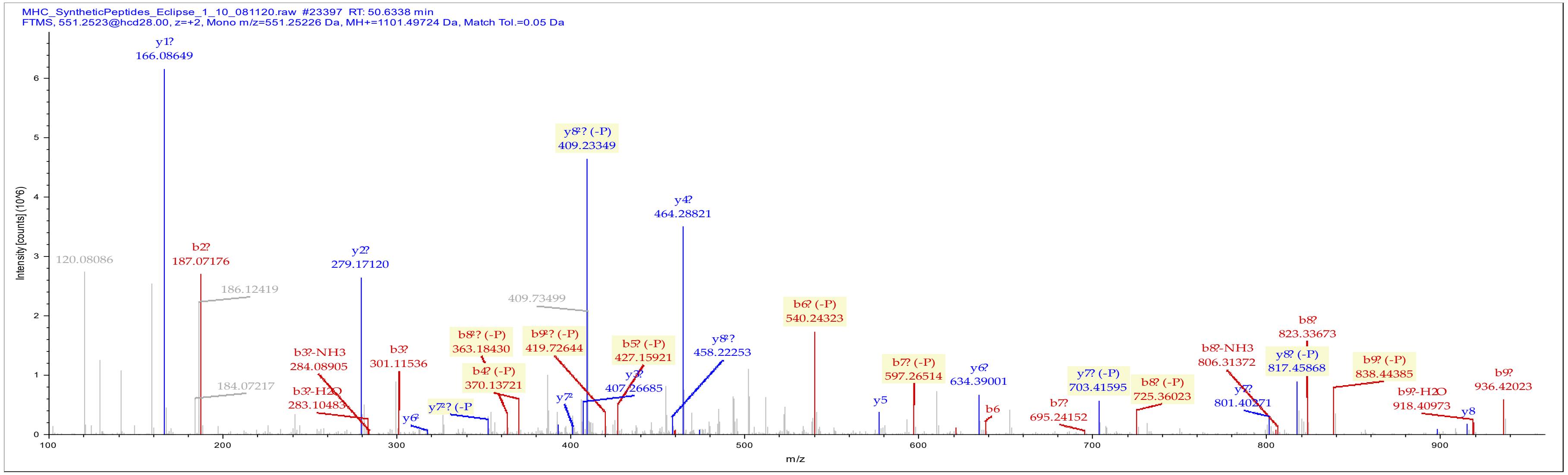

Experimental

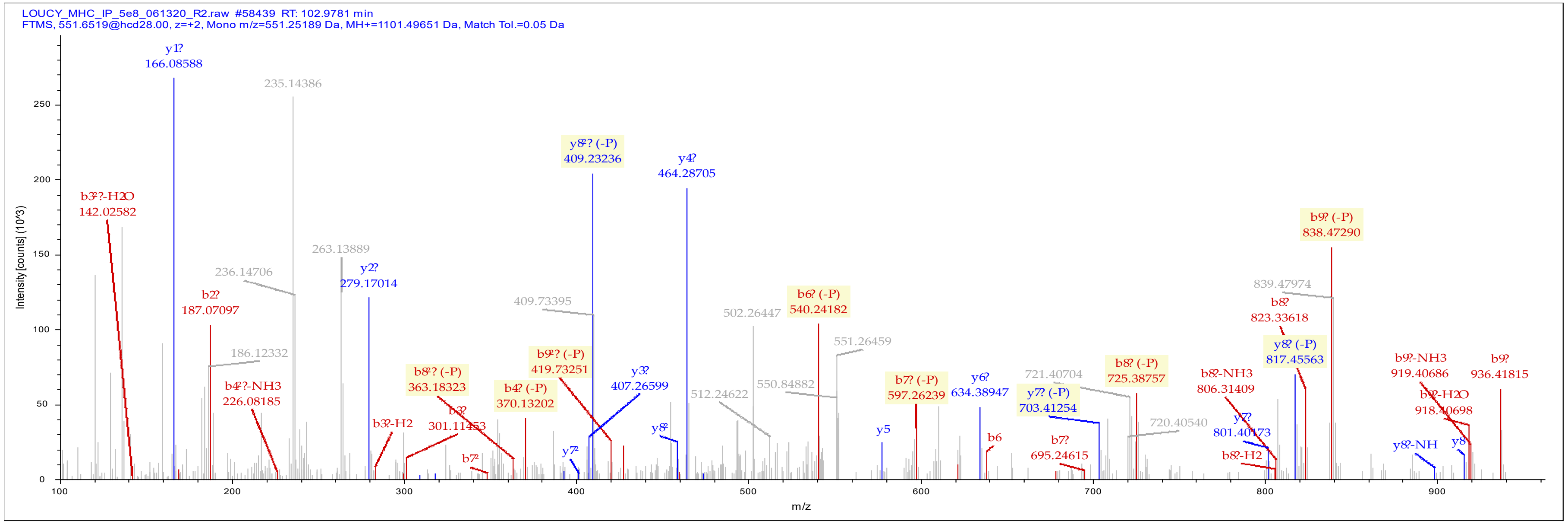

GEWSASLPHRF – Phospho

Synthetic

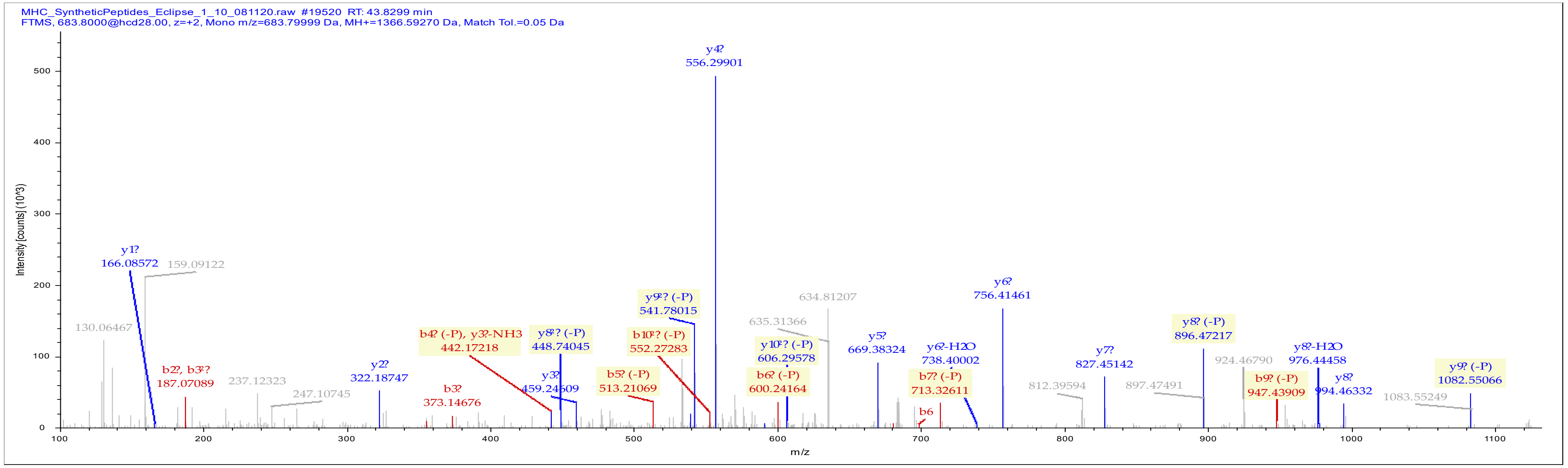

Experimental

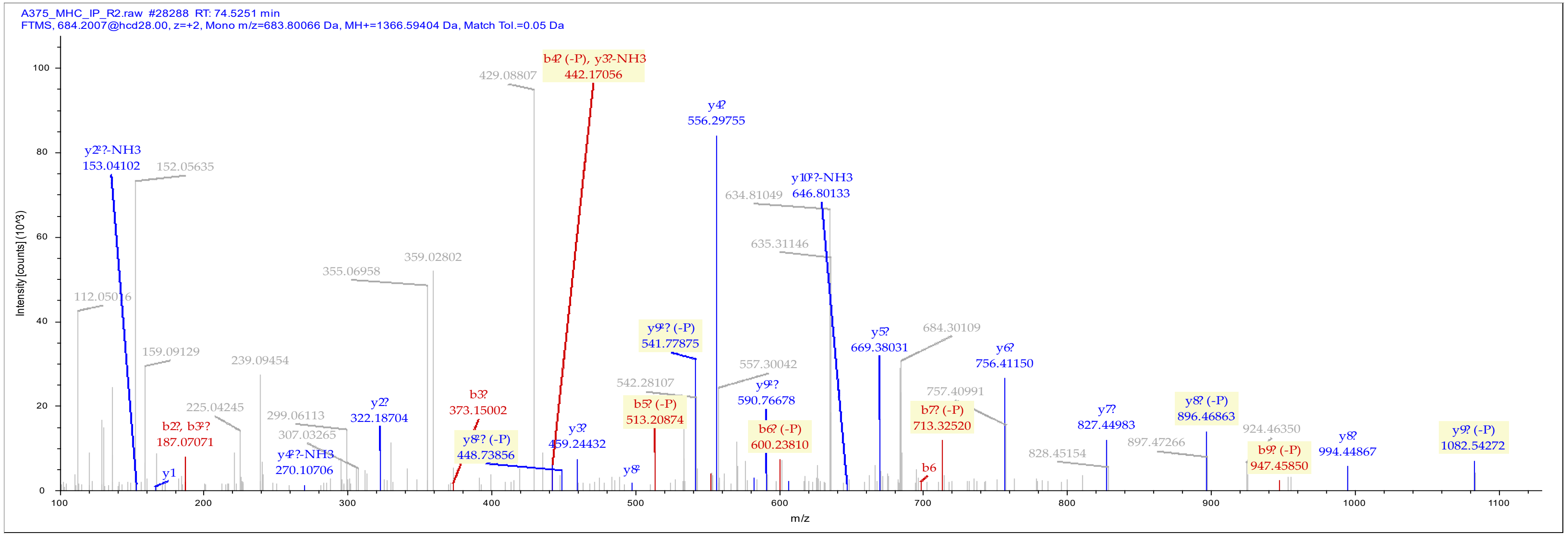

ITQGTPLKY – Phospho

Synthetic

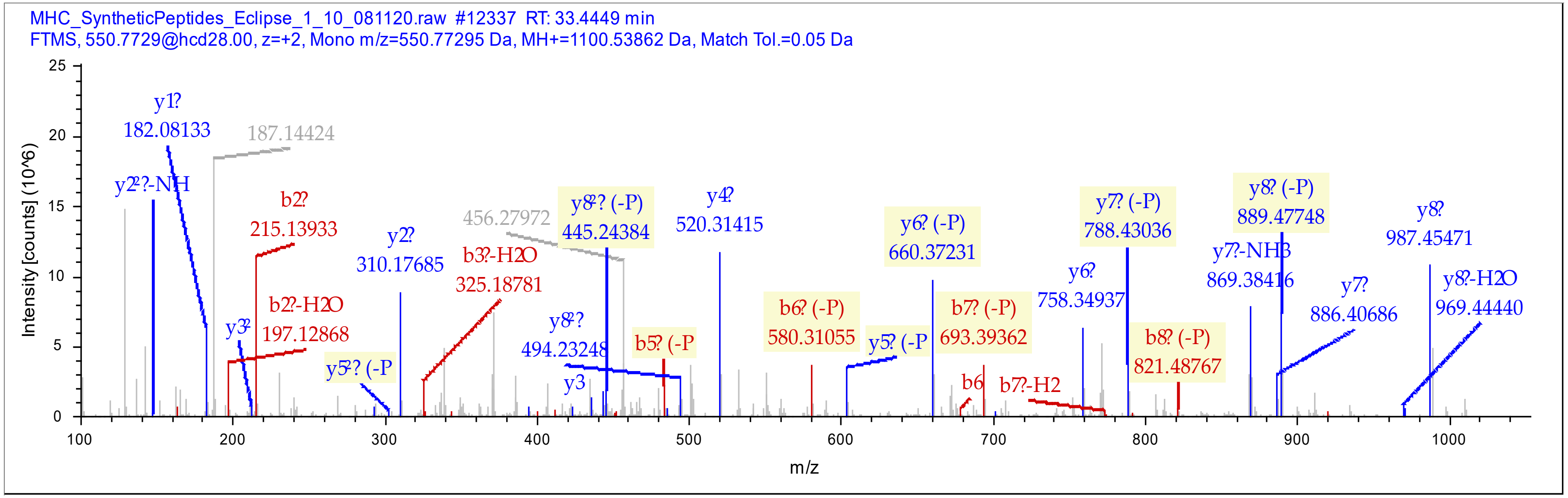

Experimental

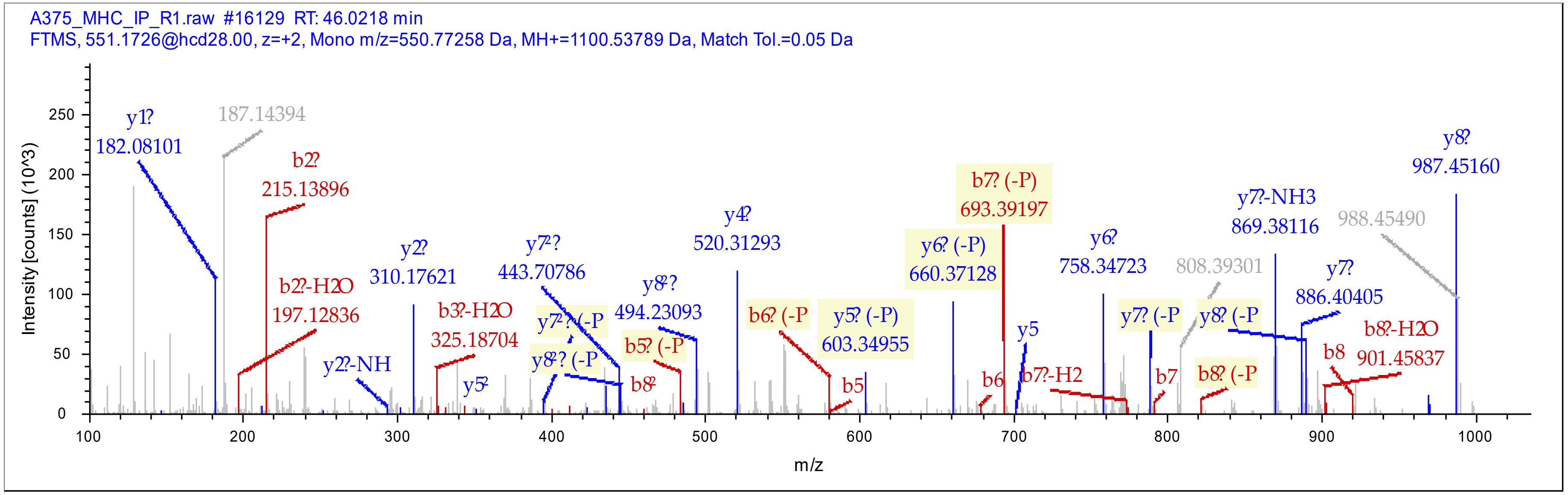

KAFSPVRSV – Phospho

Synthetic

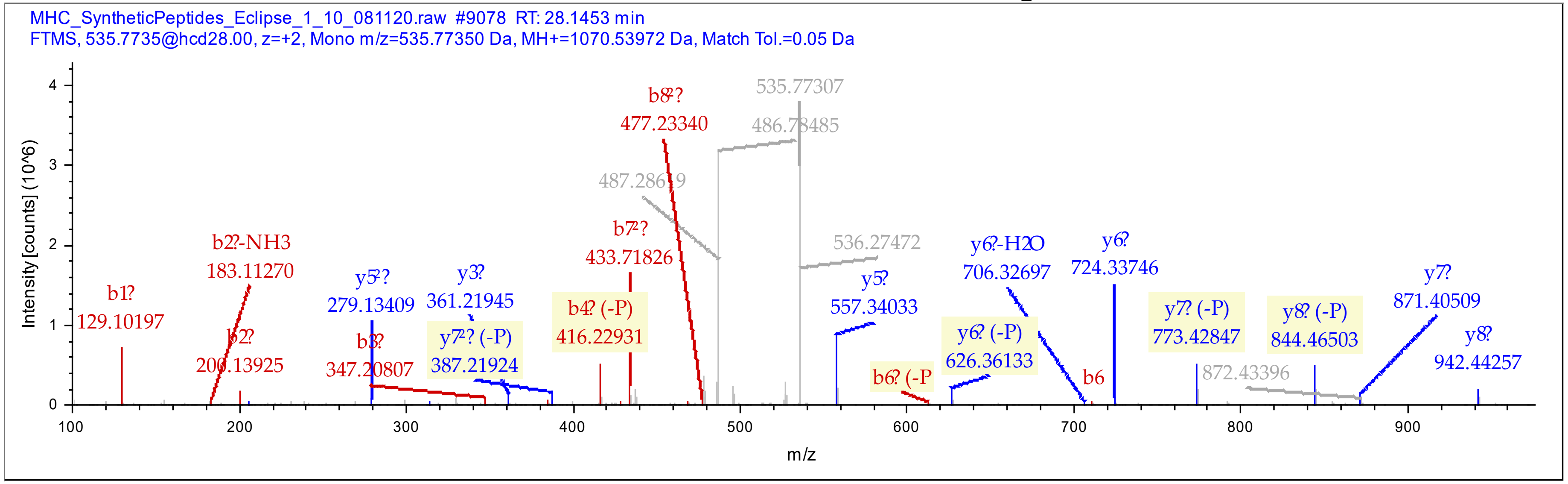

Experimental

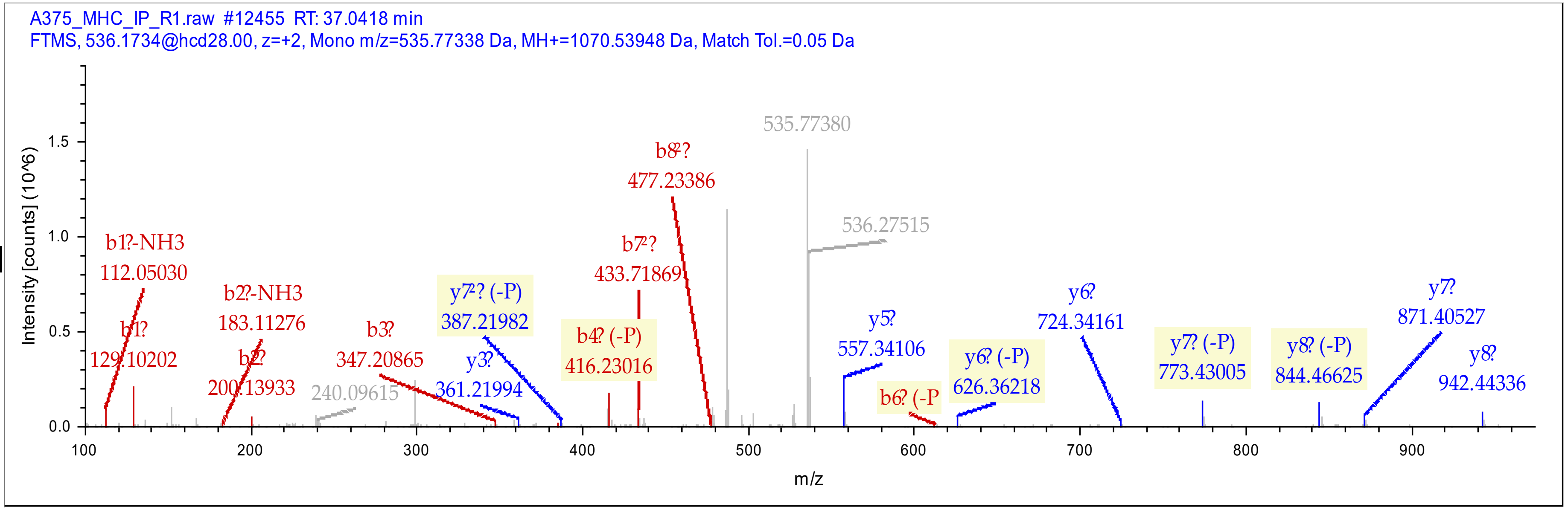

KAITPPQQPY – Phospho

Synthetic

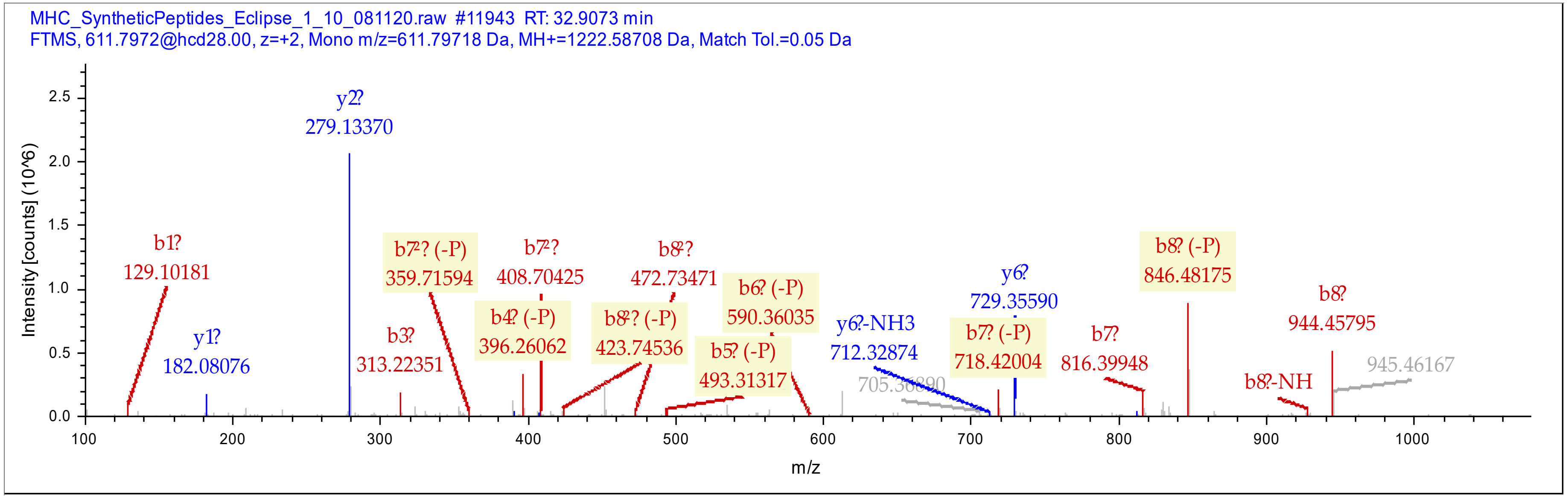

Experimental

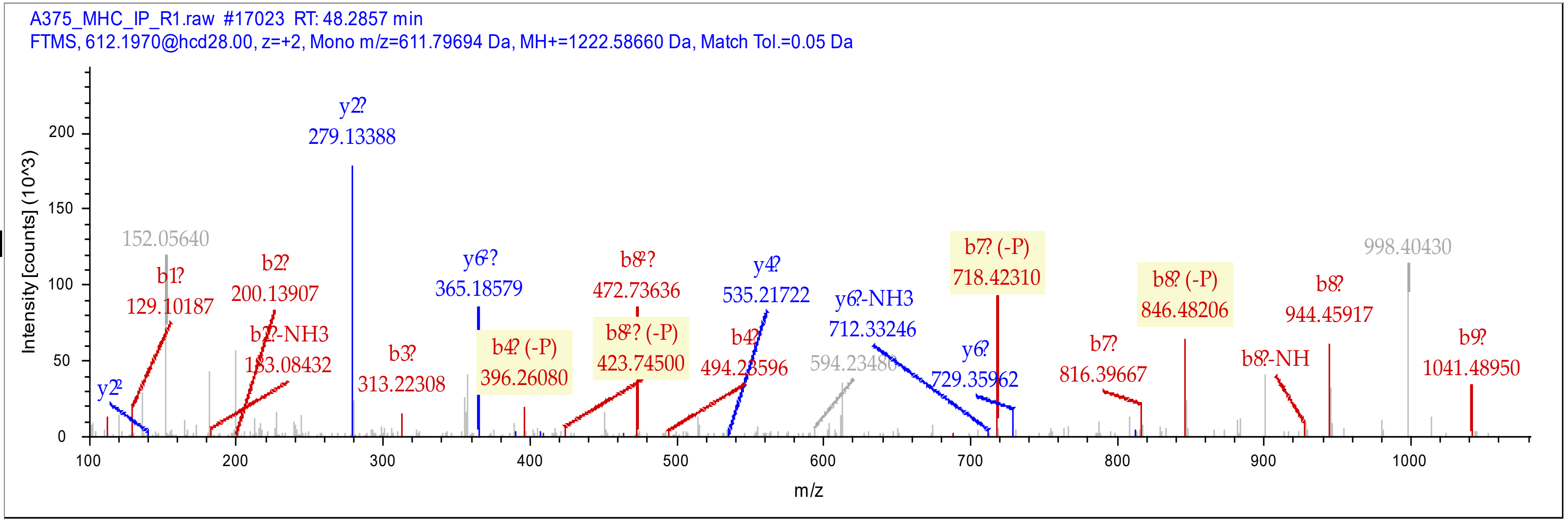

KLFSPSKEAEL – Phospho

Synthetic

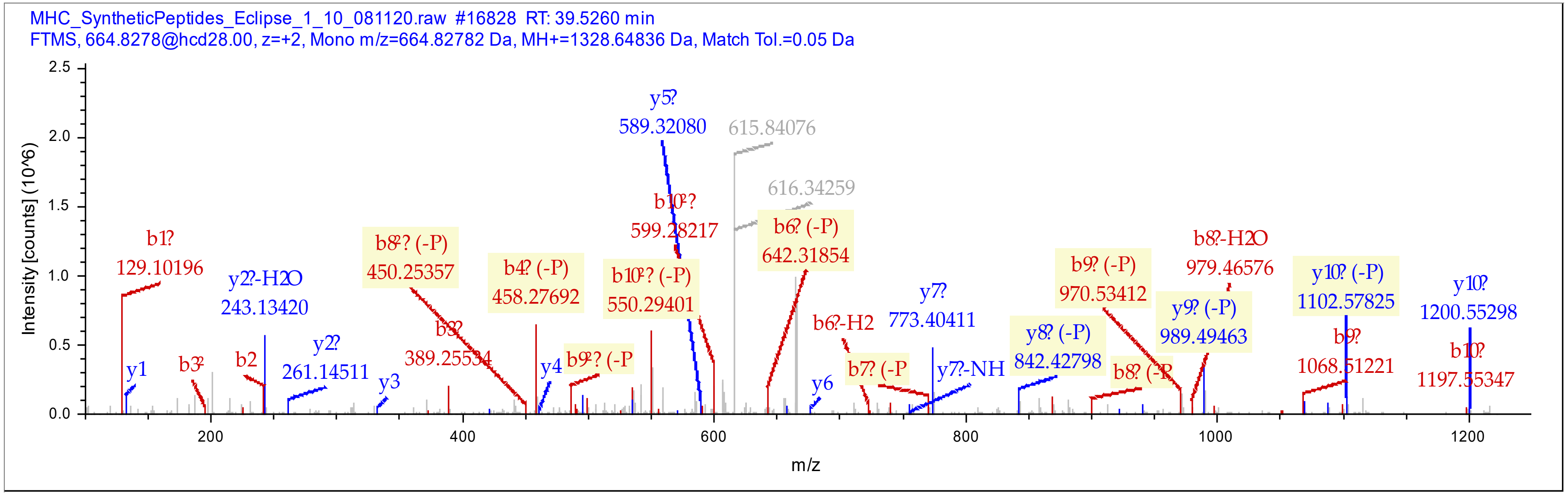

Experimental

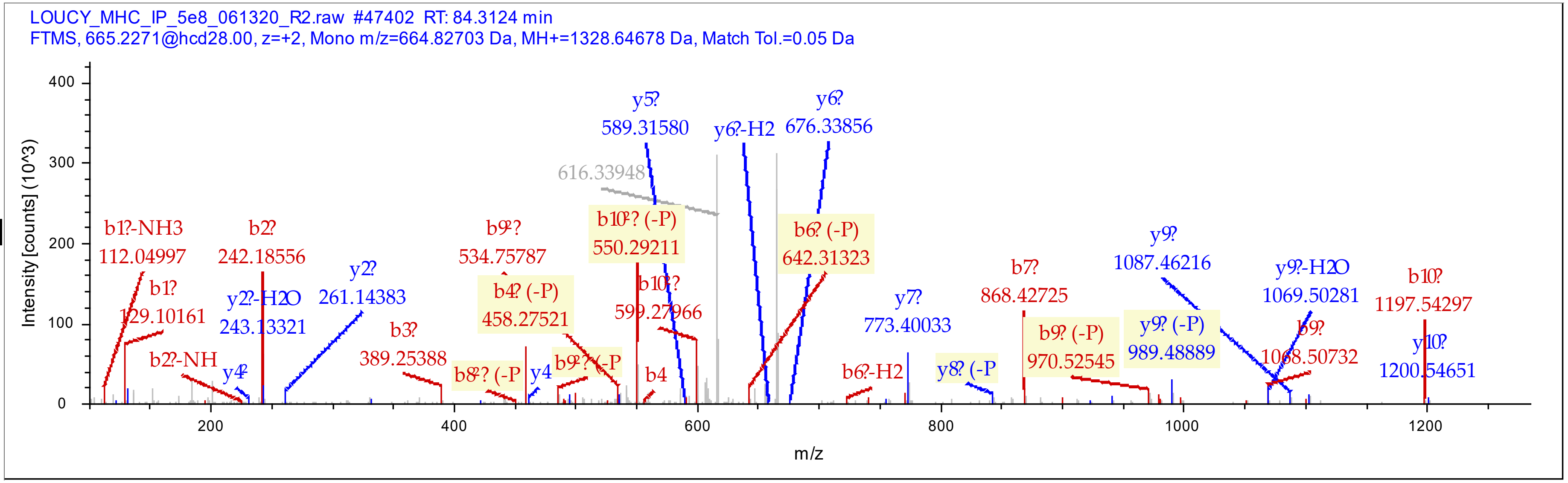

LPASPAHQL – Phospho

Synthetic

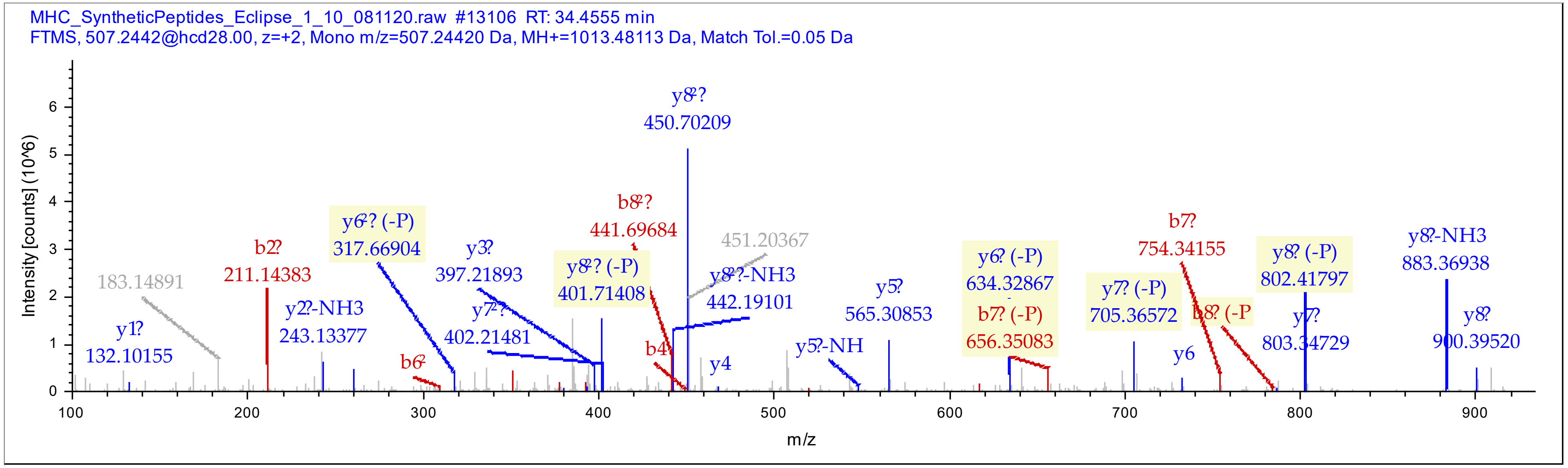

Experimental

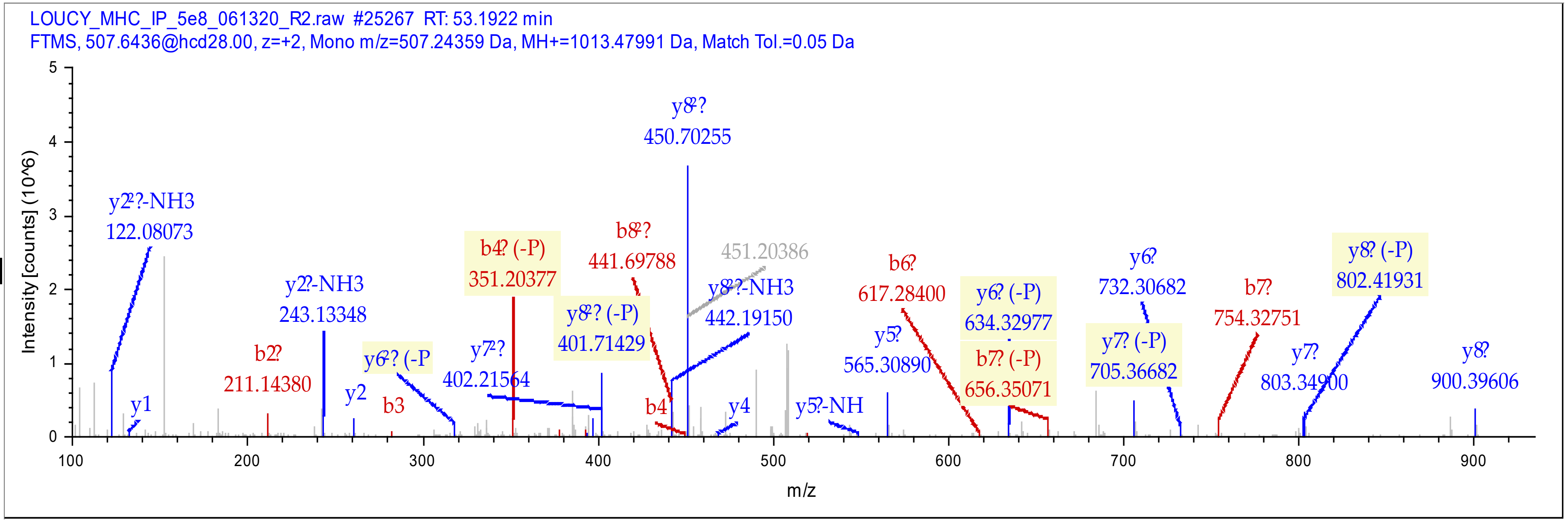

KRFSGTVRL – Phospho

Synthetic

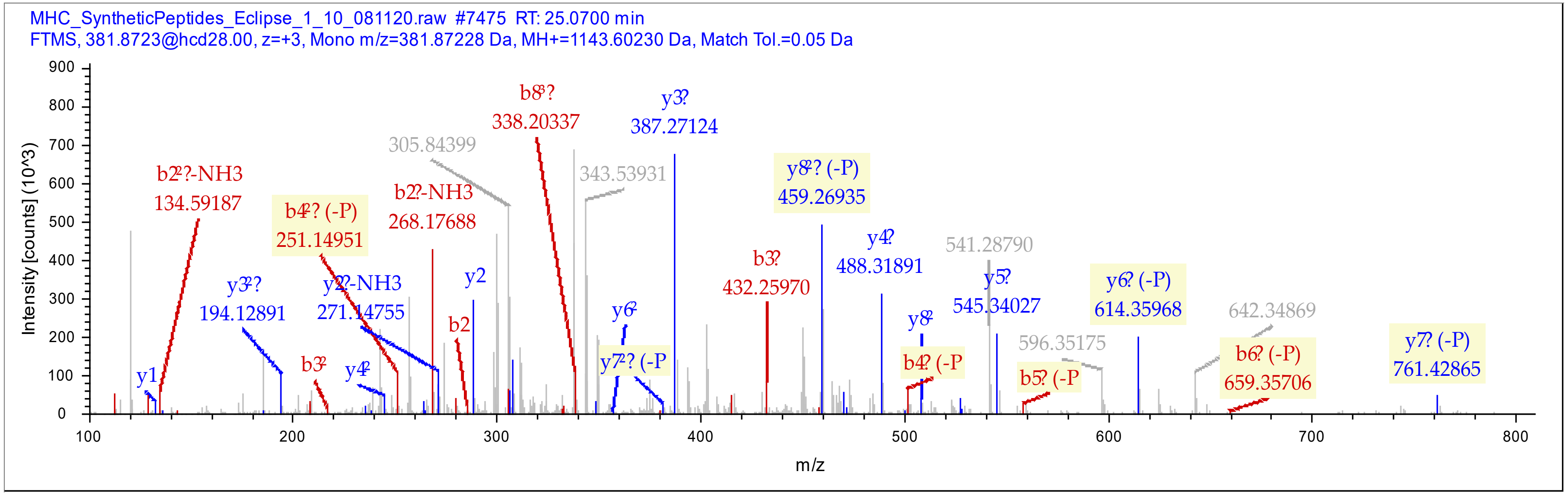

Experimental

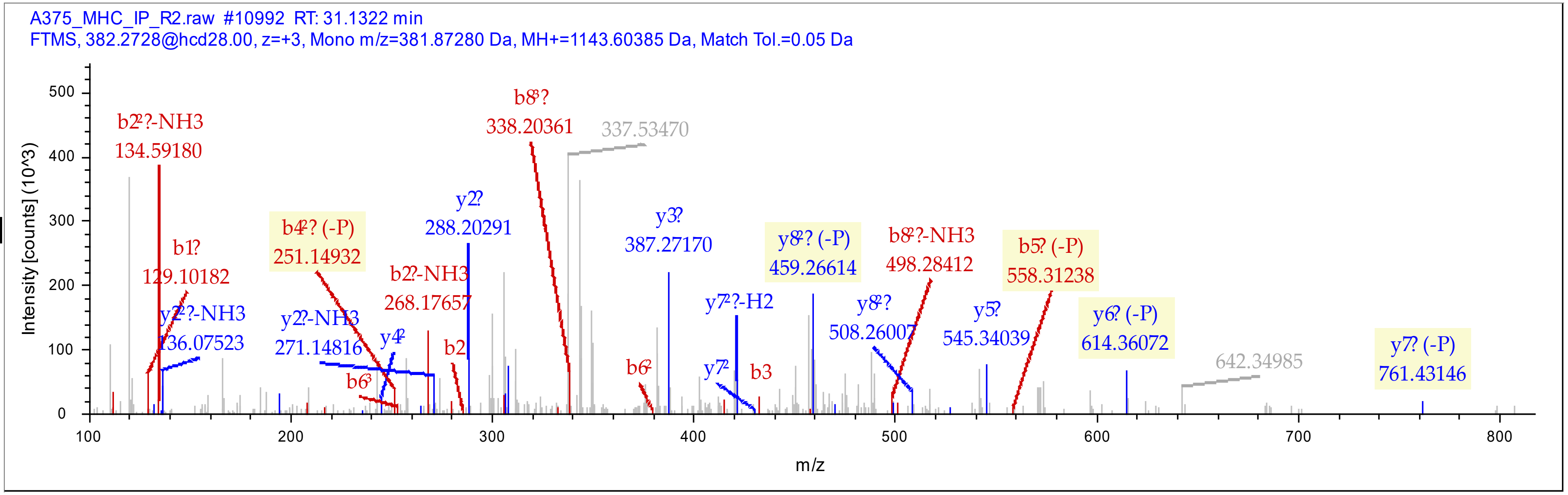

NTDSPLRY – Phospho

Synthetic

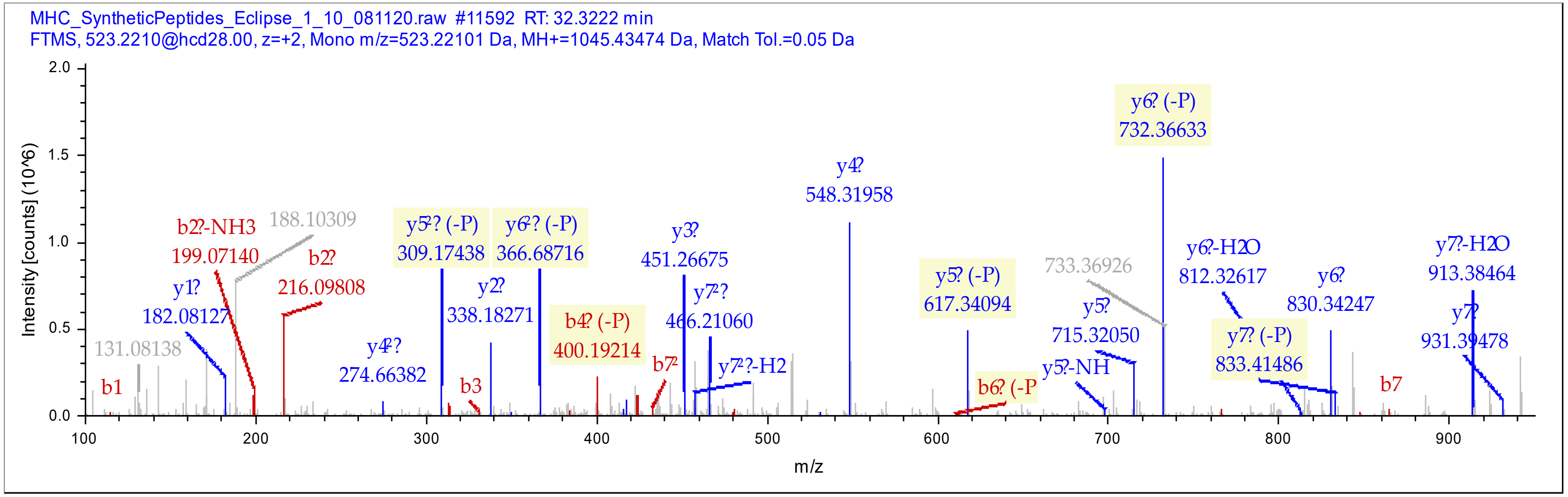

Experimental

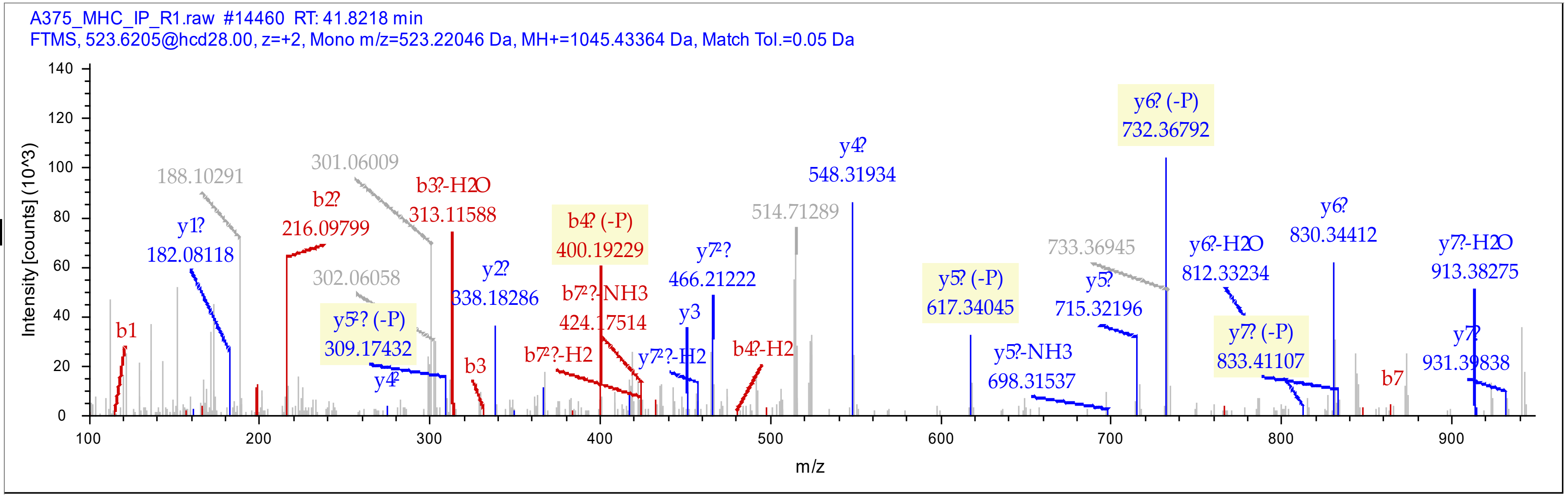

RPYSPPFFSL – Phospho

Synthetic

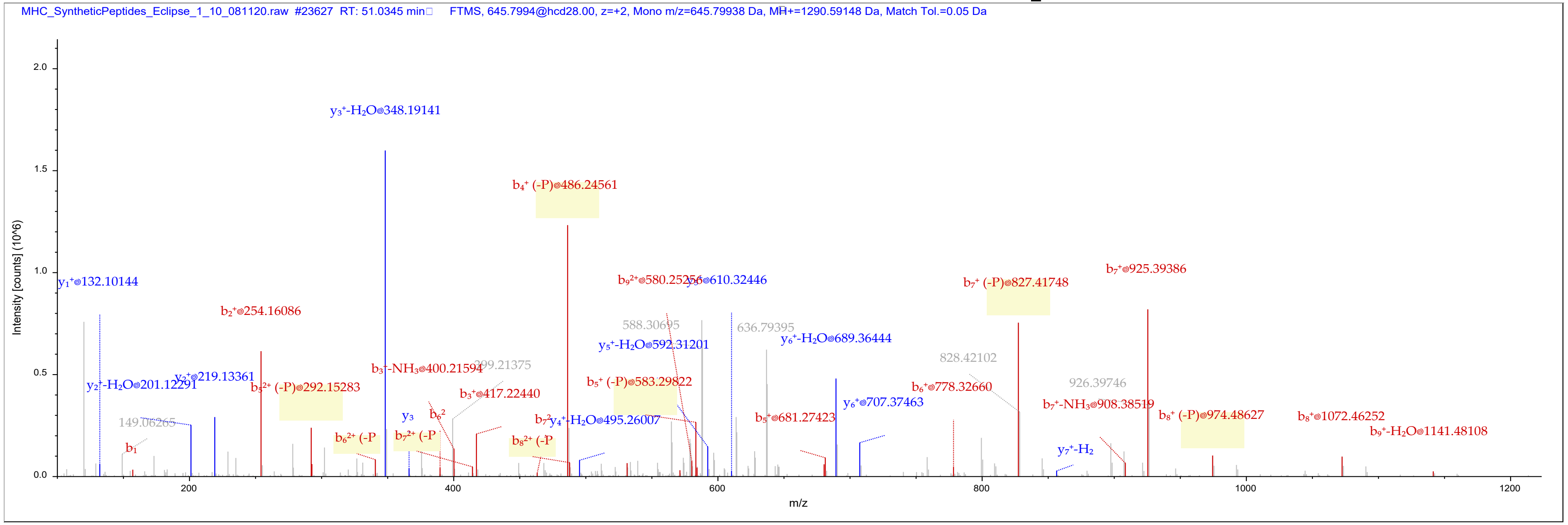

Experimental

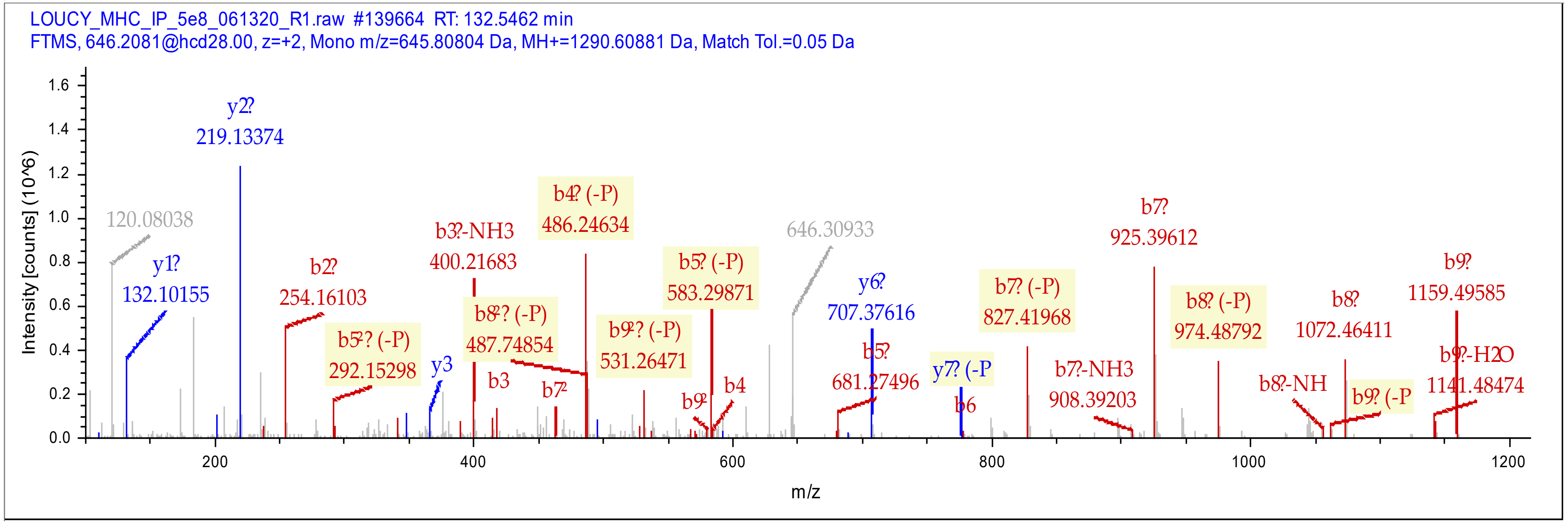

RLFSKELRC – Phospho

Synthetic

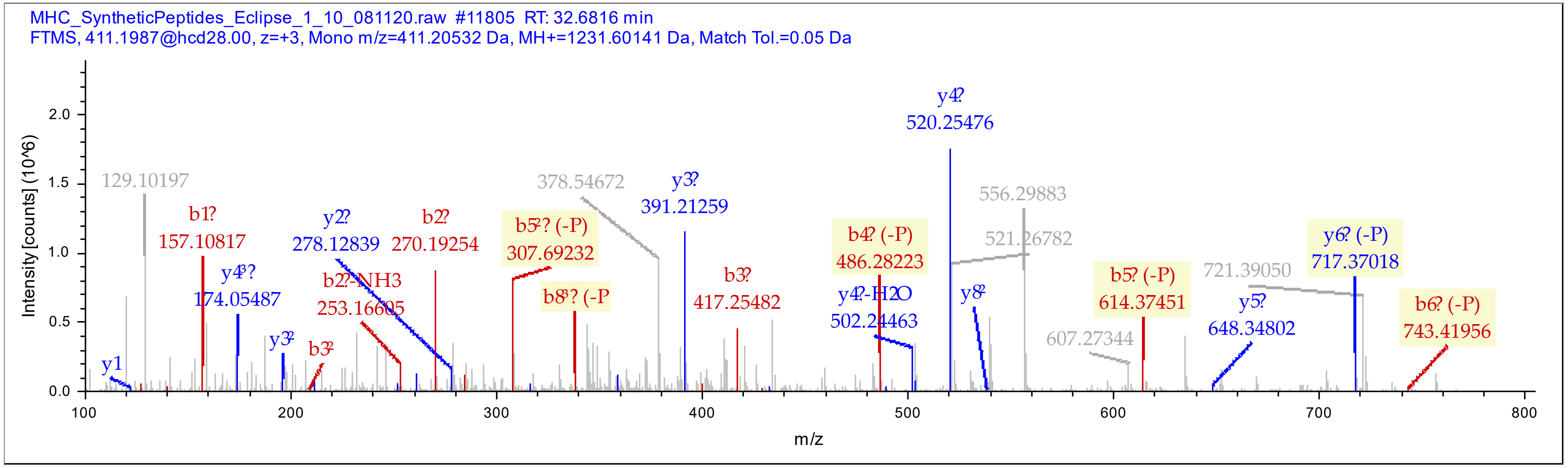

Experimental

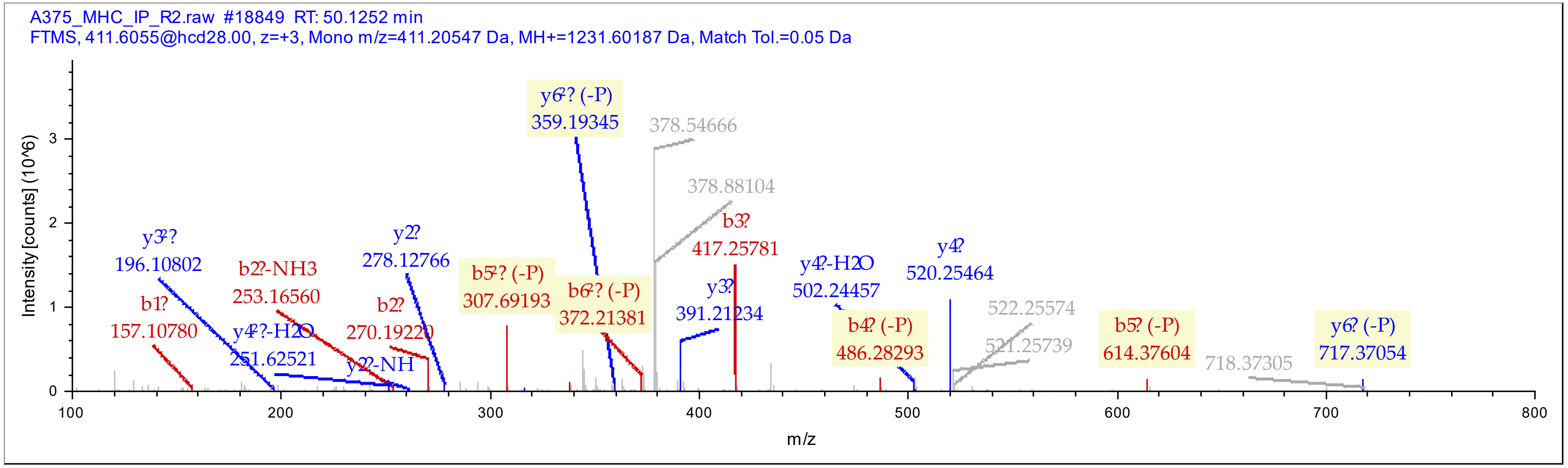

RQISQDVKL – Phospho

Synthetic

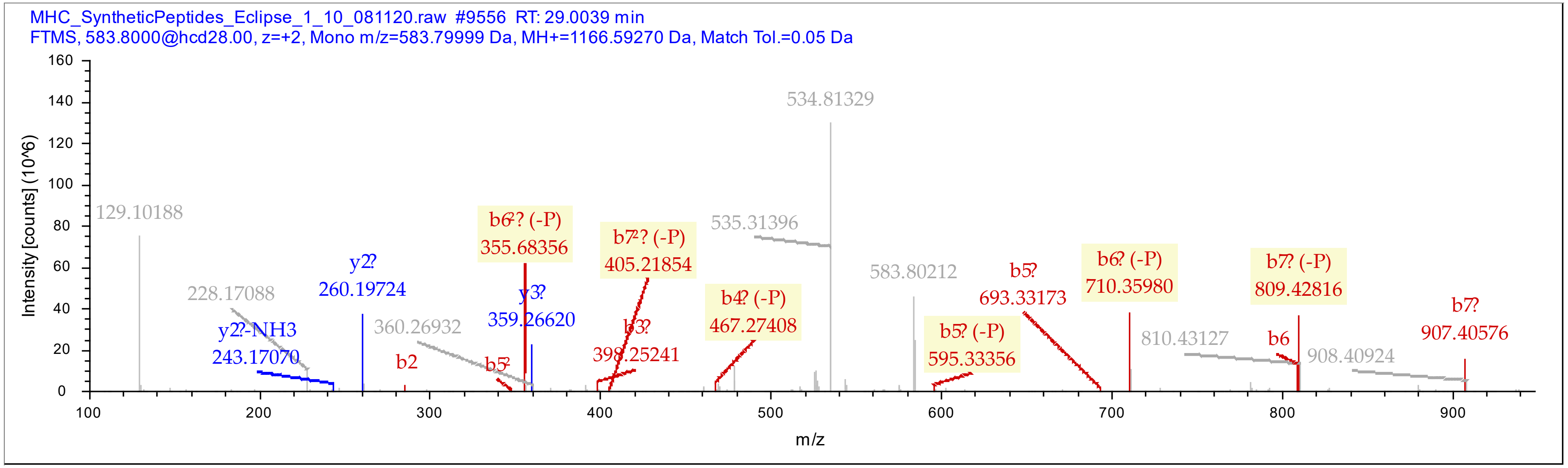

Experimental

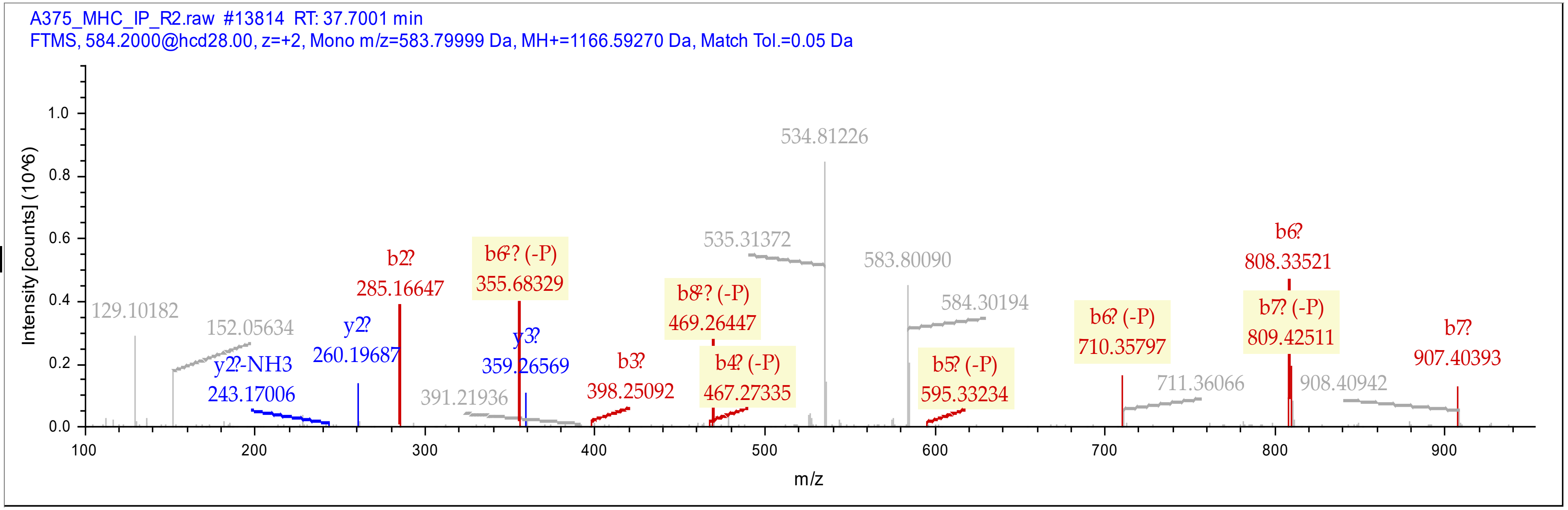

SDVSLTACKV – Phospho

Synthetic

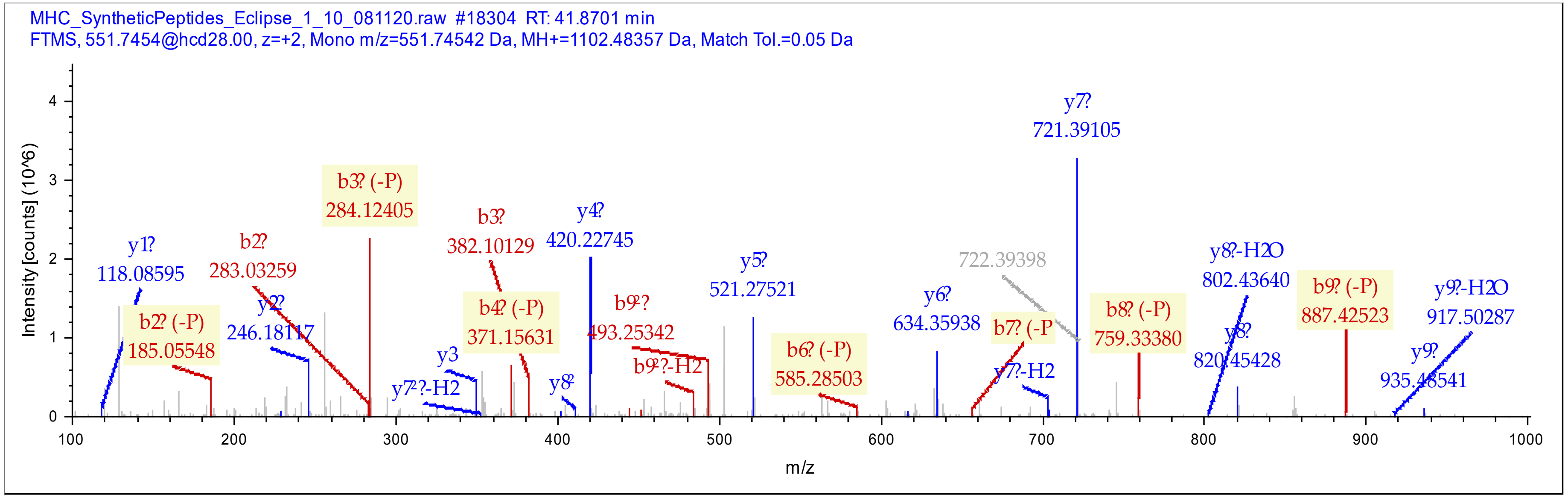

Experimental

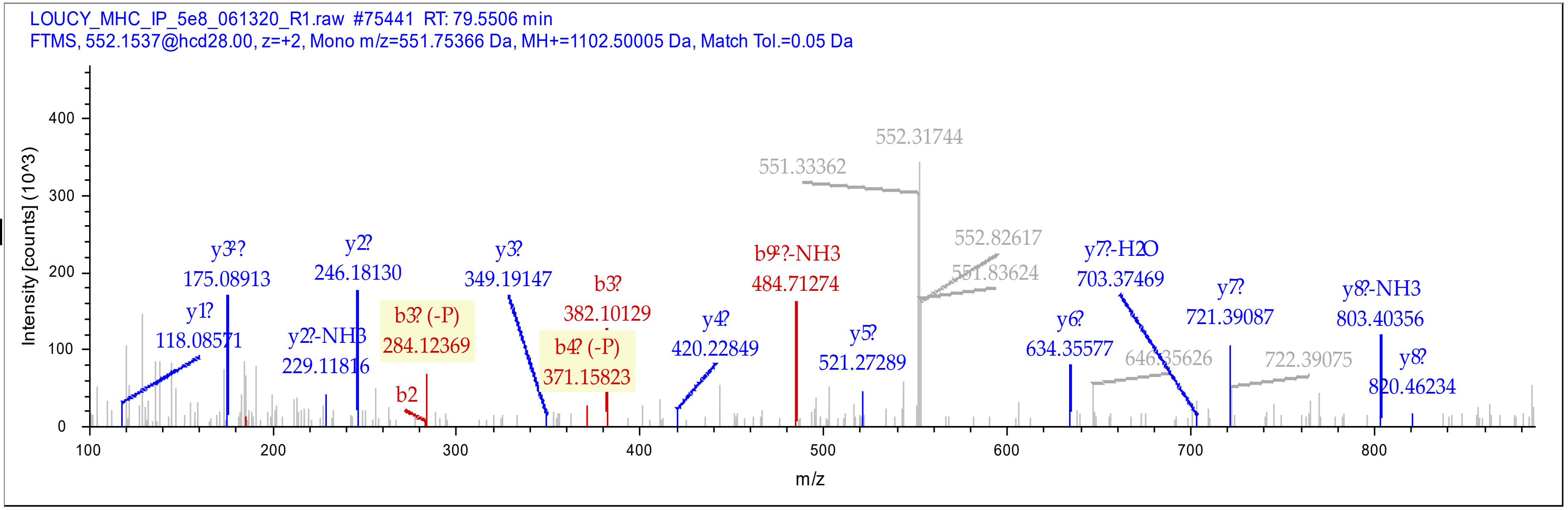

SEASPSREAI – Phospho

Synthetic

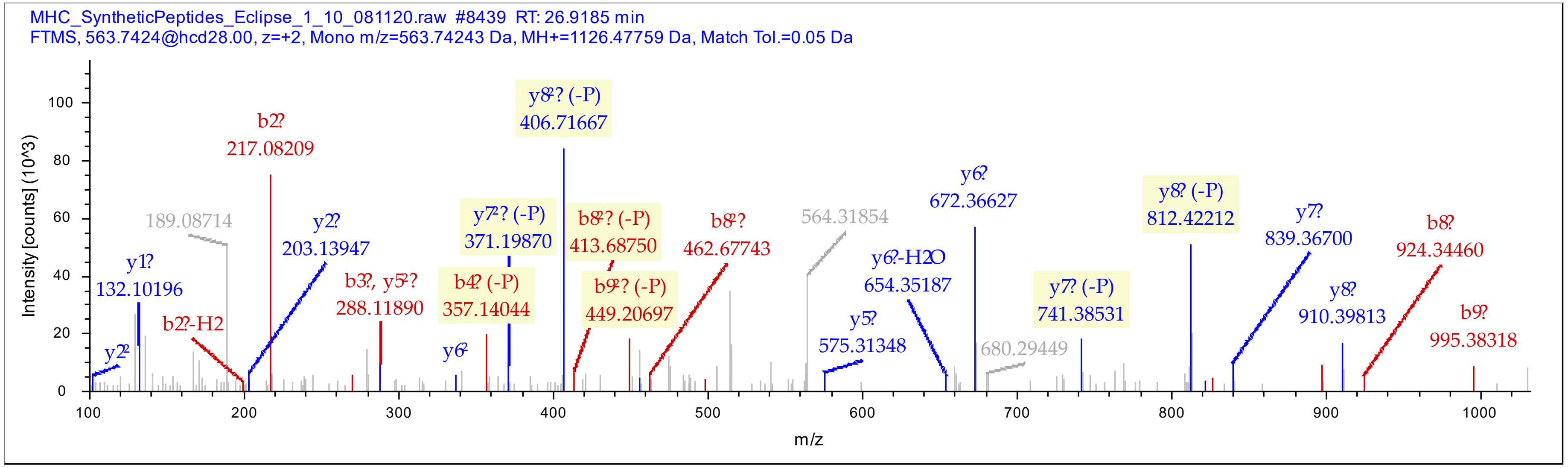

Experimental

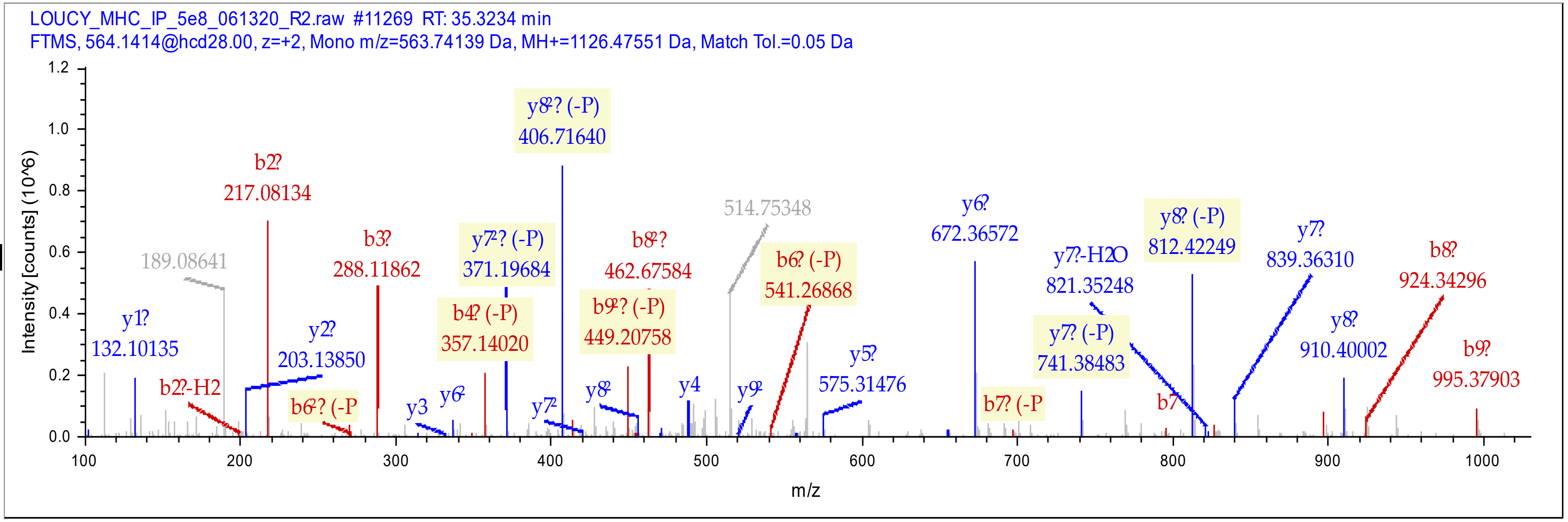

SEGSLGSHF – Phospho

Synthetic

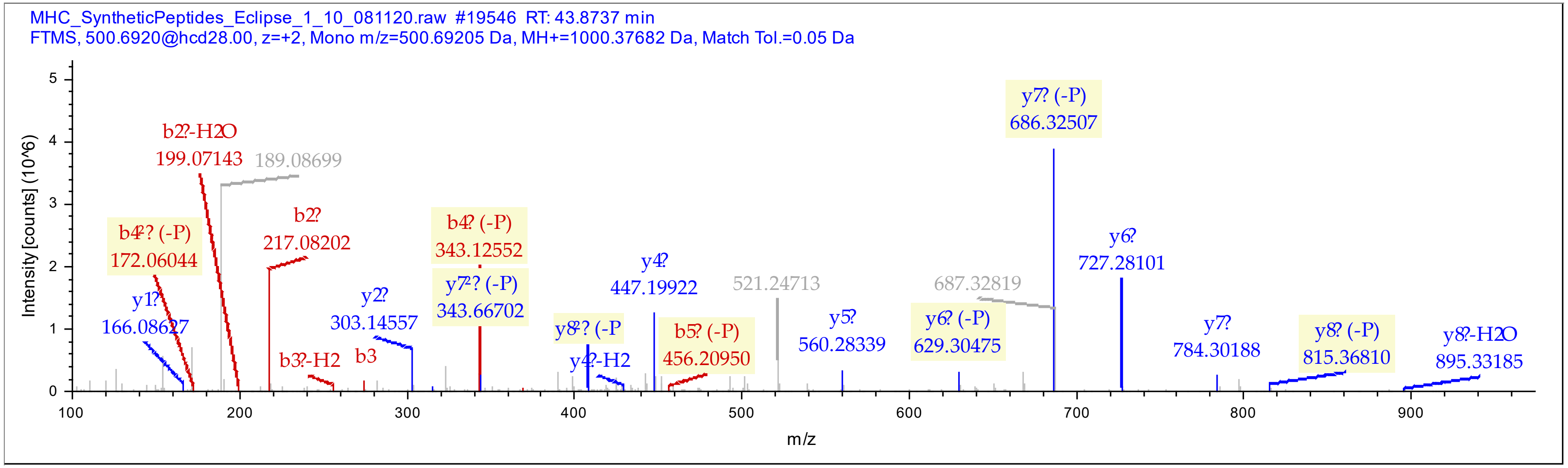

Experimental

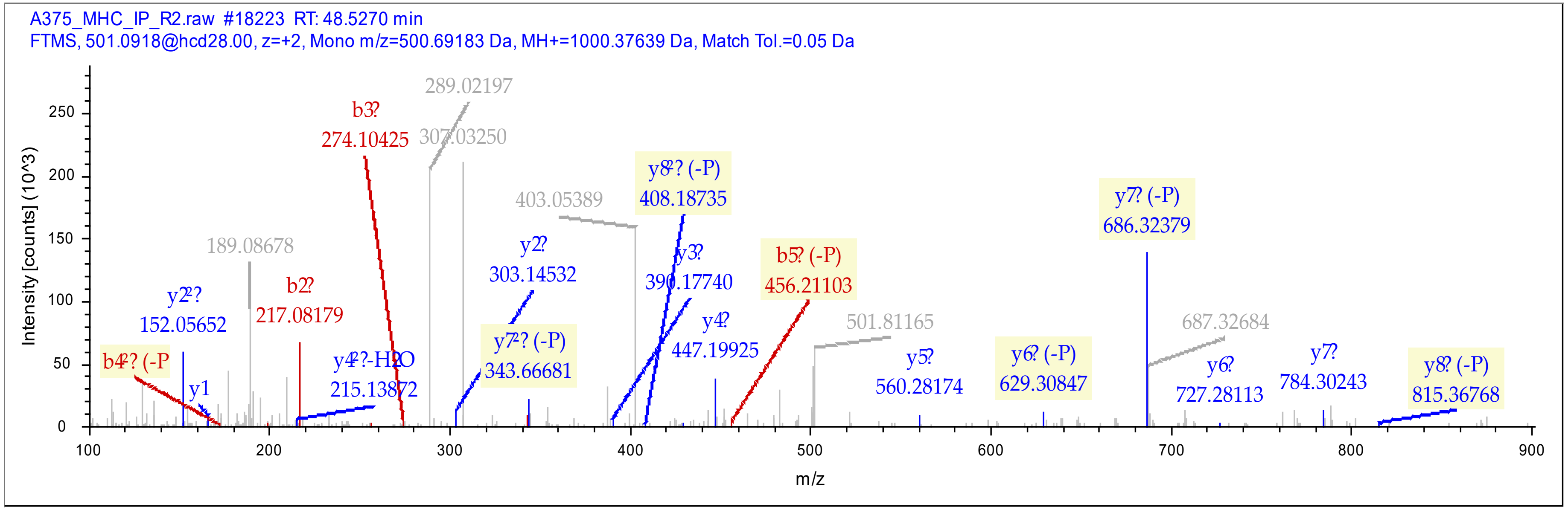

SFFSRQLSL – Phospho

Synthetic

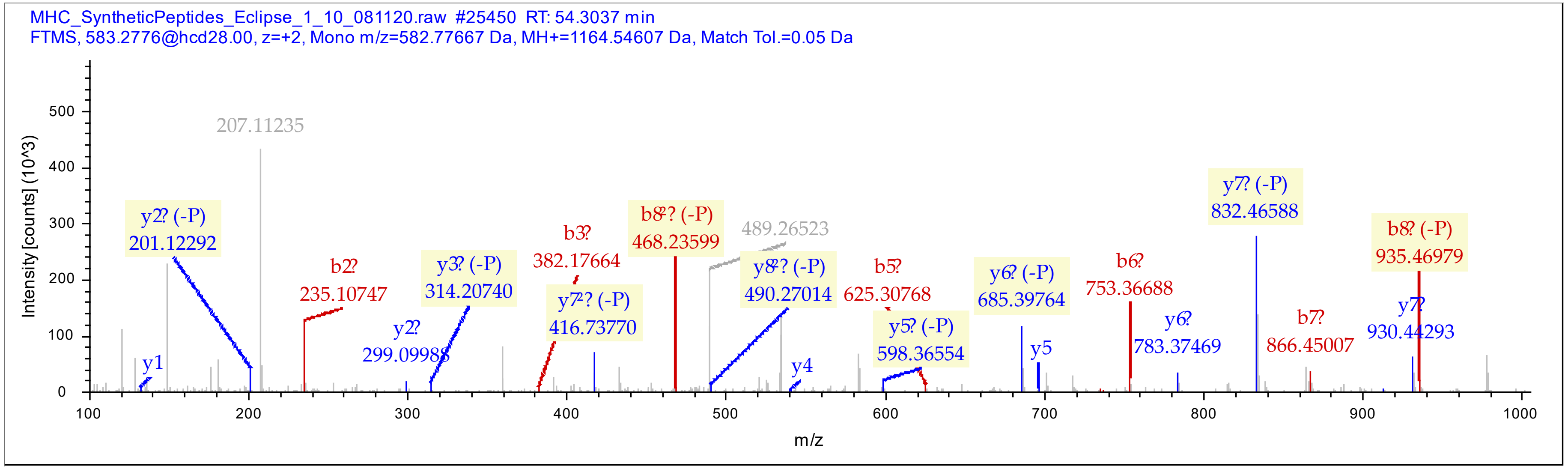

Experimental

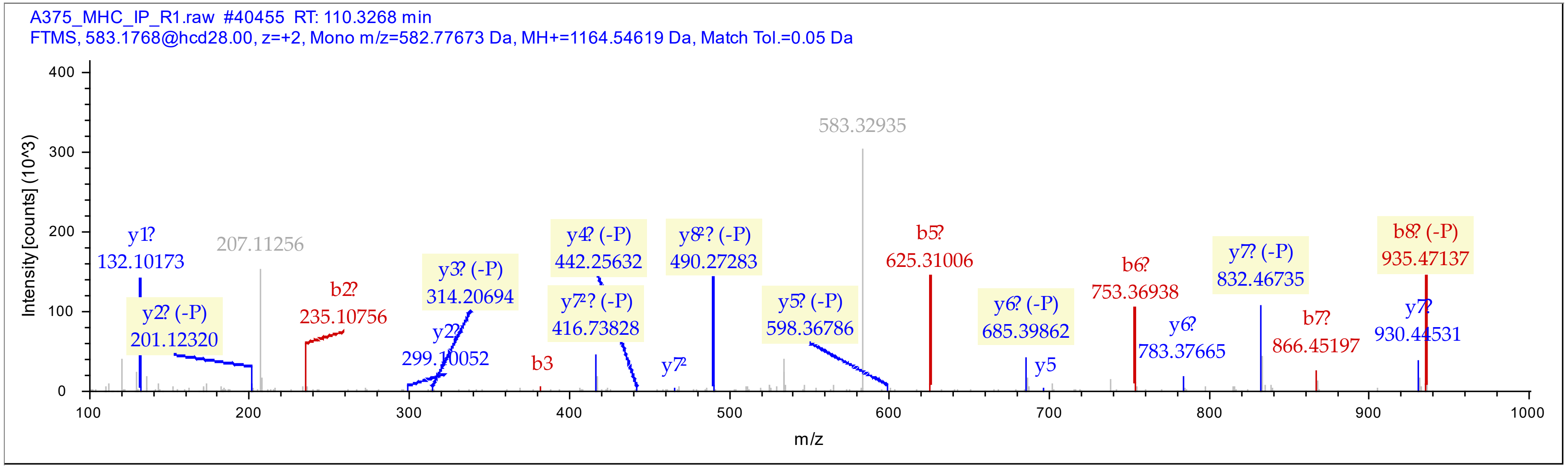

SPRTPVSPVKF – Phospho

Synthetic

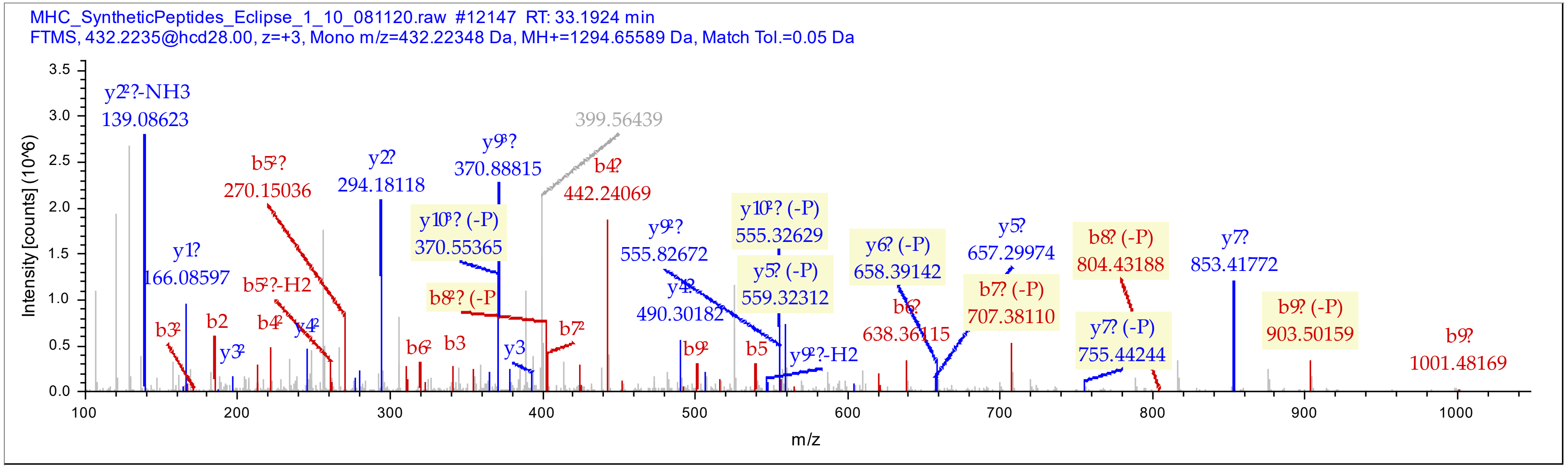

Experimental

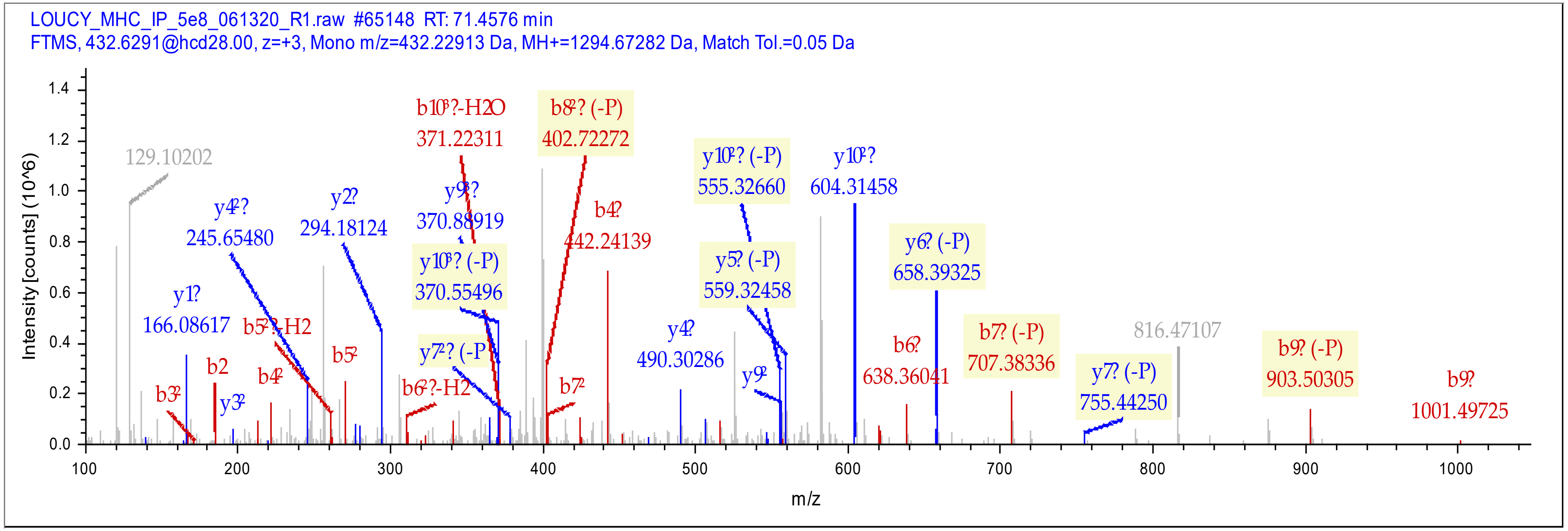

VLASPLKTGR – Phospho

Synthetic

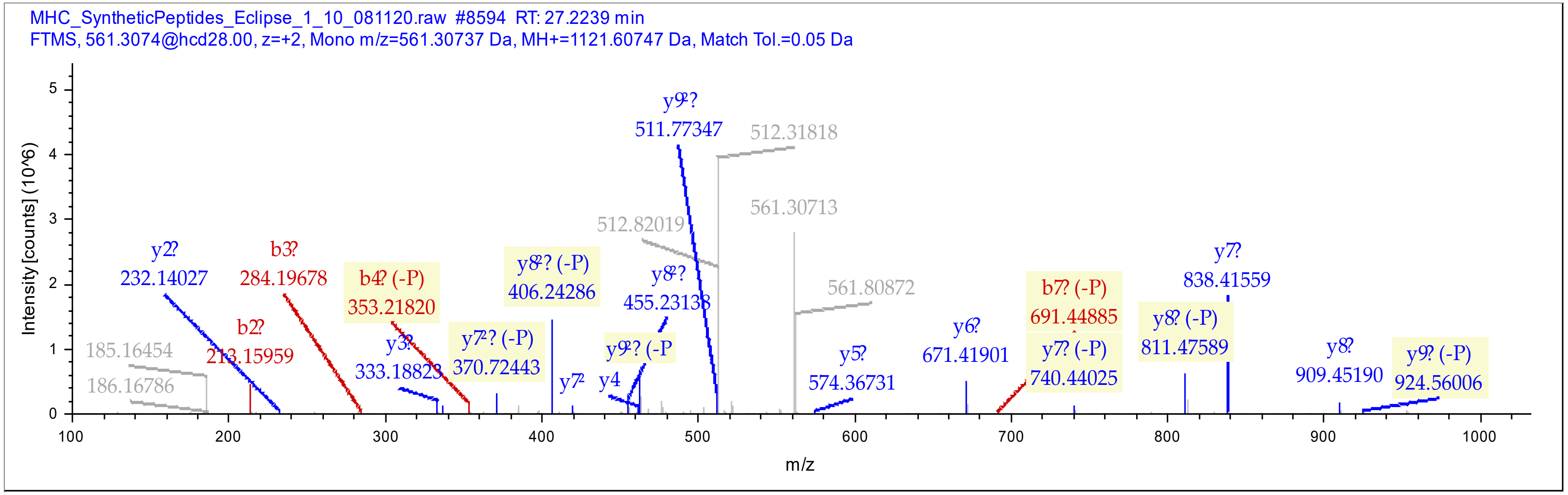

Experimental

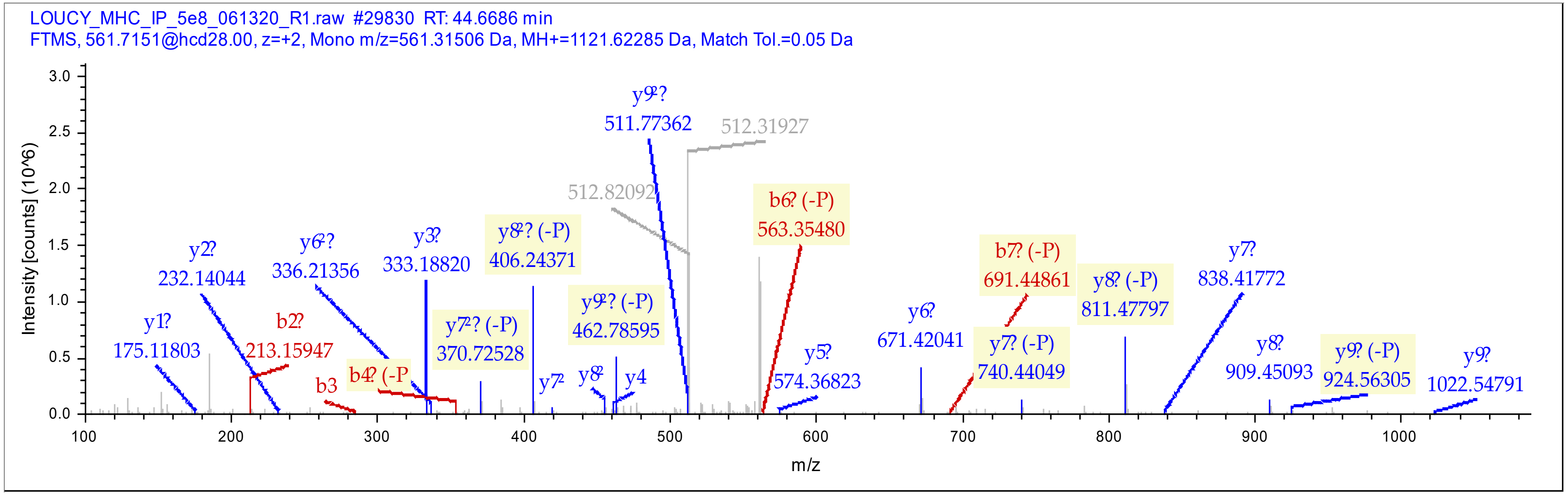

ATAGPRLGW – Phosphorylation

Synthetic

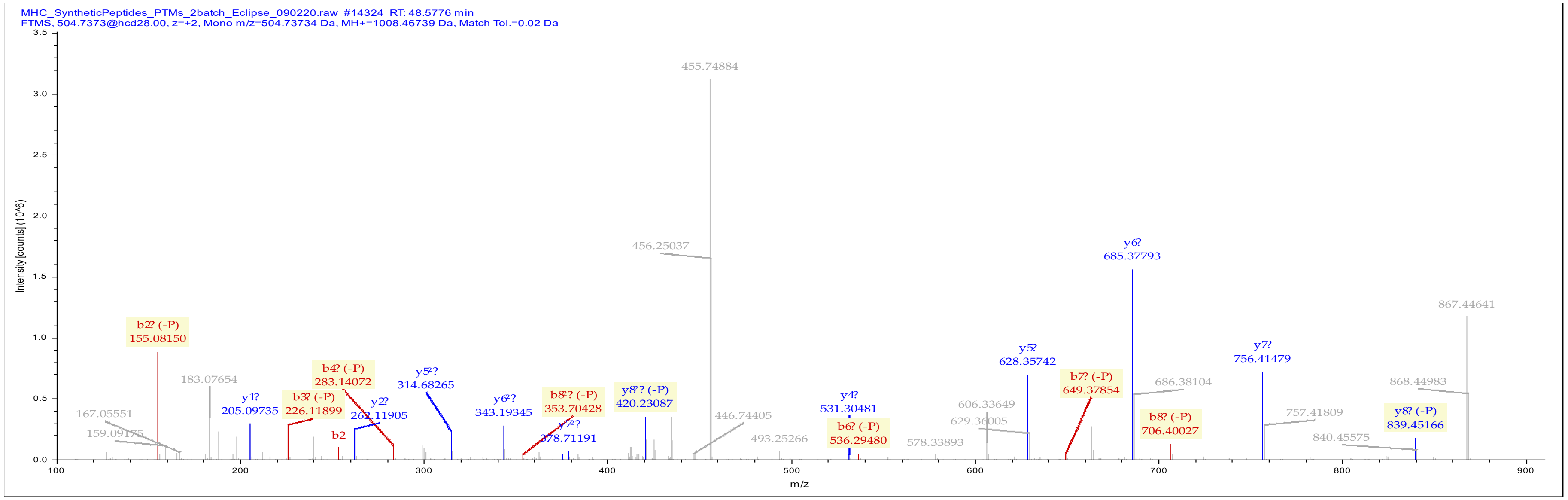

Experimental

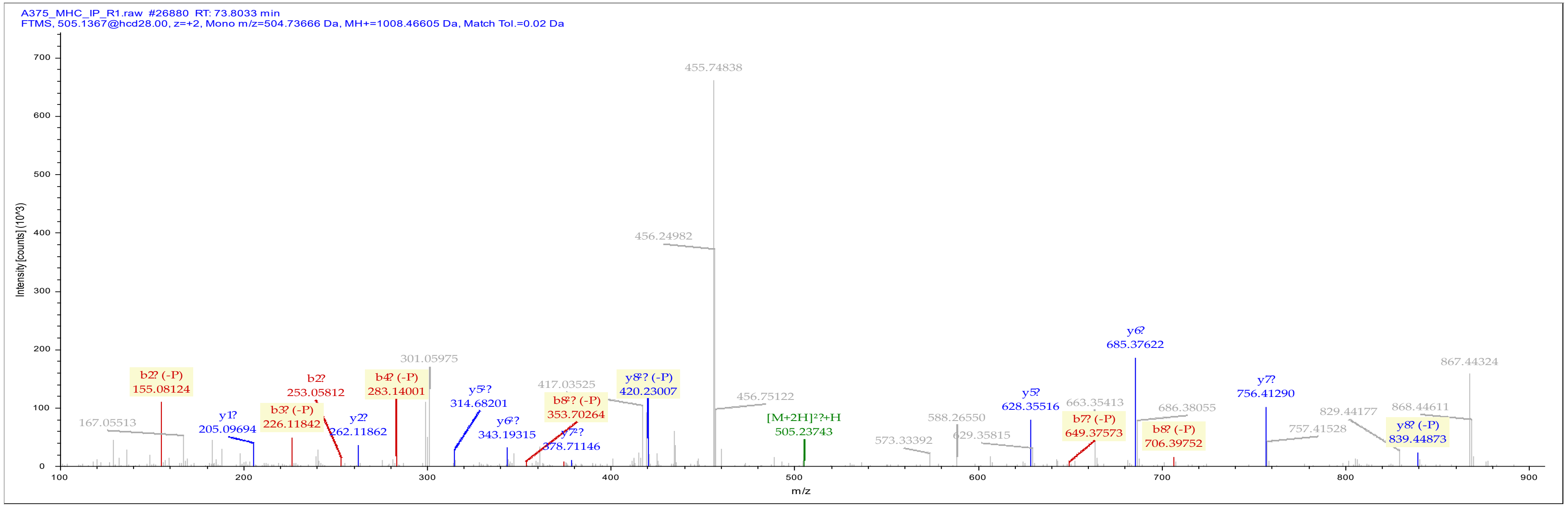

RSASPSSQGW – Phosphorylation

Synthetic

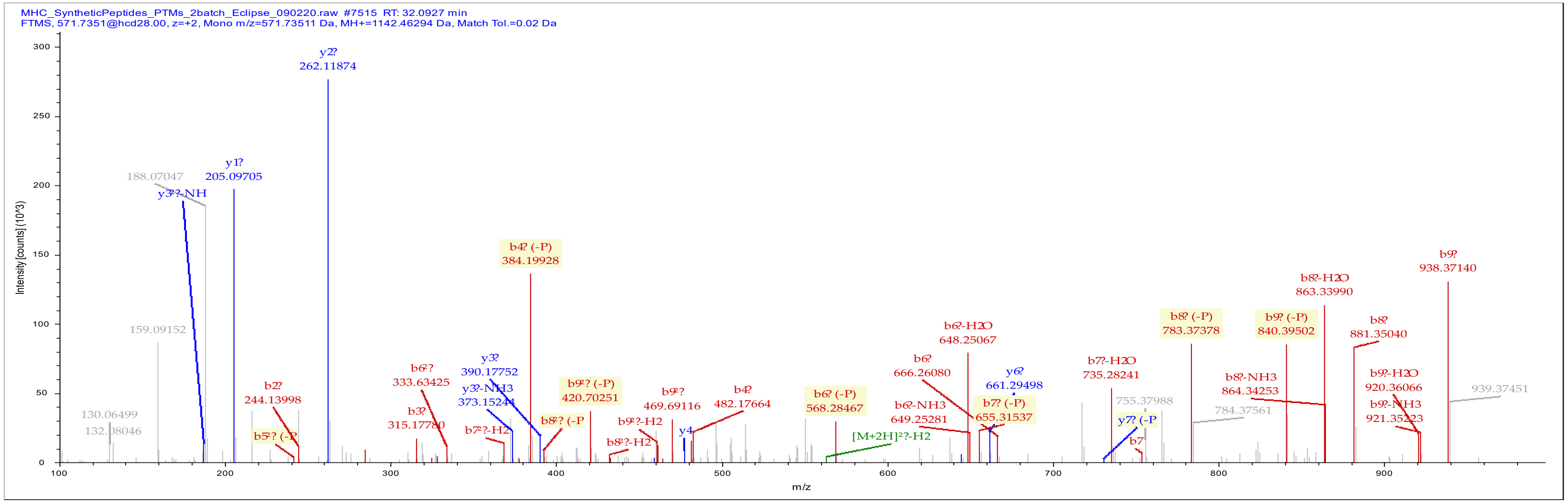

Experimental

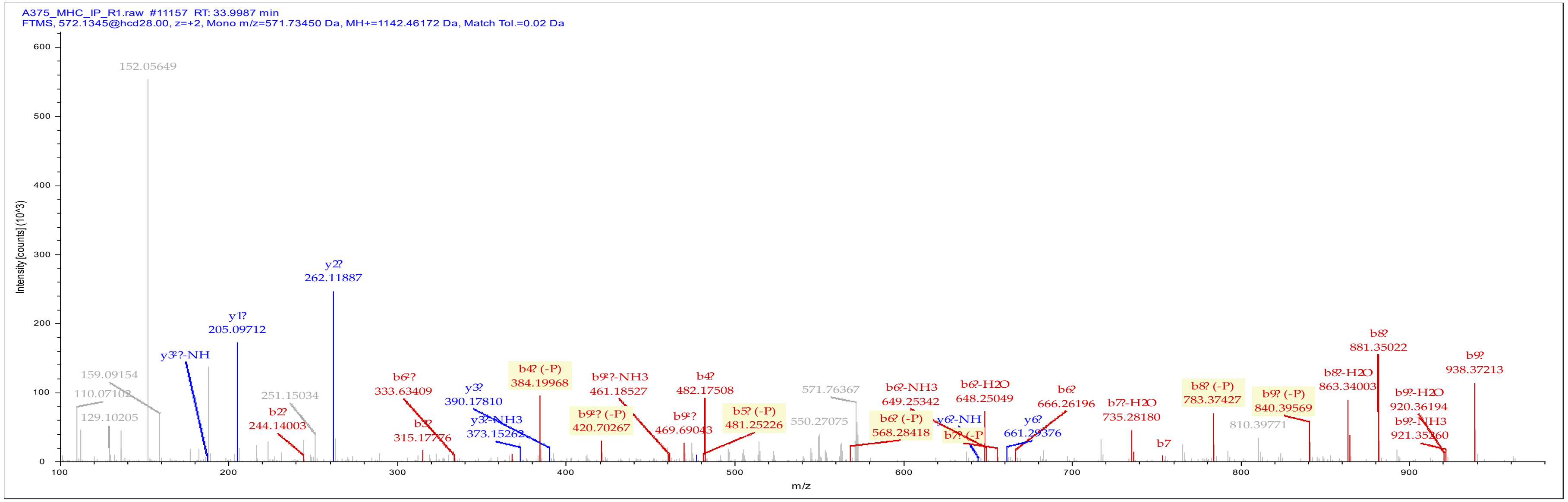

RVASPTSGV – Phosphorylation

Synthetic

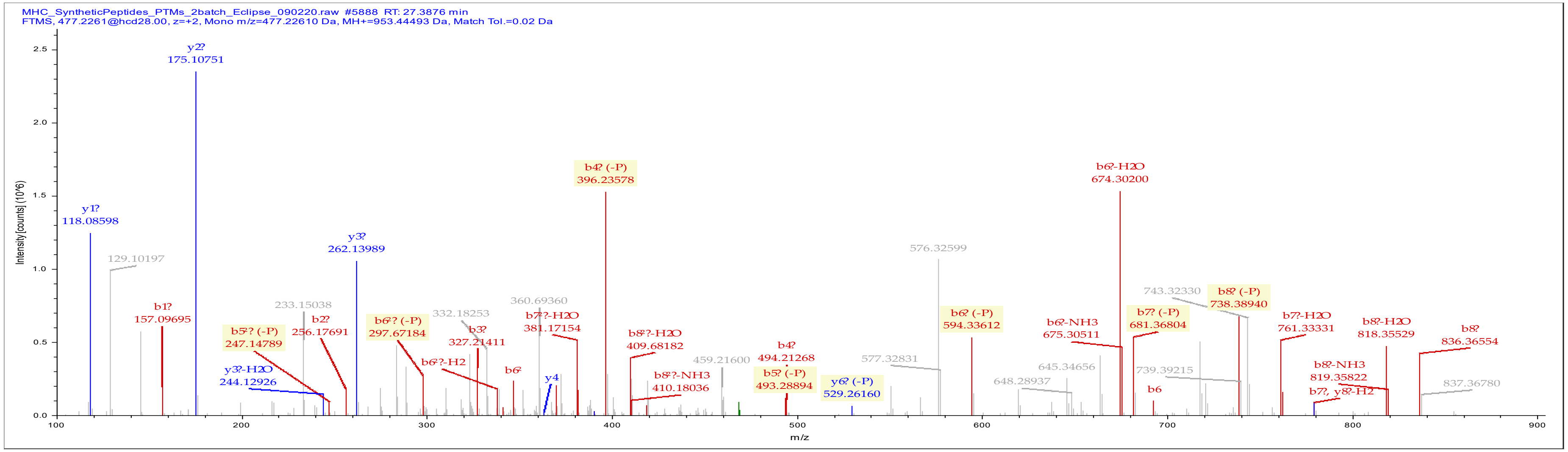

Experimental

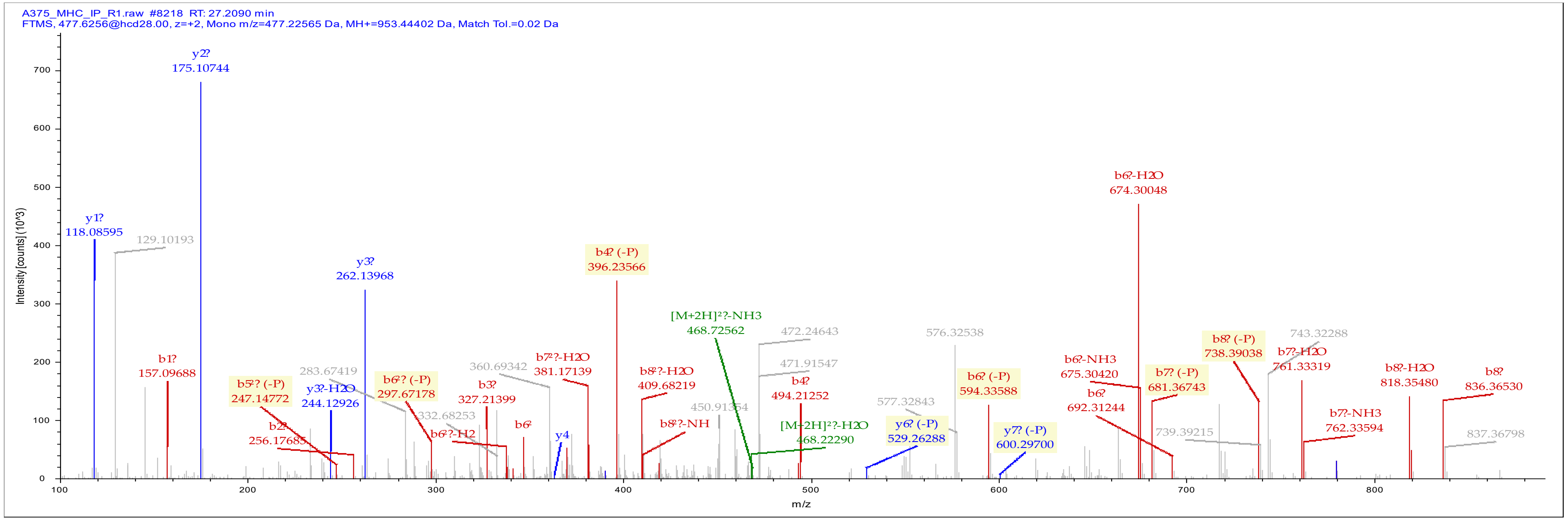

SSDPASQLSY – Phosphorylation

Synthetic

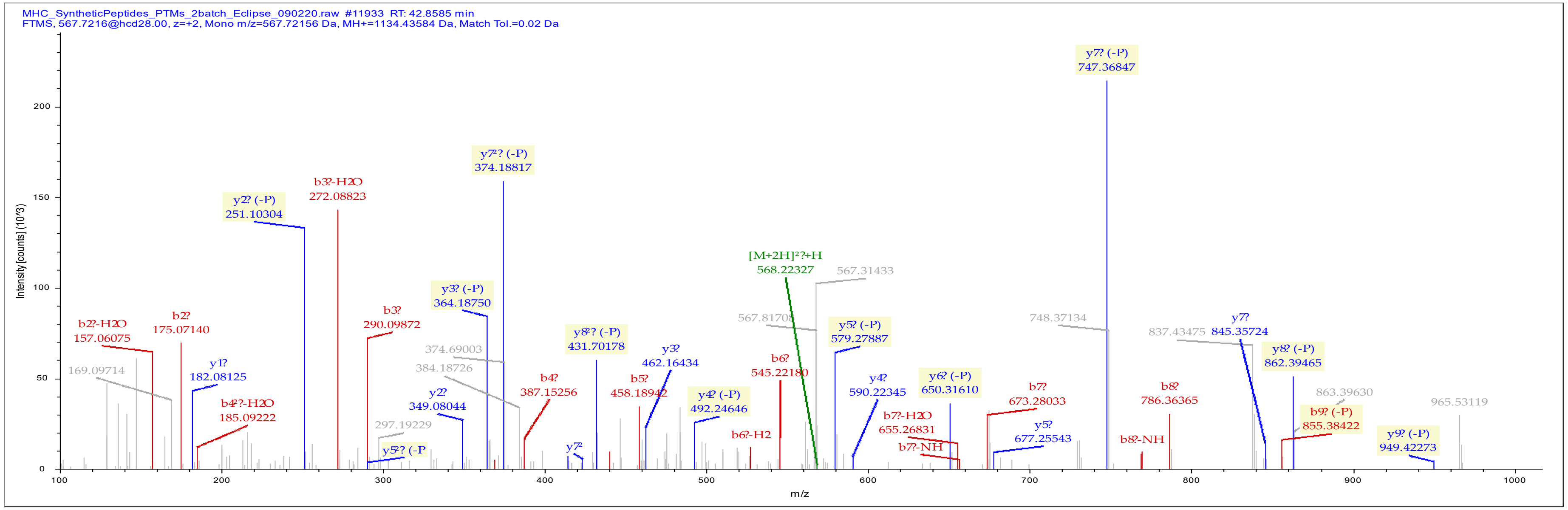

Experimental

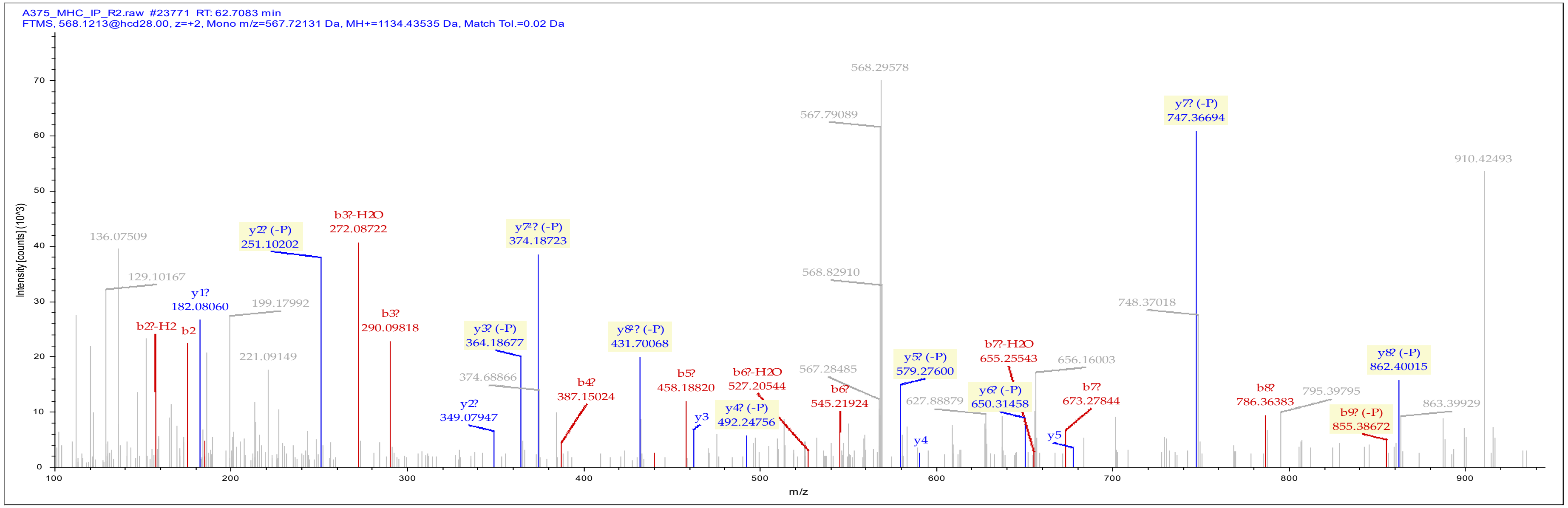

Spliced peptide - AAAAGARLY

Synthetic

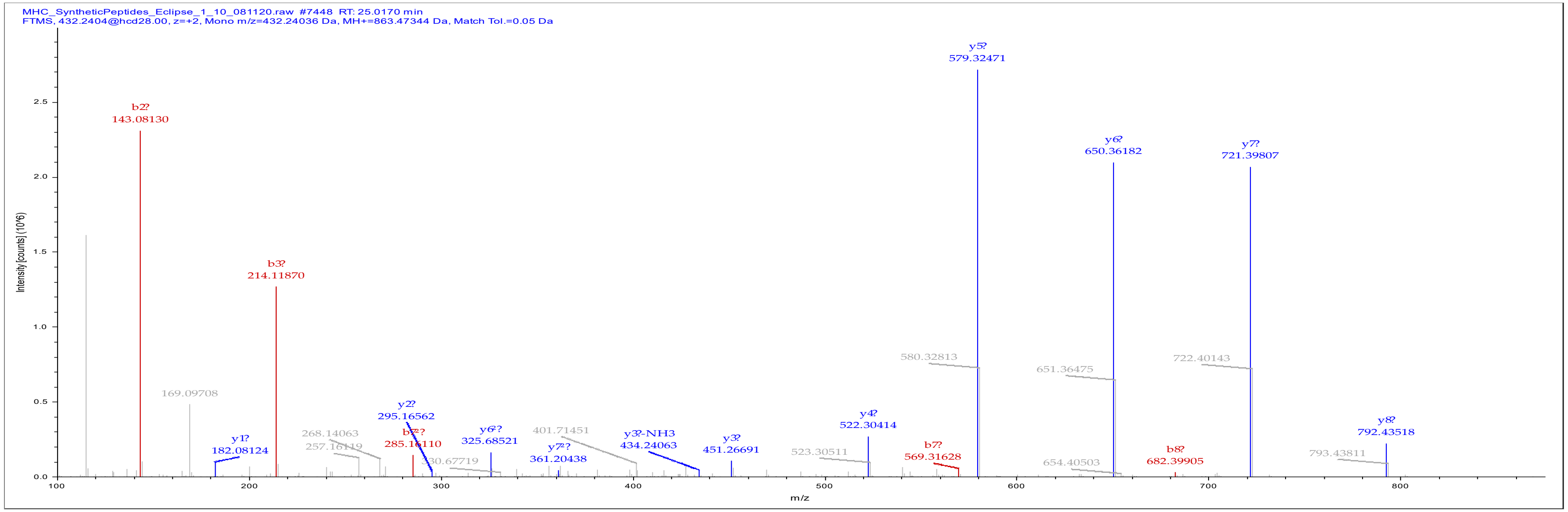

Experimental

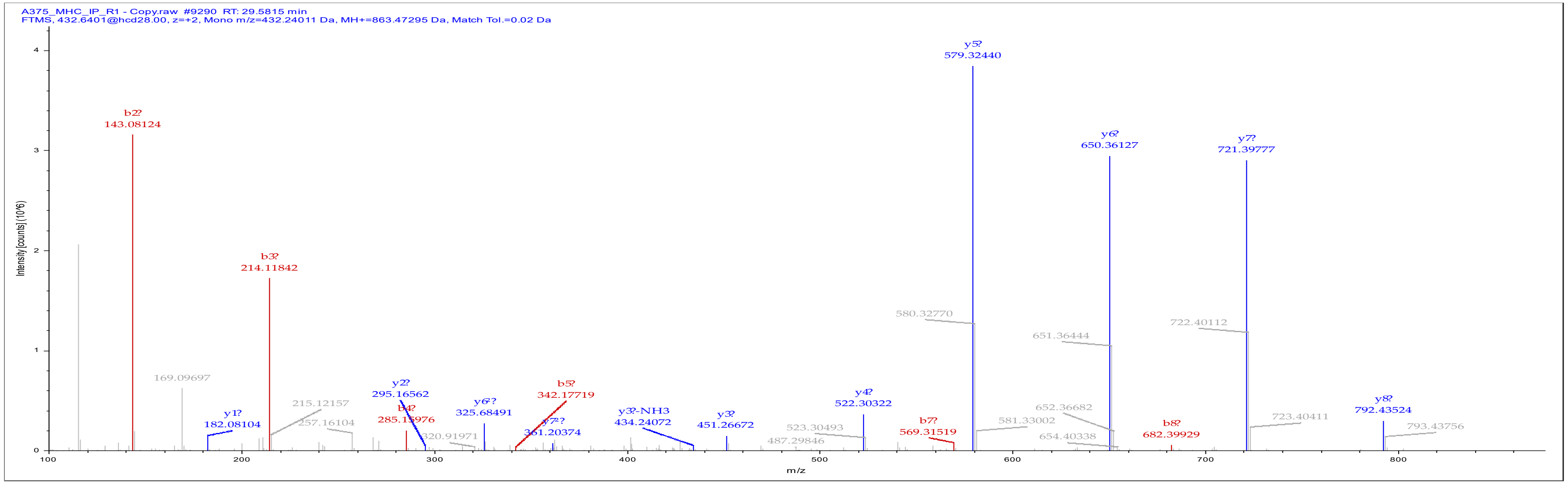

Spliced peptide - AAALKAAAF

Synthetic

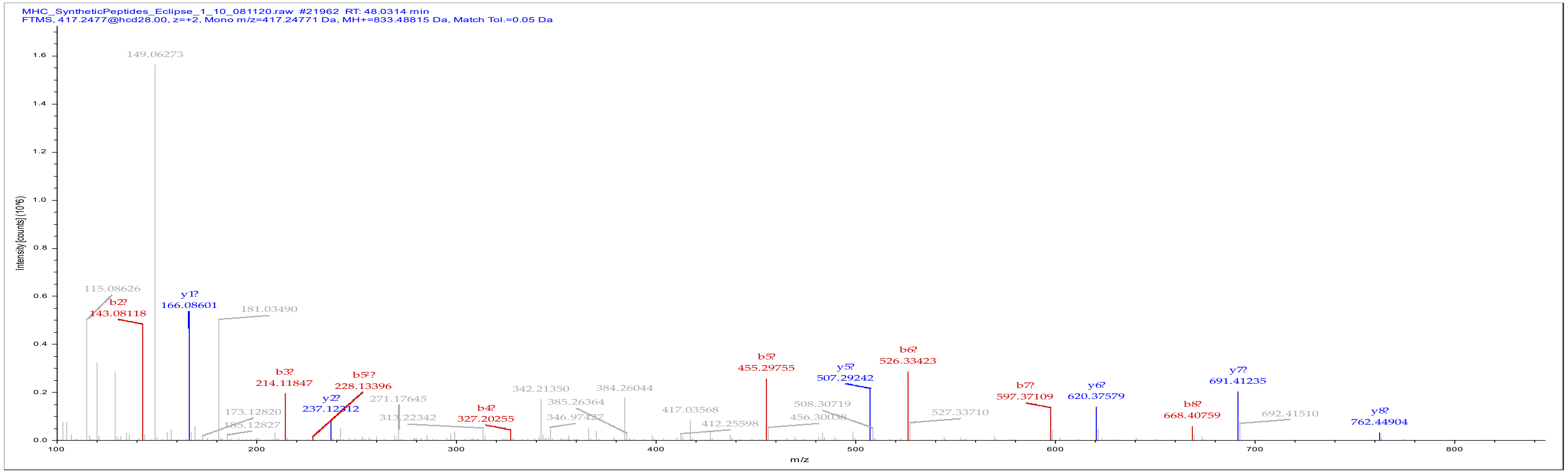

Experimental

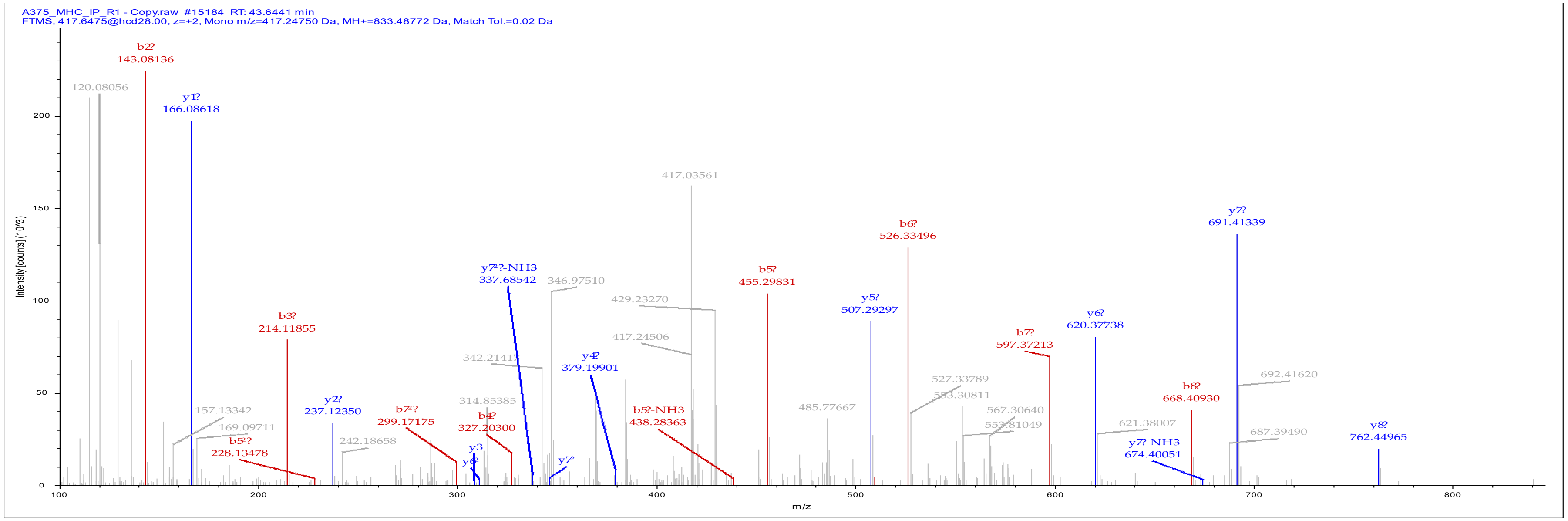

Spliced peptide - ASDLLLHSY

Synthetic

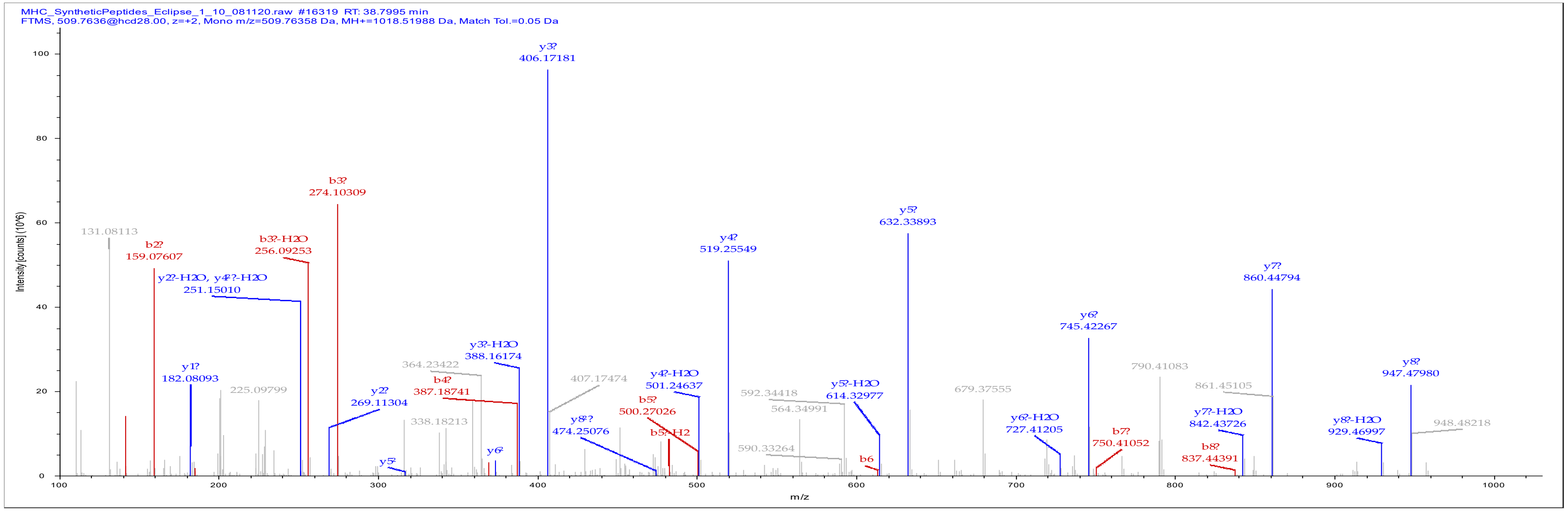

Experimental

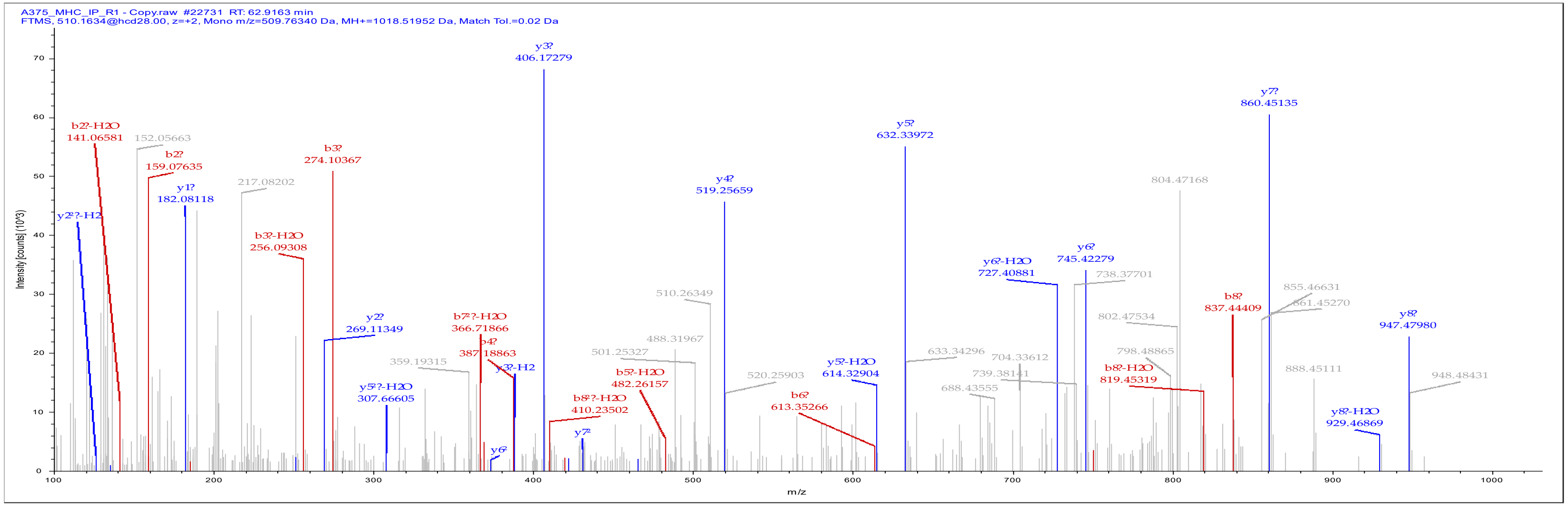

Spliced peptide - ASELHTSLY

Synthetic

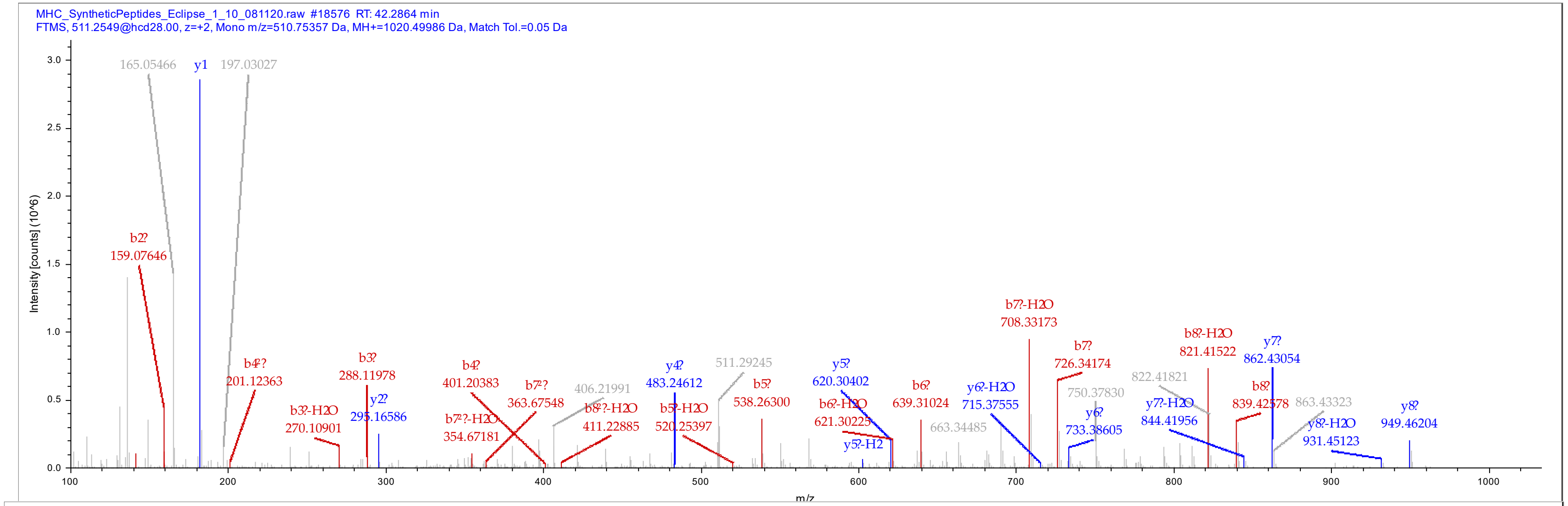

Experimental

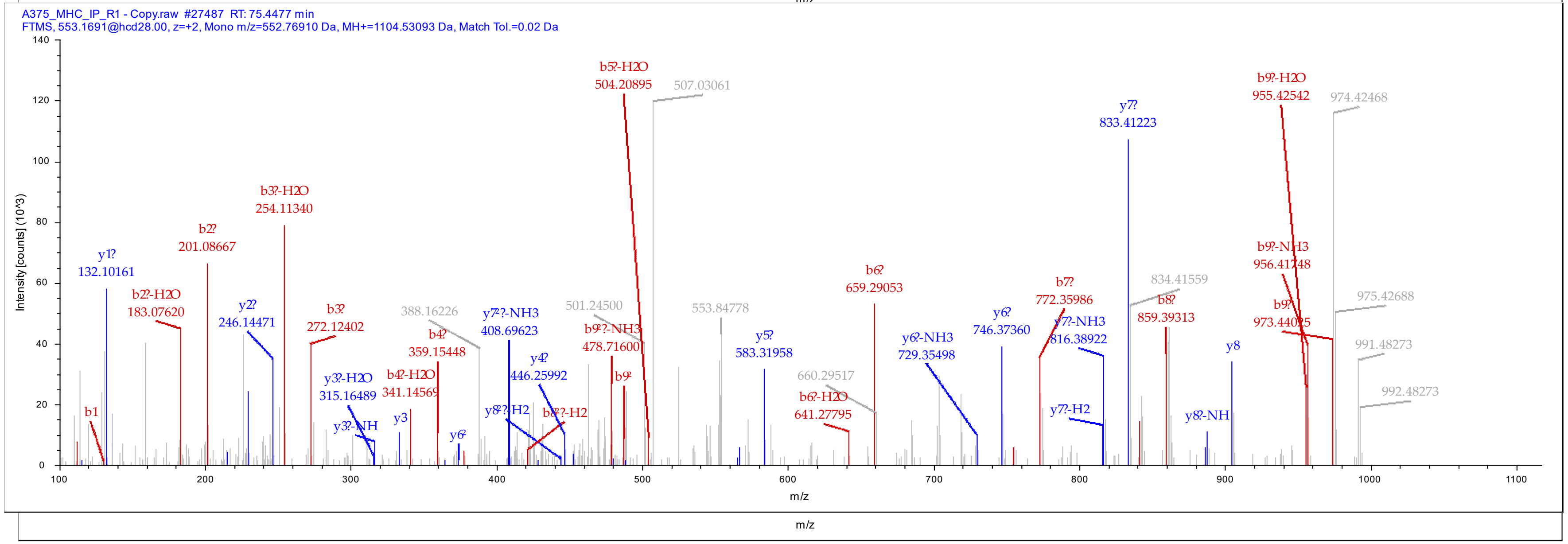

Spliced peptide - EAASYHLSNL

Synthetic

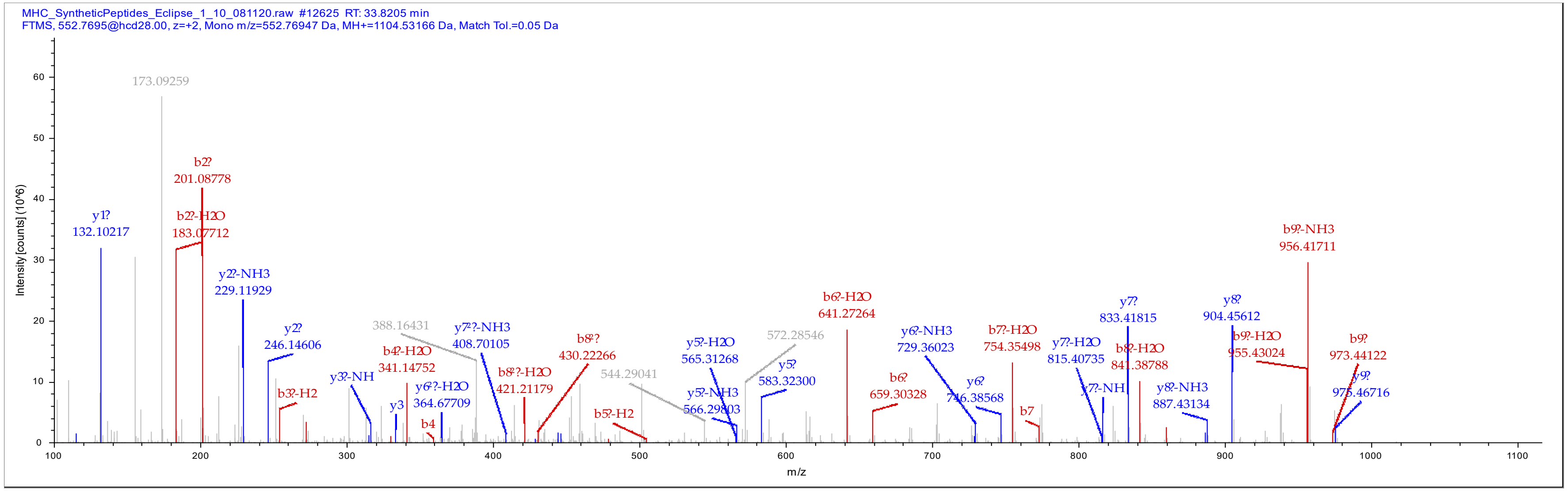

Experimental

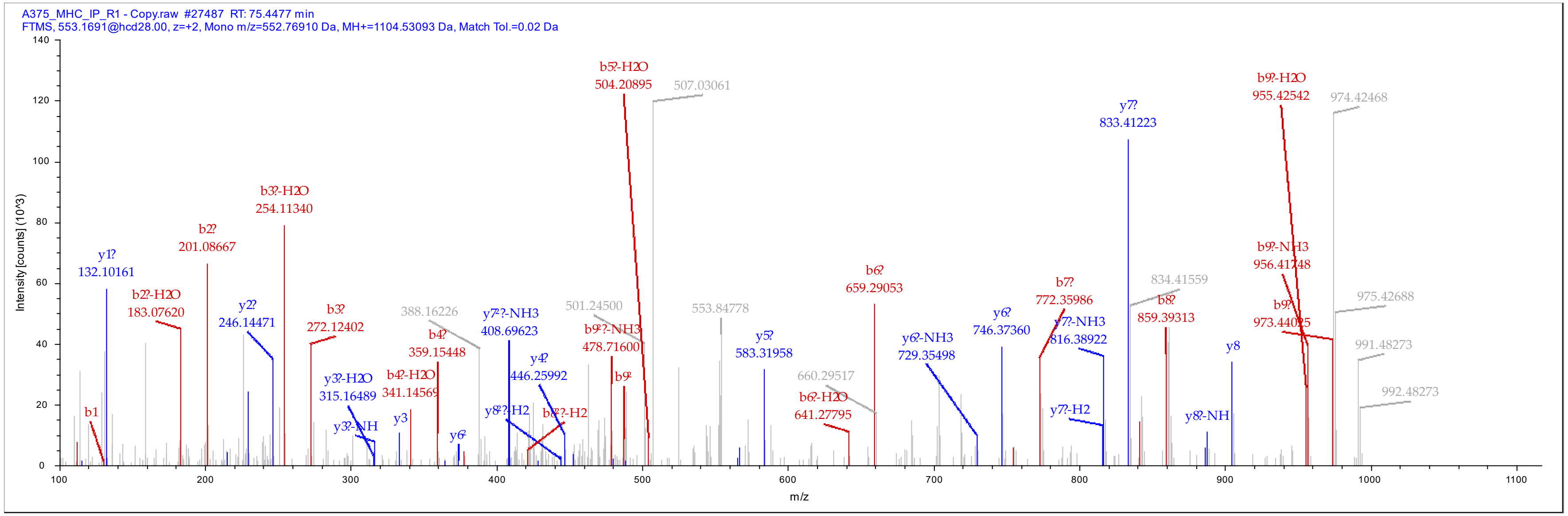

Spliced peptide - EELAAKLKF

Synthetic

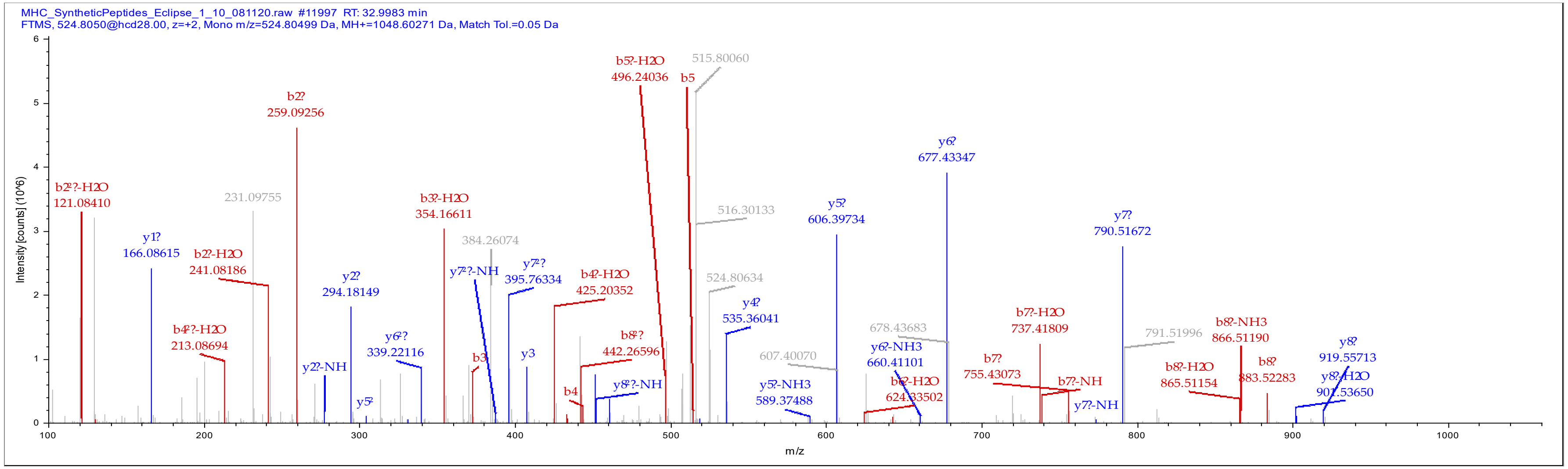

Experimental

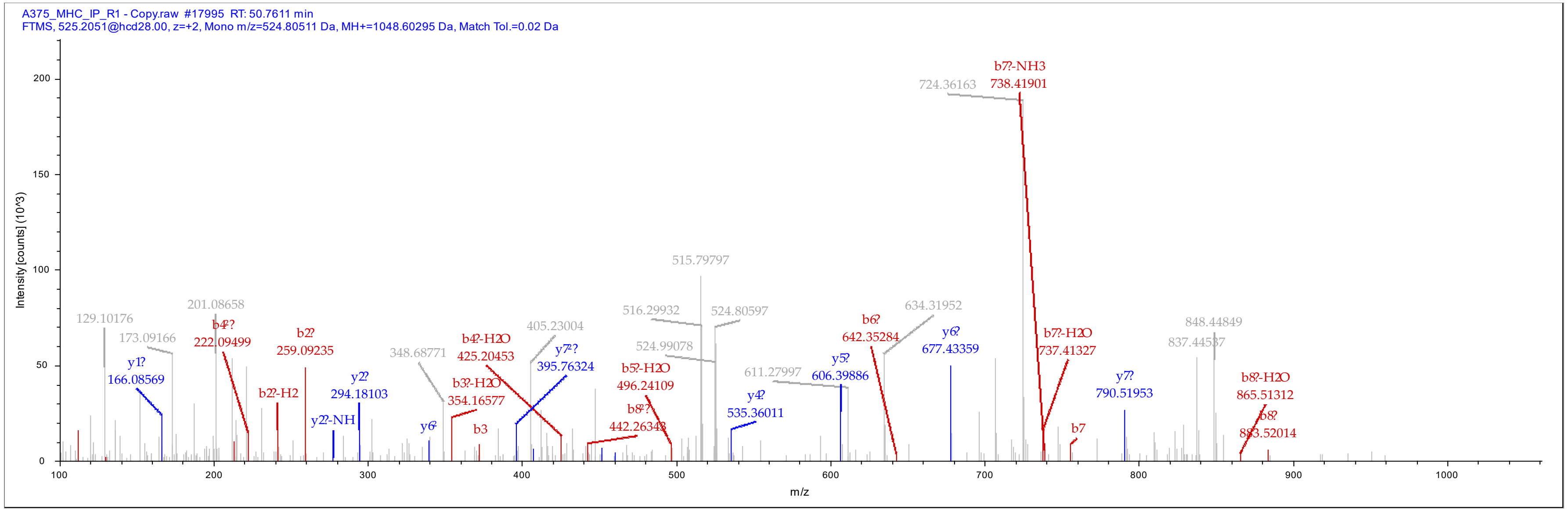

Spliced peptide - EENLYFKNF

Synthetic

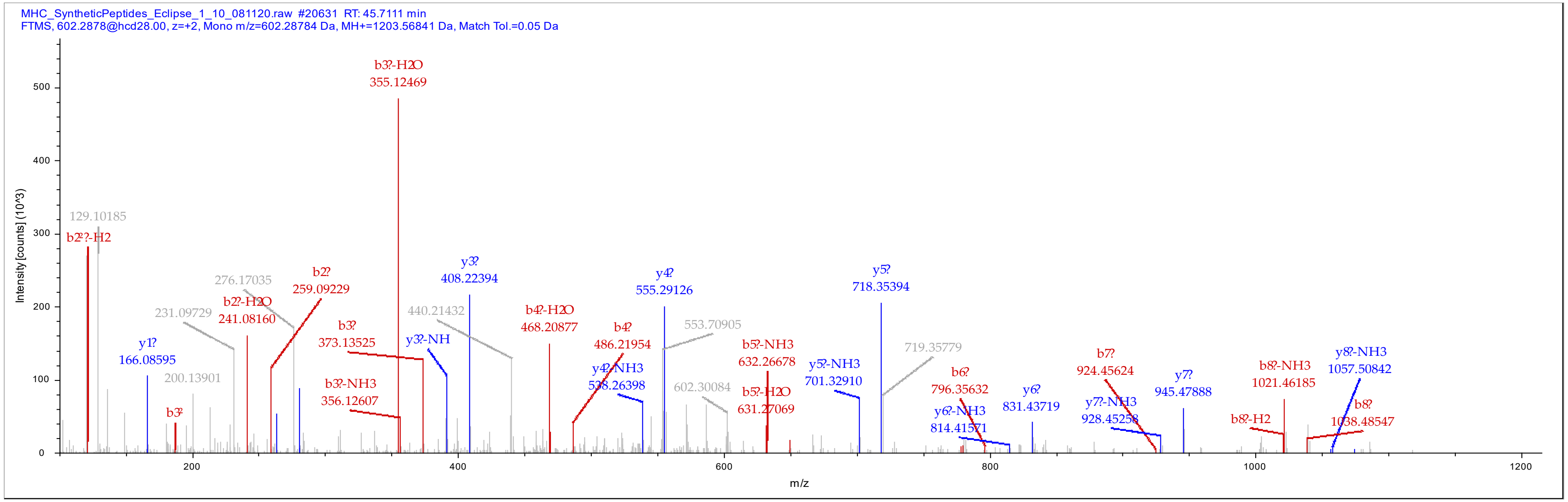

Experimental

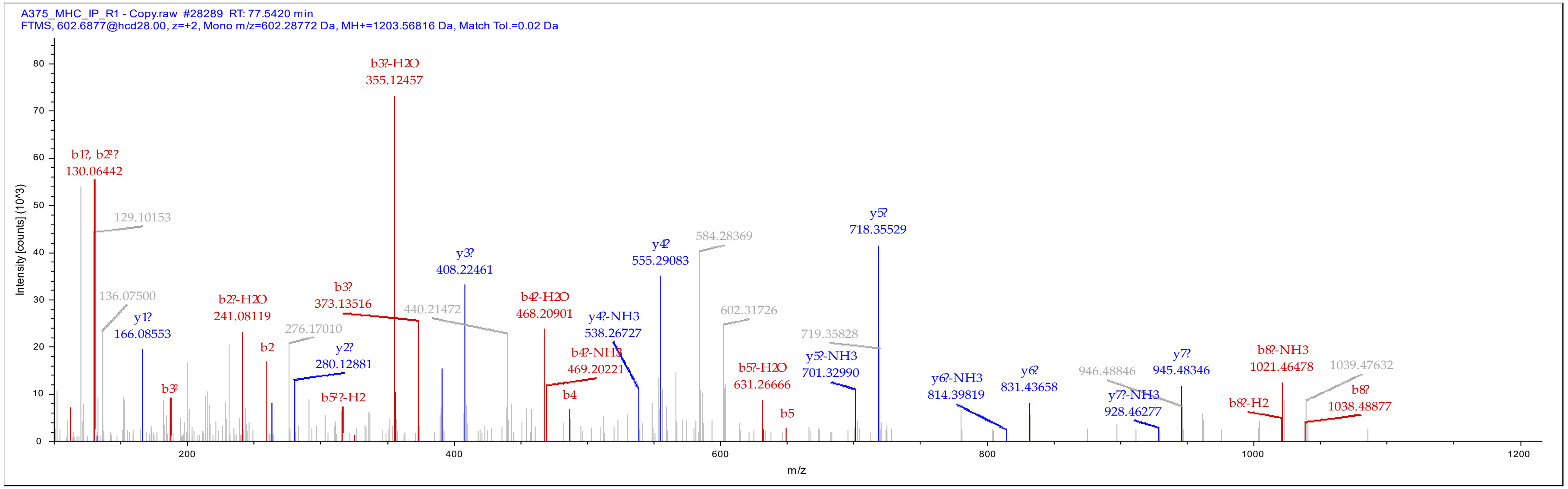

Spliced peptide - ELEDGRTLSDY

Synthetic

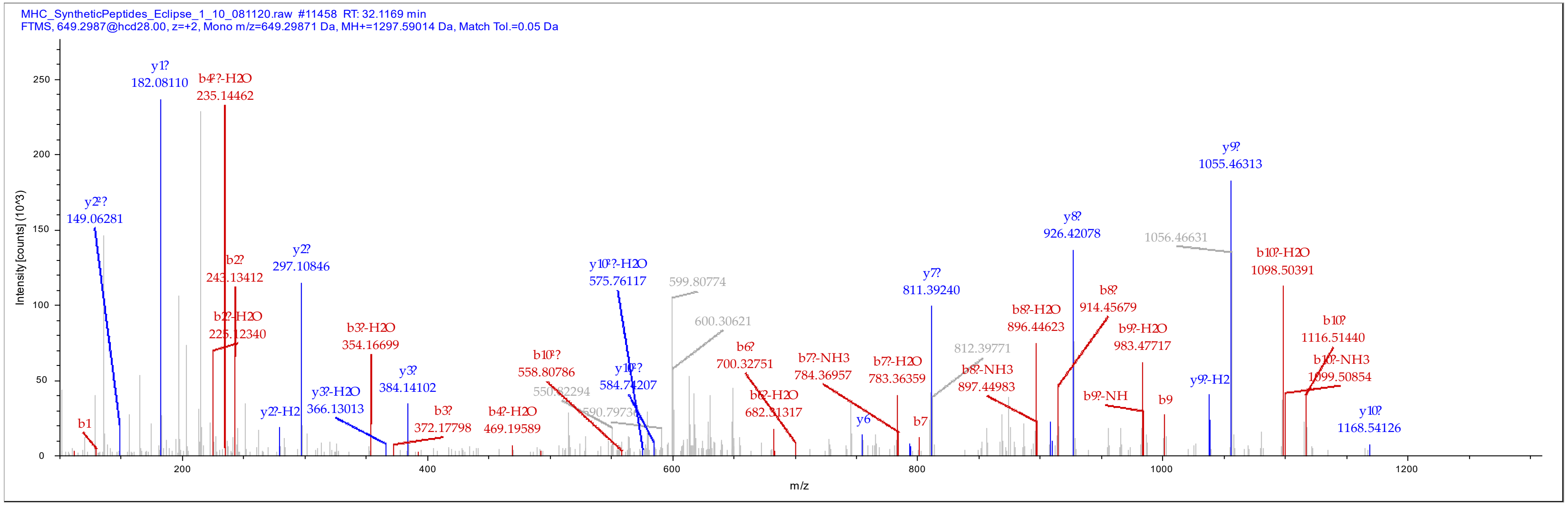

Experimental

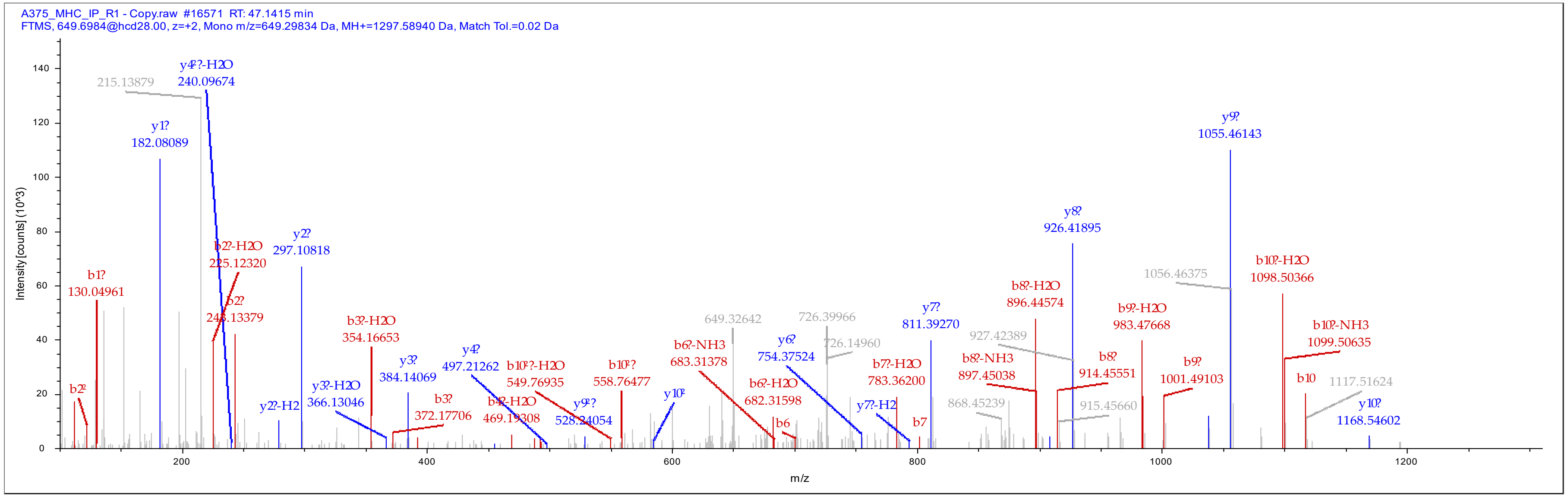

Spliced peptide - FANYLDKVL

Synthetic

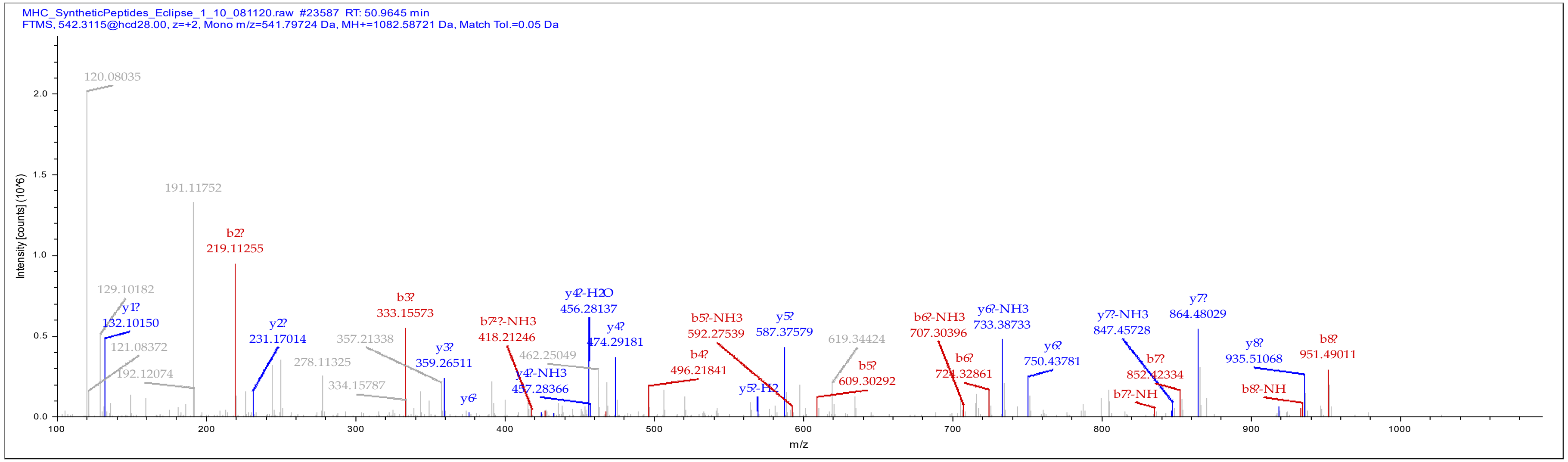

Experimental

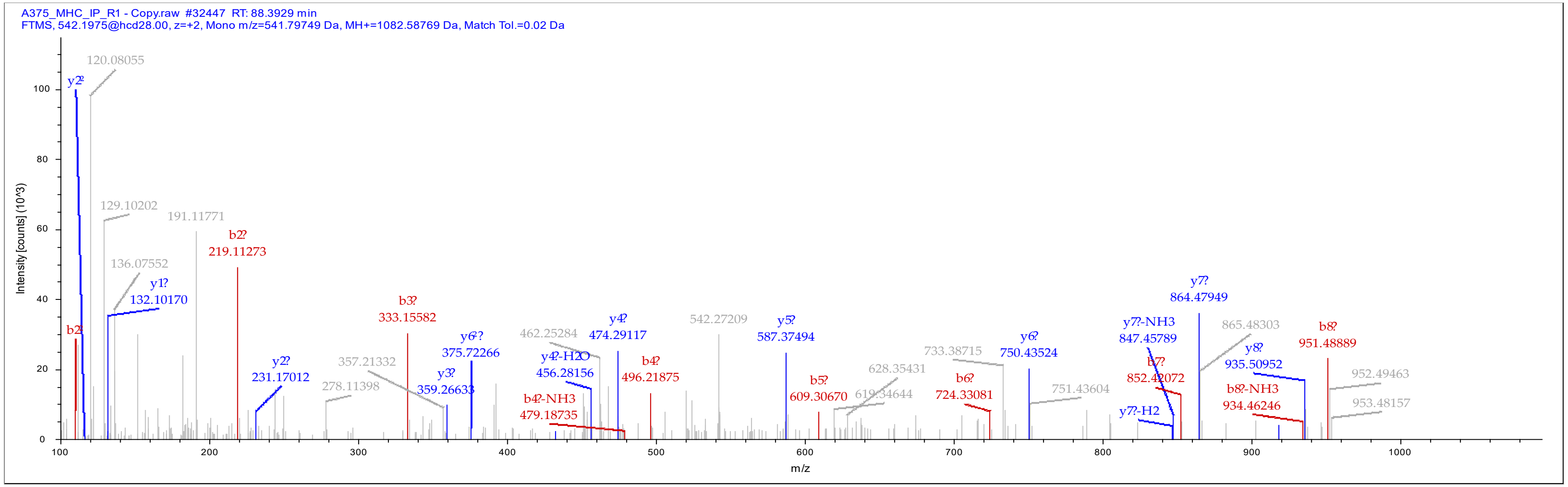

Spliced peptide - FLFQDFLRQA

Synthetic

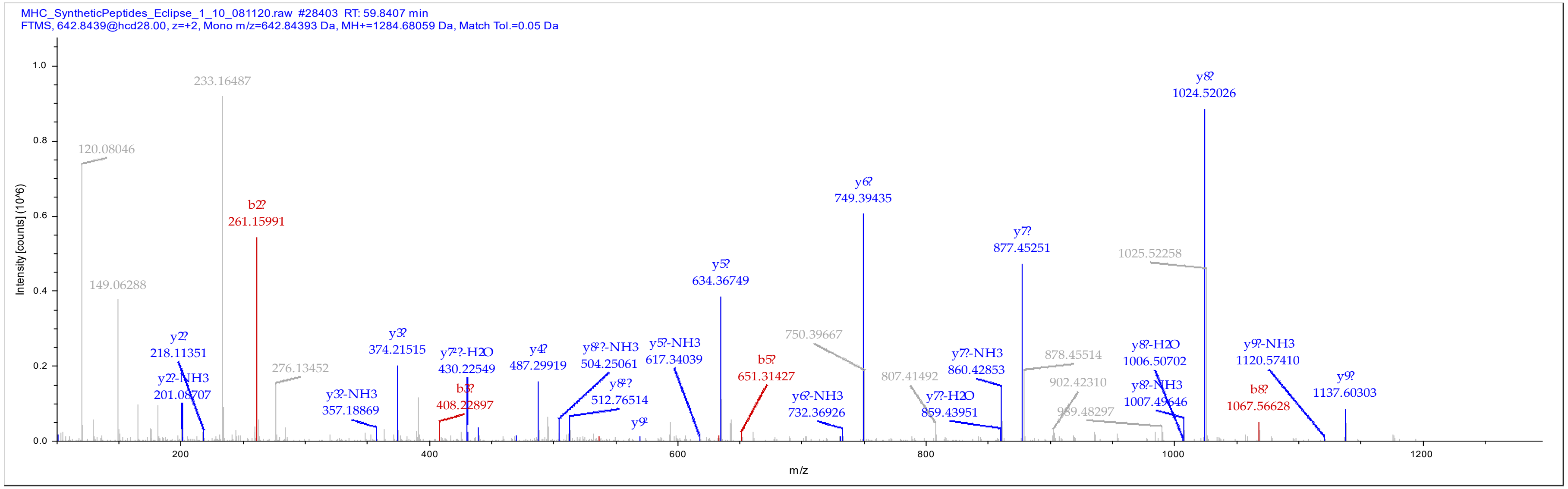

Experimental

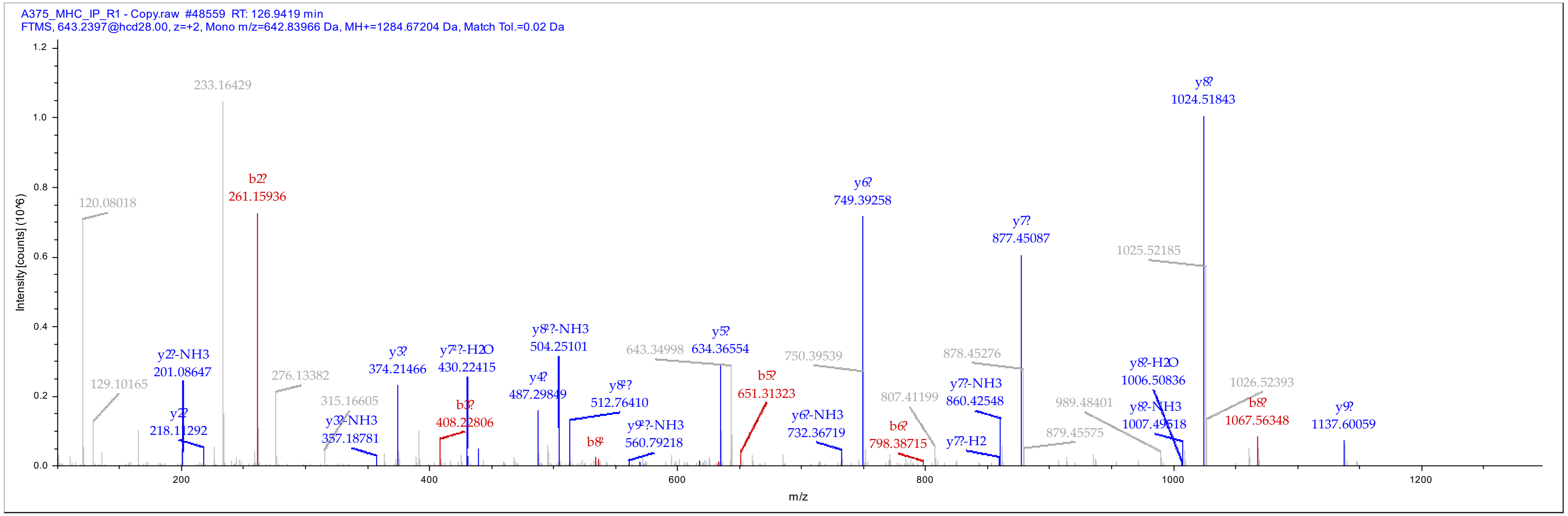

Spliced peptide - FLLQHLPLV

Synthetic

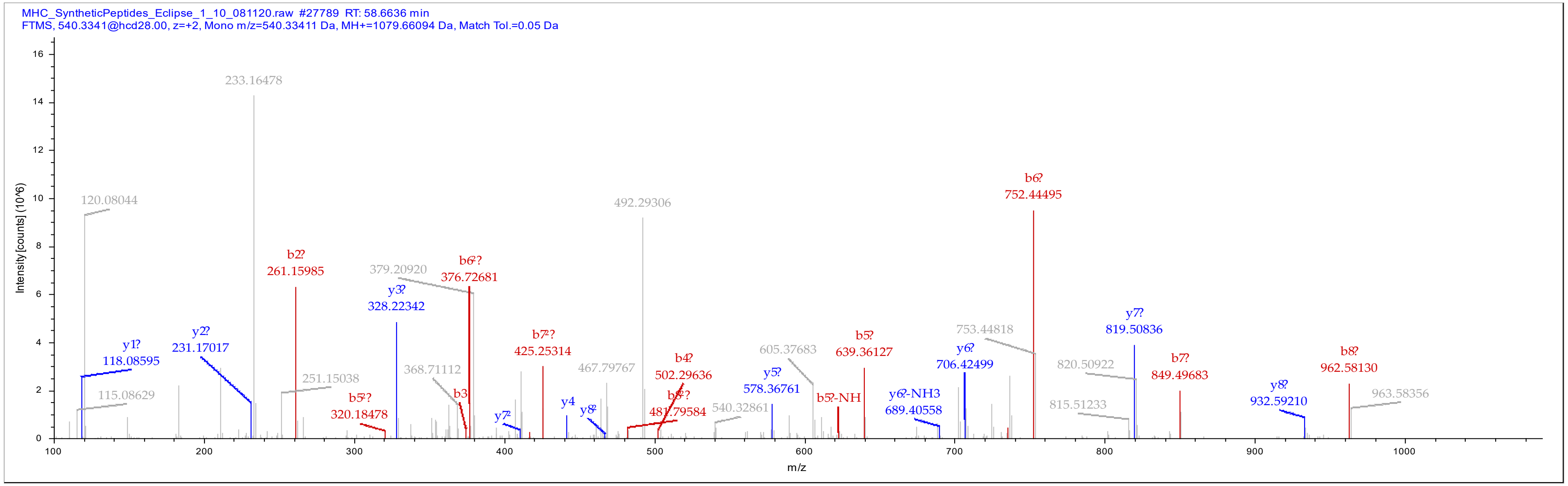

Experimental

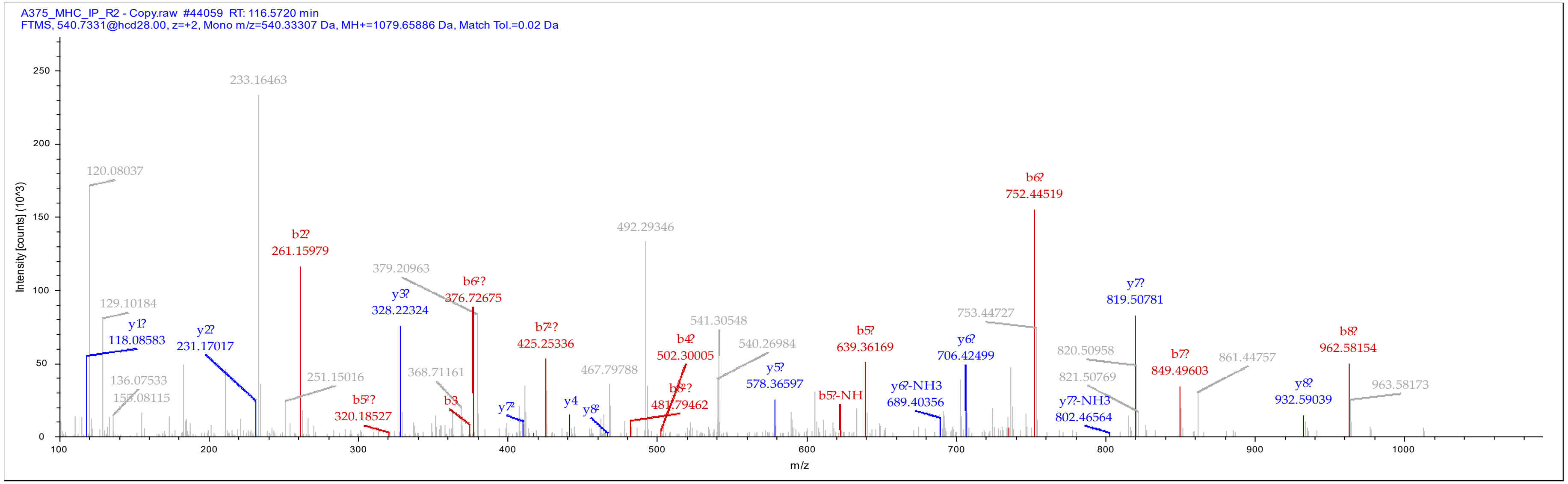

Spliced peptide - FQDPYTRSL

Synthetic

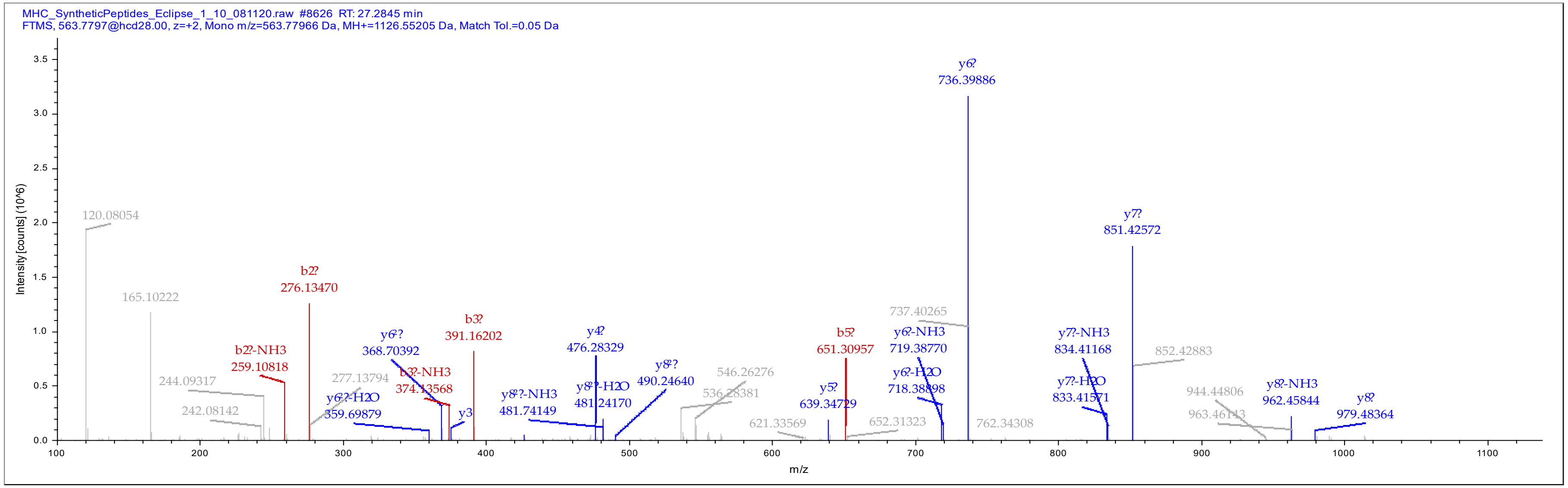

Experimental

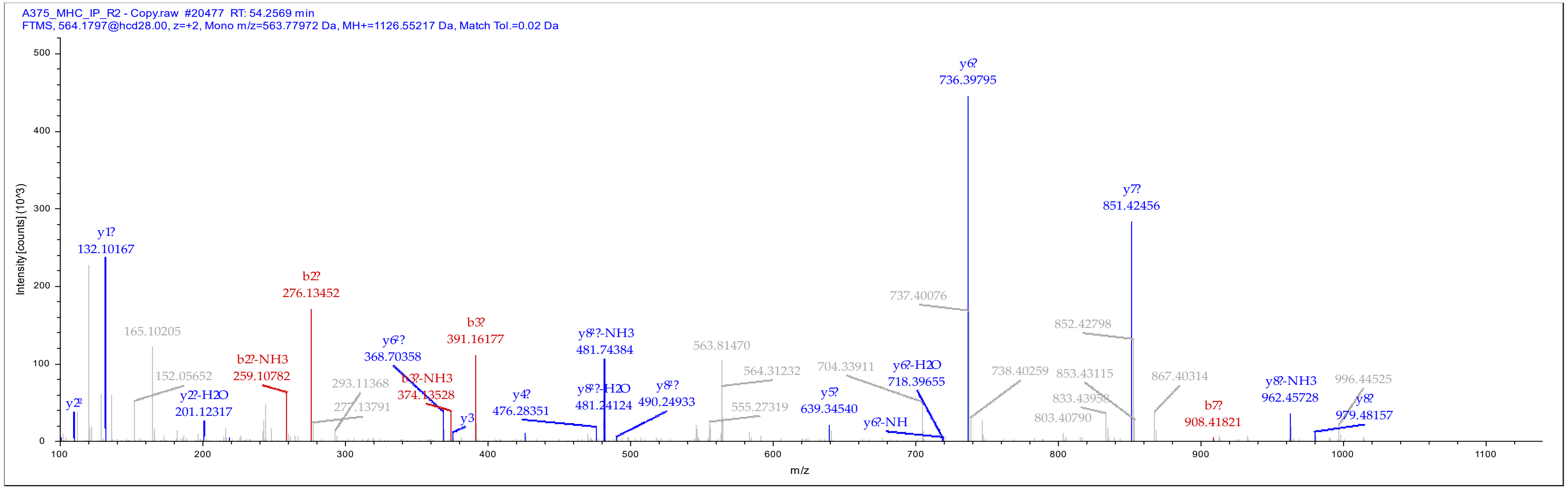

Spliced peptide - HALPLLKEY

Synthetic

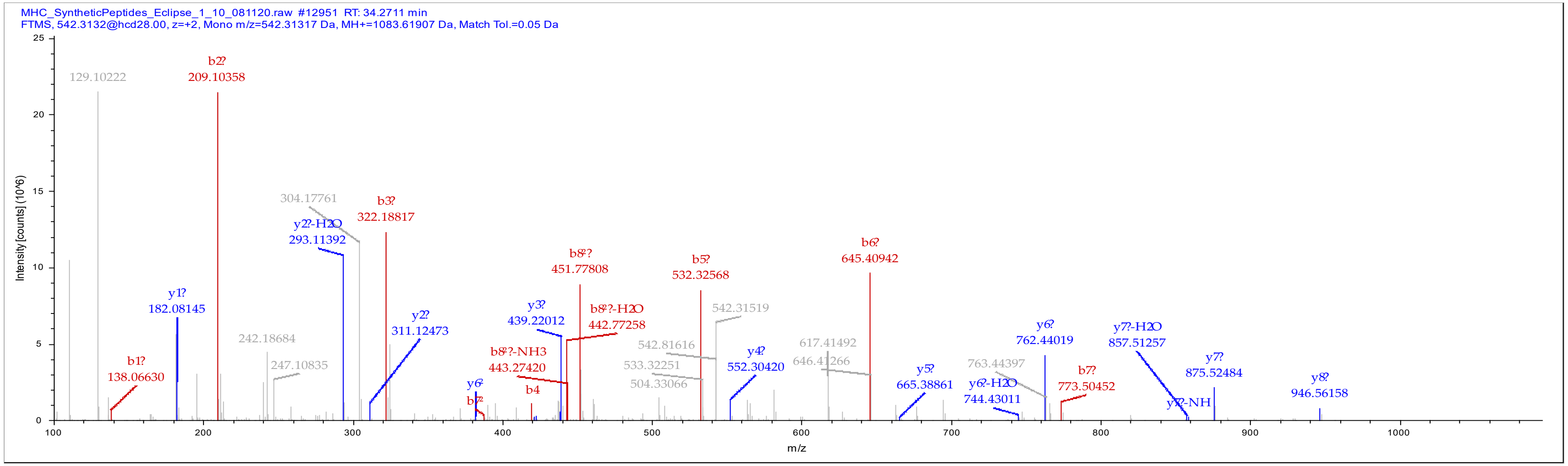

Experimental

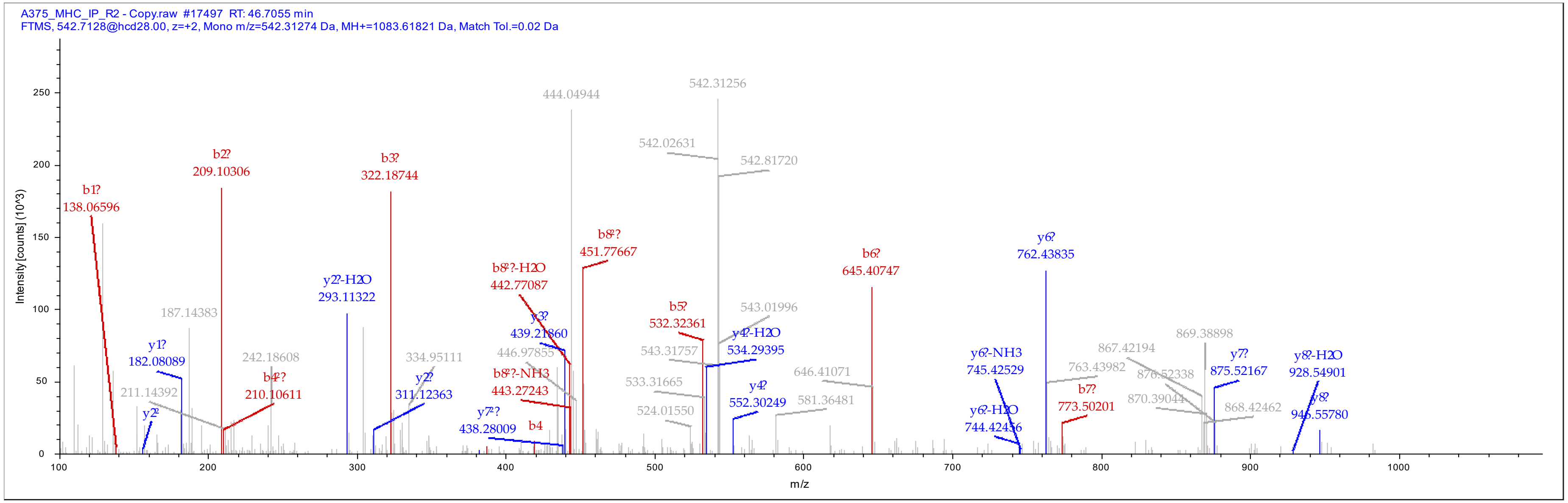

Spliced peptide - HVVDLVRQL

Synthetic

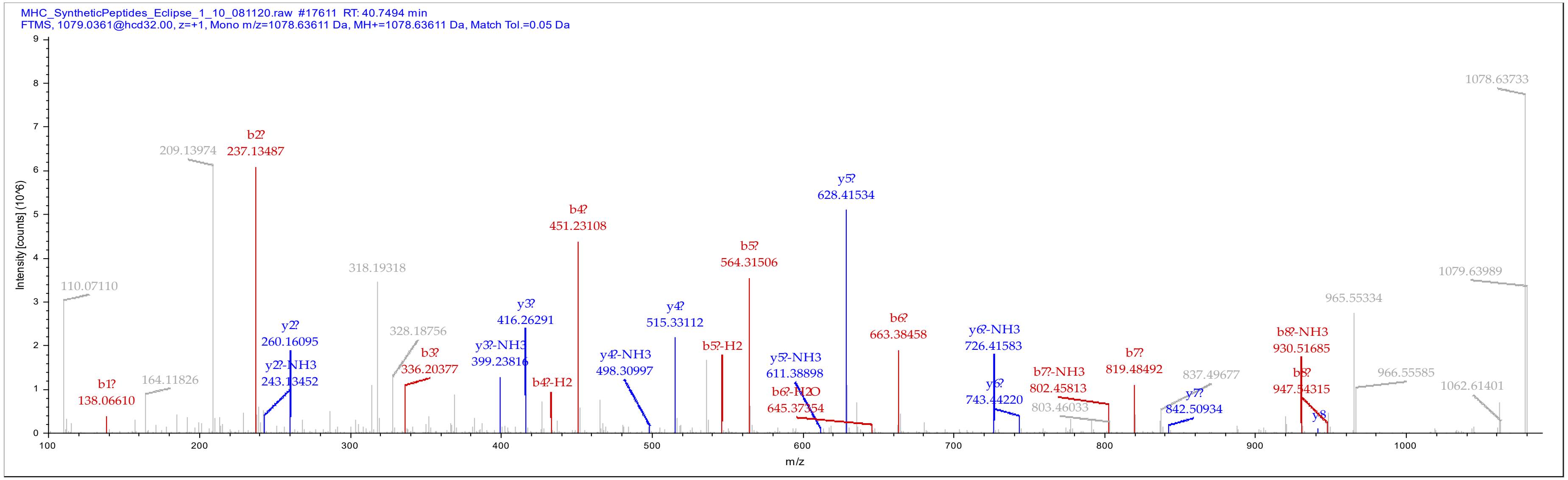

Experimental

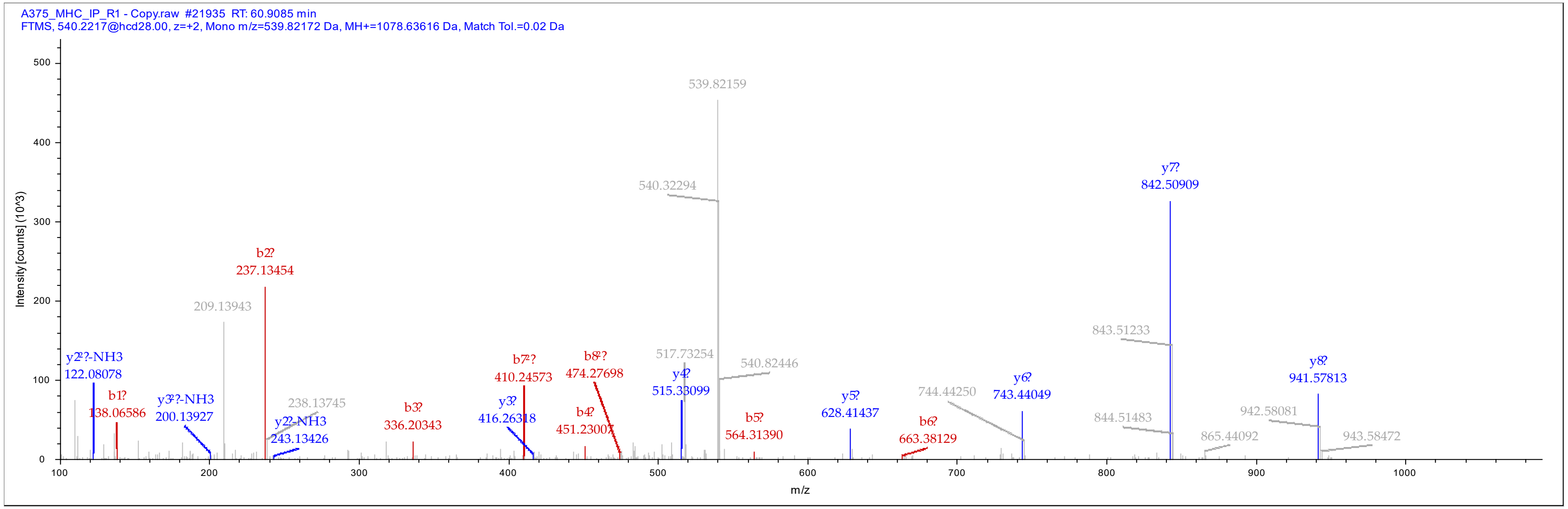

Spliced peptide - KVLDLLRKF

Synthetic

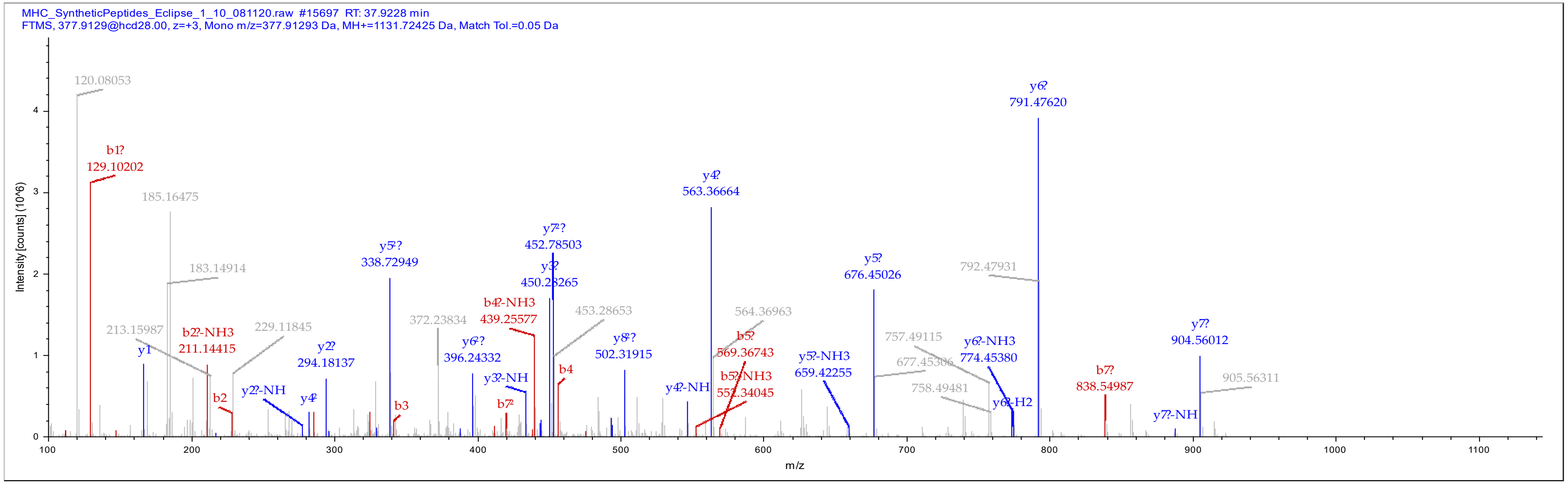

Experimental

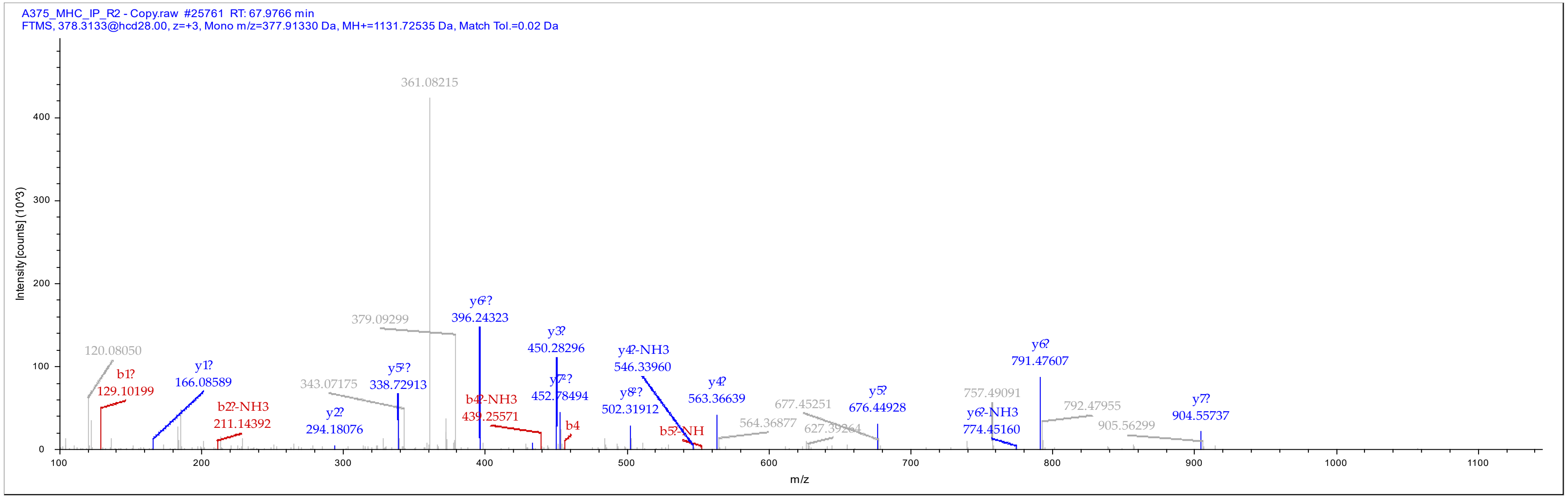

Spliced peptide - LELLLHKNF

Synthetic

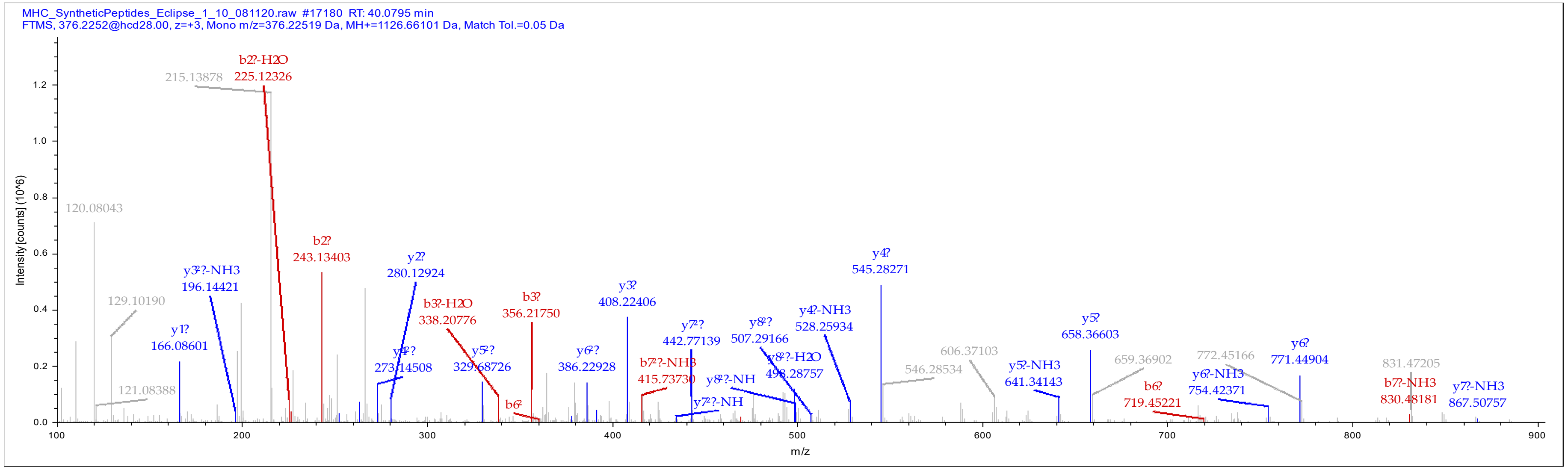

Experimental

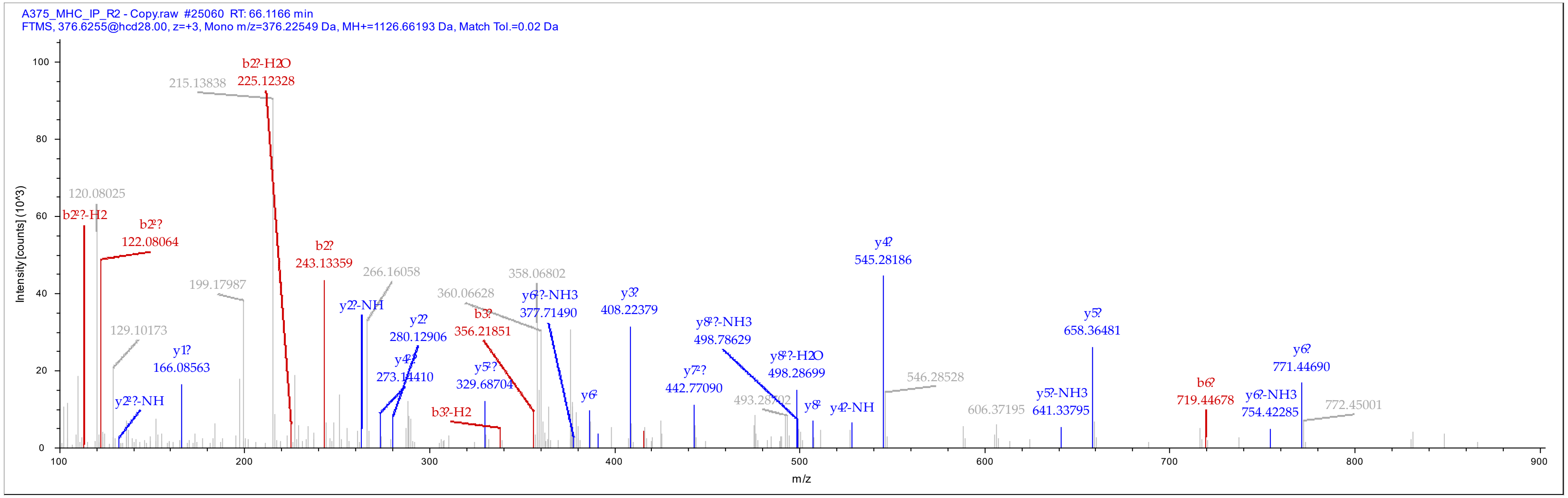

Spliced peptide - LERAFLKKL

Synthetic

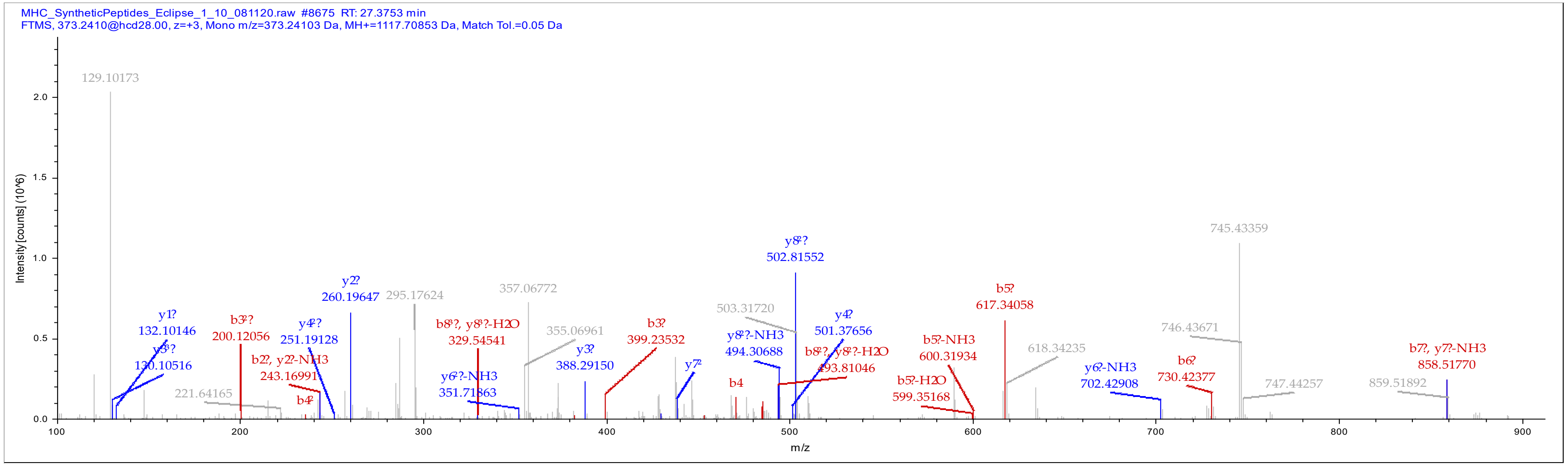

Experimental

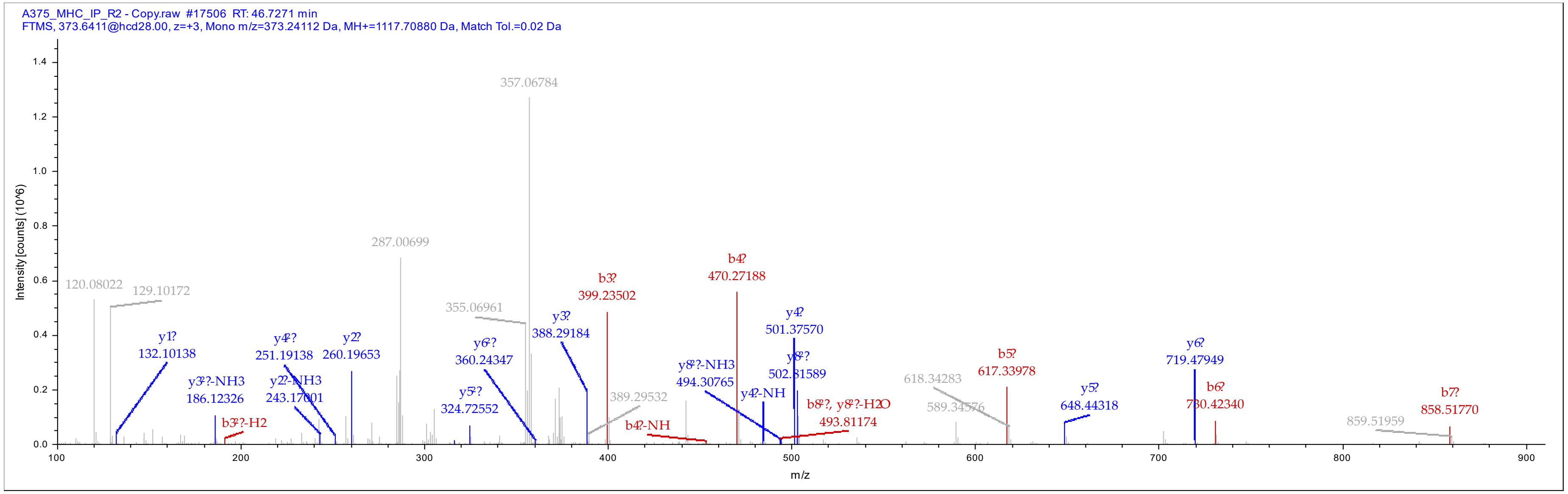

Spliced peptide - LGGGDFLQKL

Synthetic

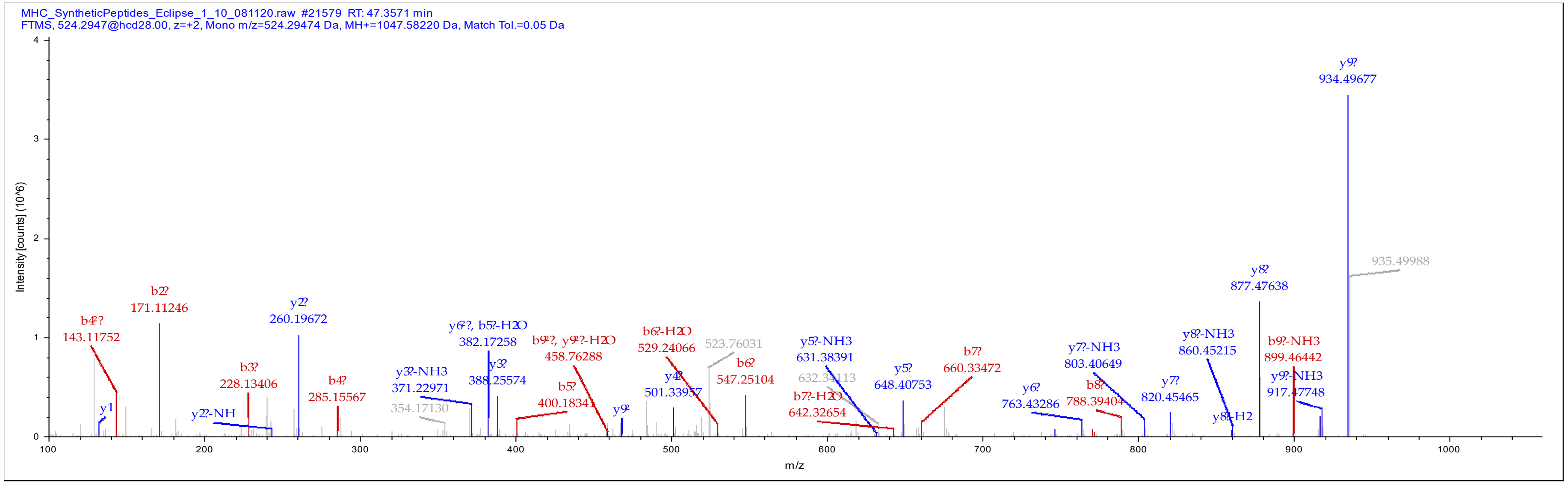

Experimental

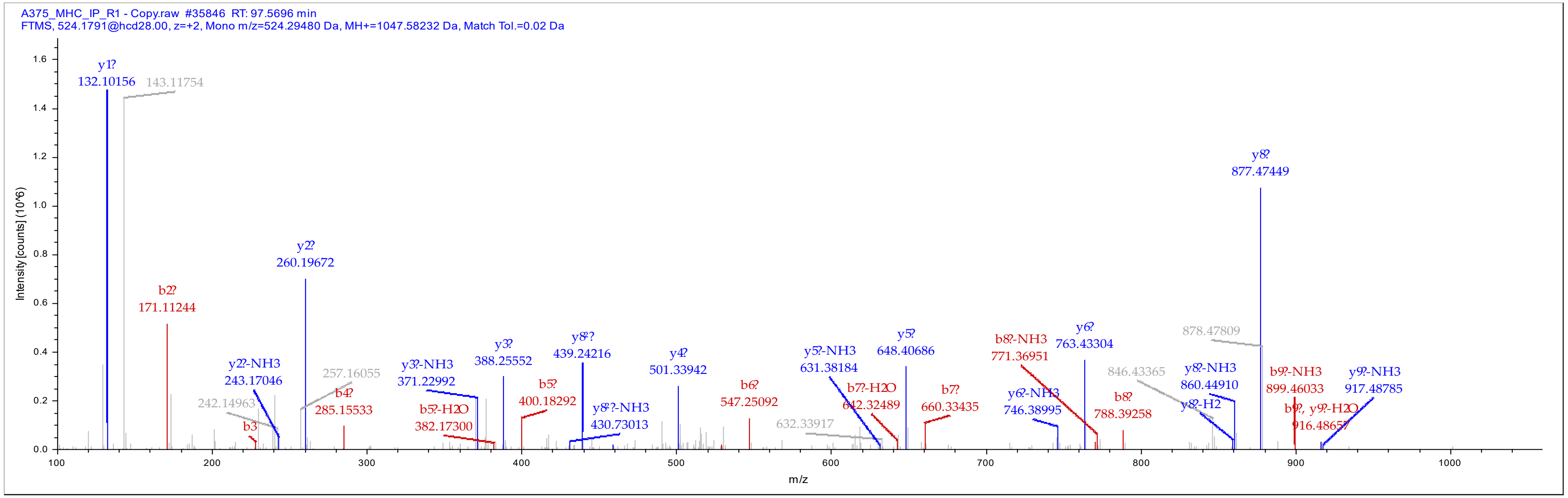

Spliced peptide - LGLEKGGLEL

Synthetic

Experimental

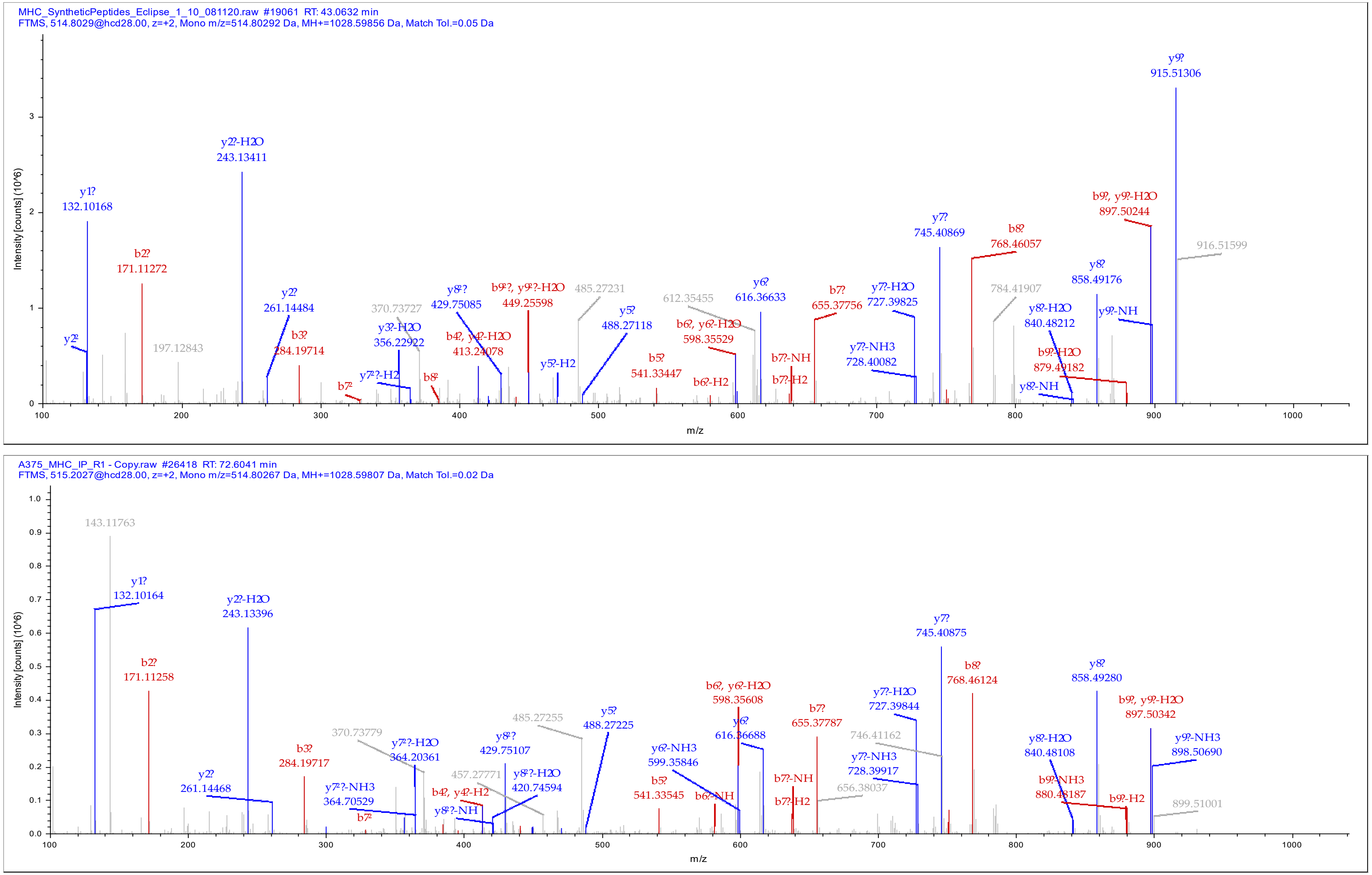

Spliced peptide - LRFDTQSKEKL

Synthetic

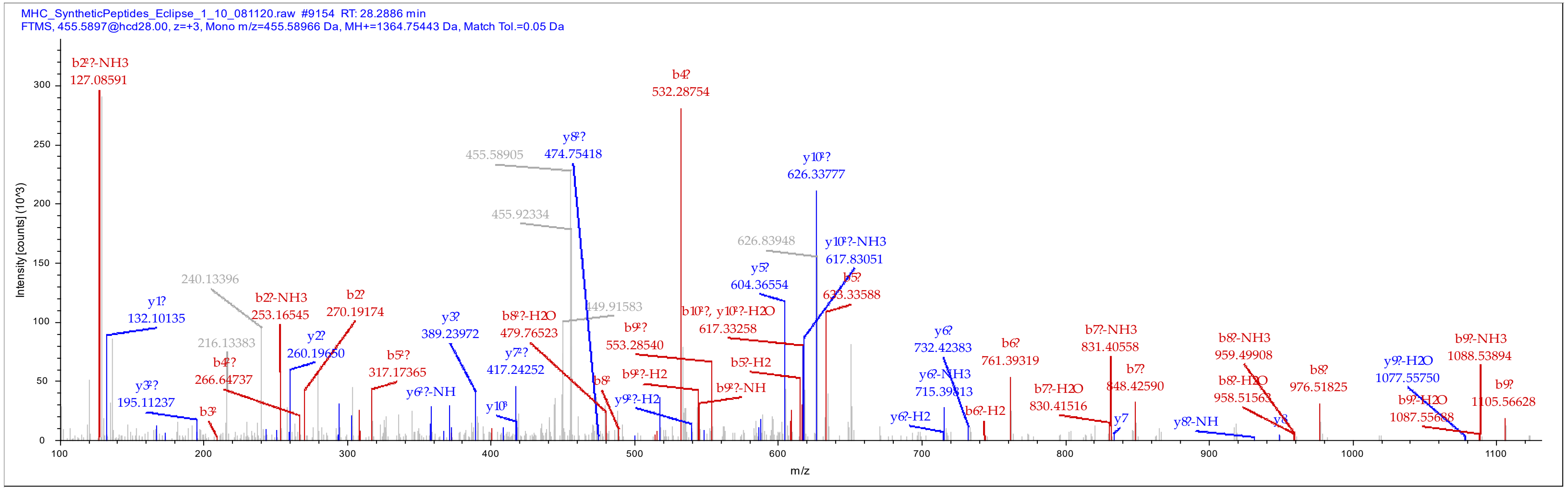

Experimental

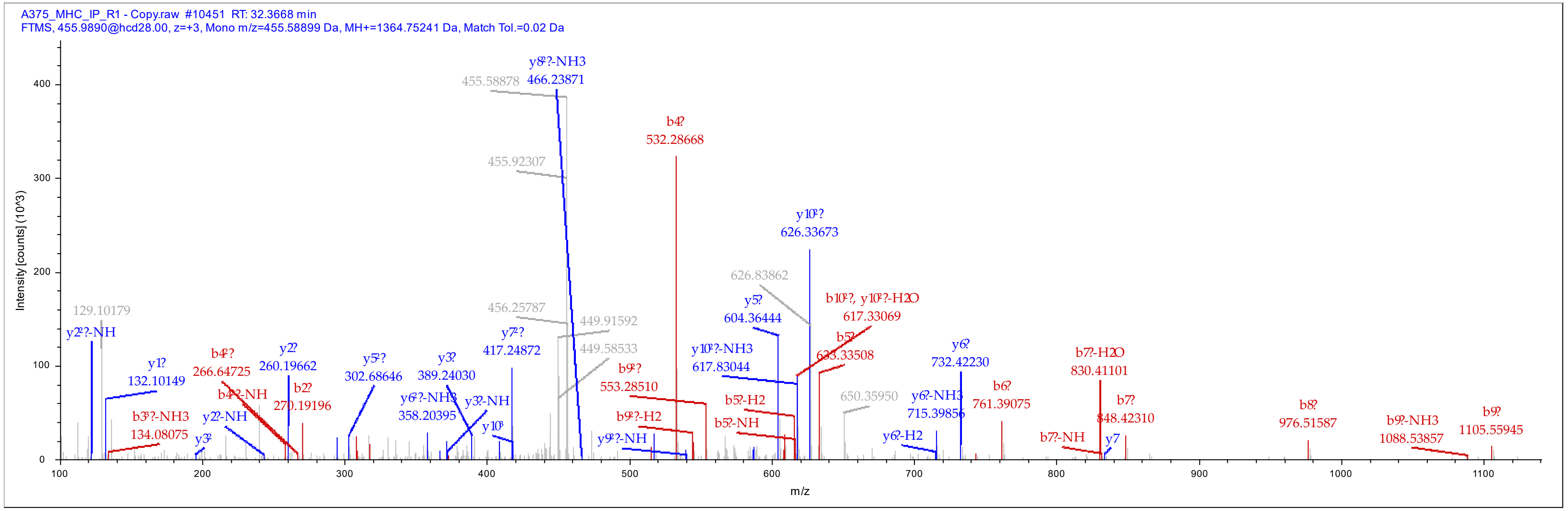

Spliced peptide - LTRPKLEKL

Synthetic

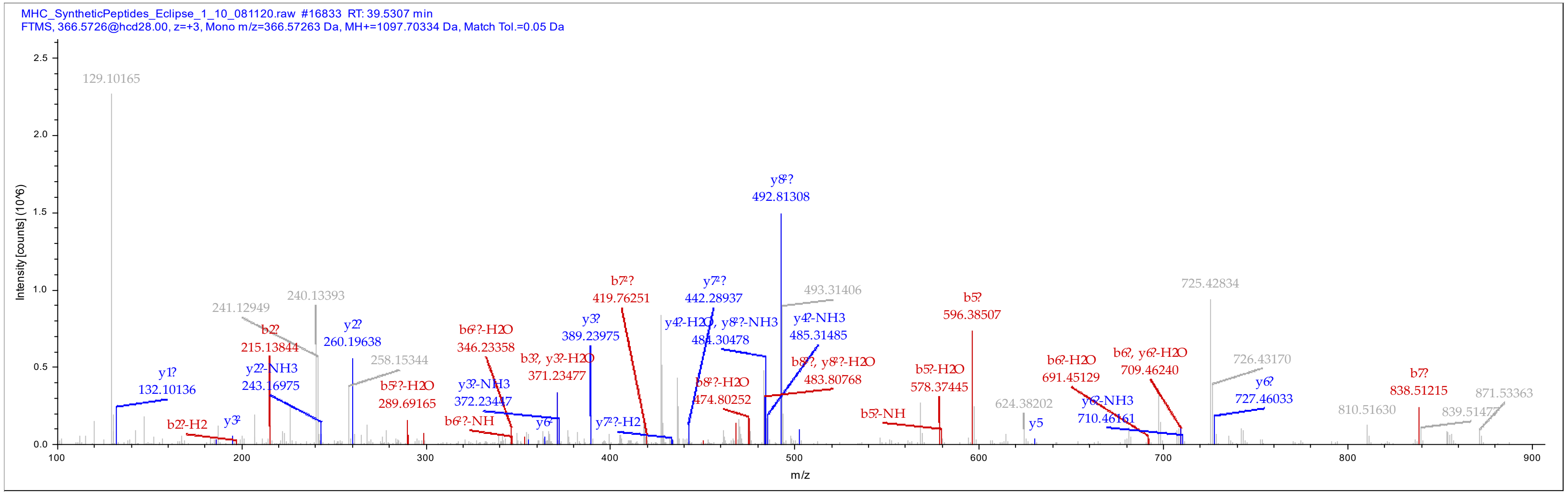

Experimental

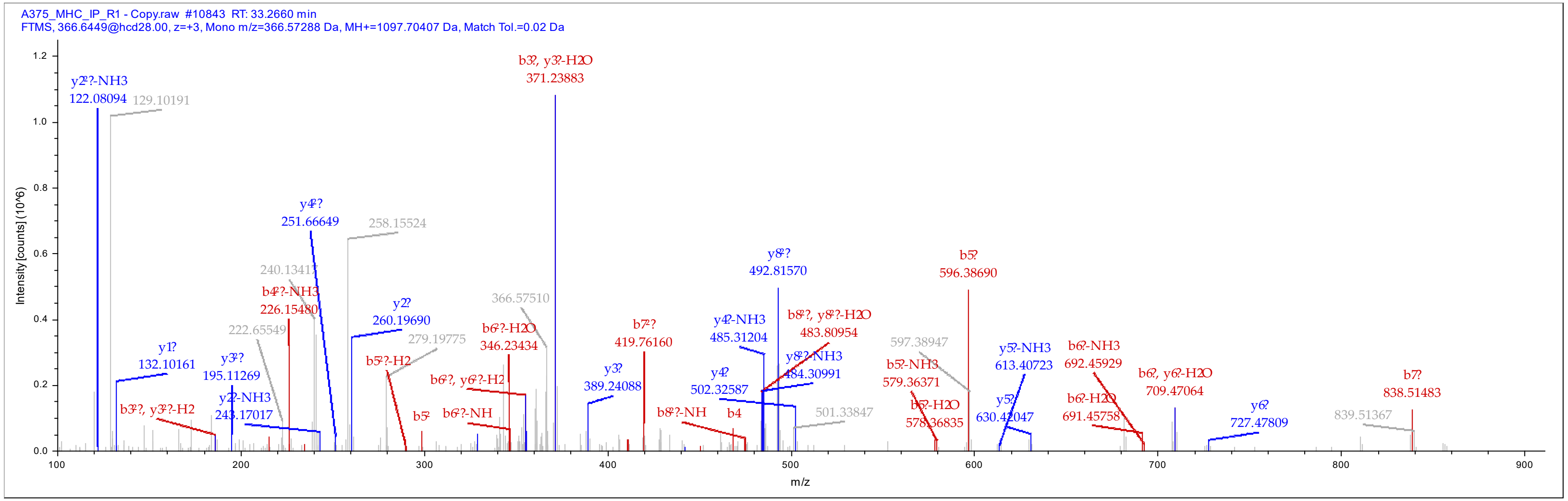

Spliced peptide - NMLPQLVRF

Synthetic

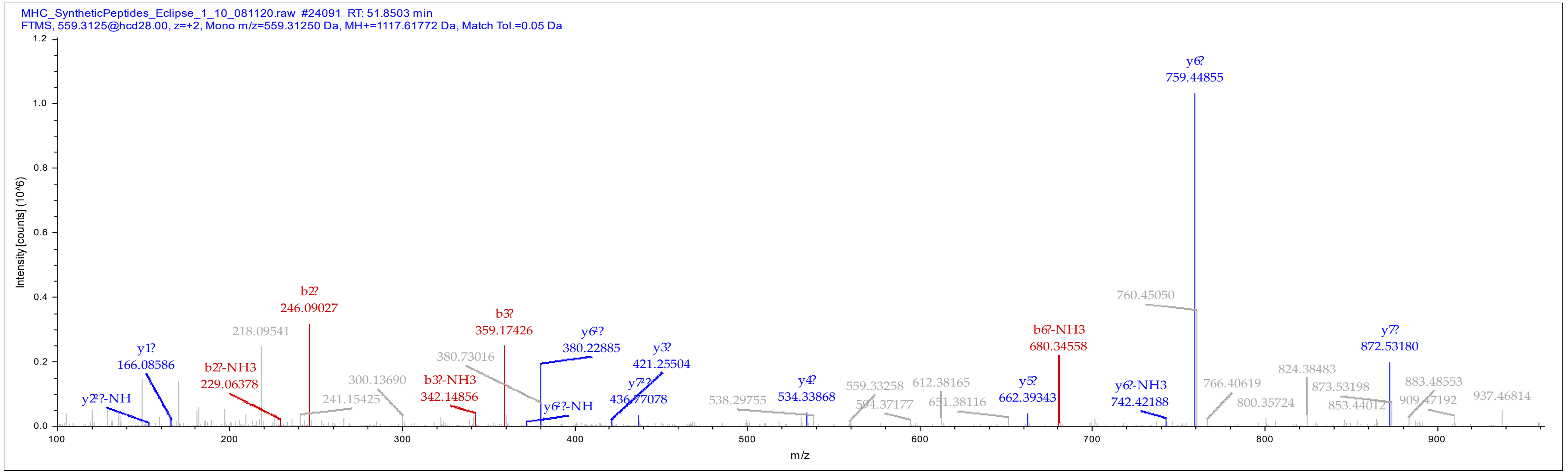

Experimental

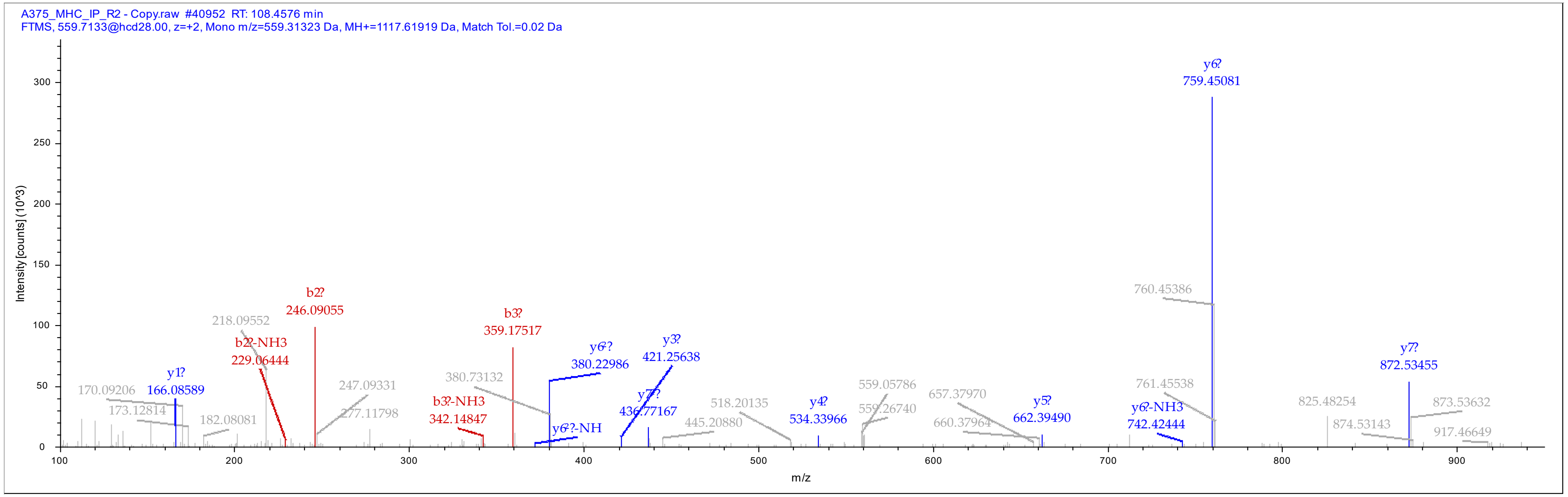

Spliced peptide - NSDLVLQRY

Synthetic

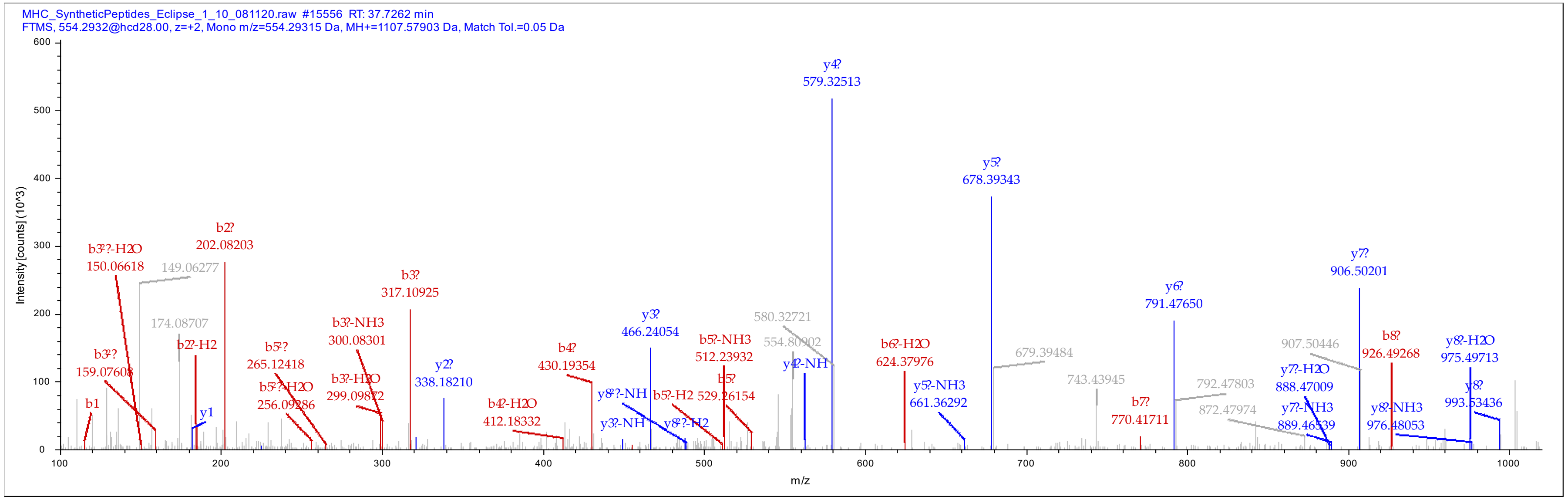

Experimental

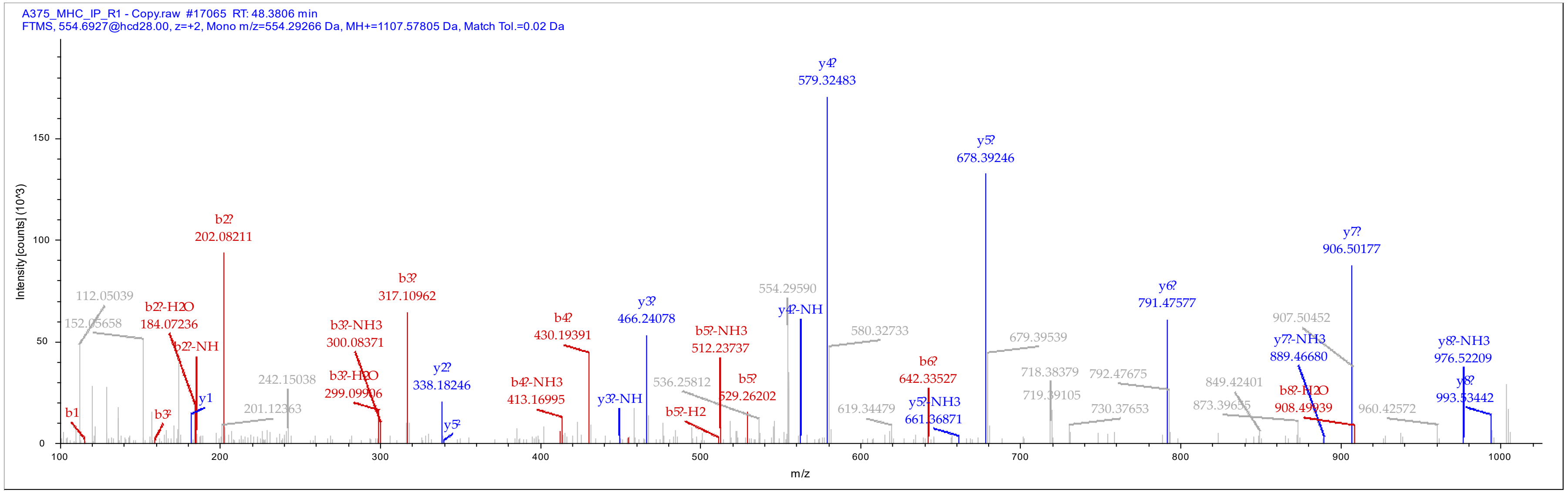

Spliced peptide - QEHPDLAKKF

Synthetic

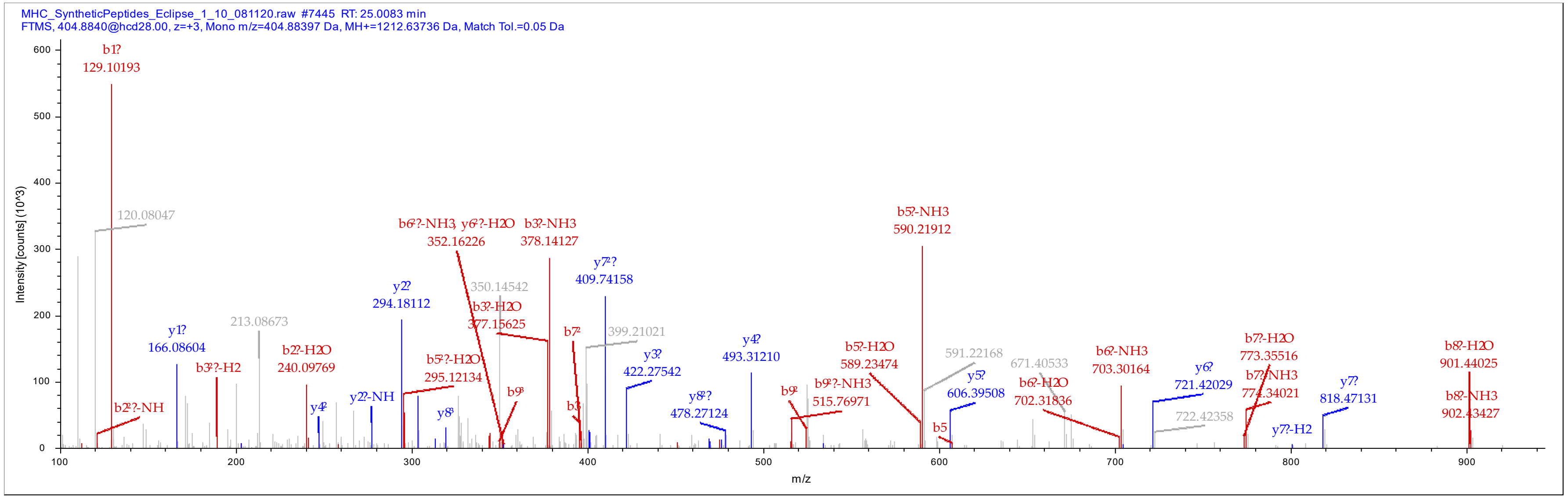

Experimental

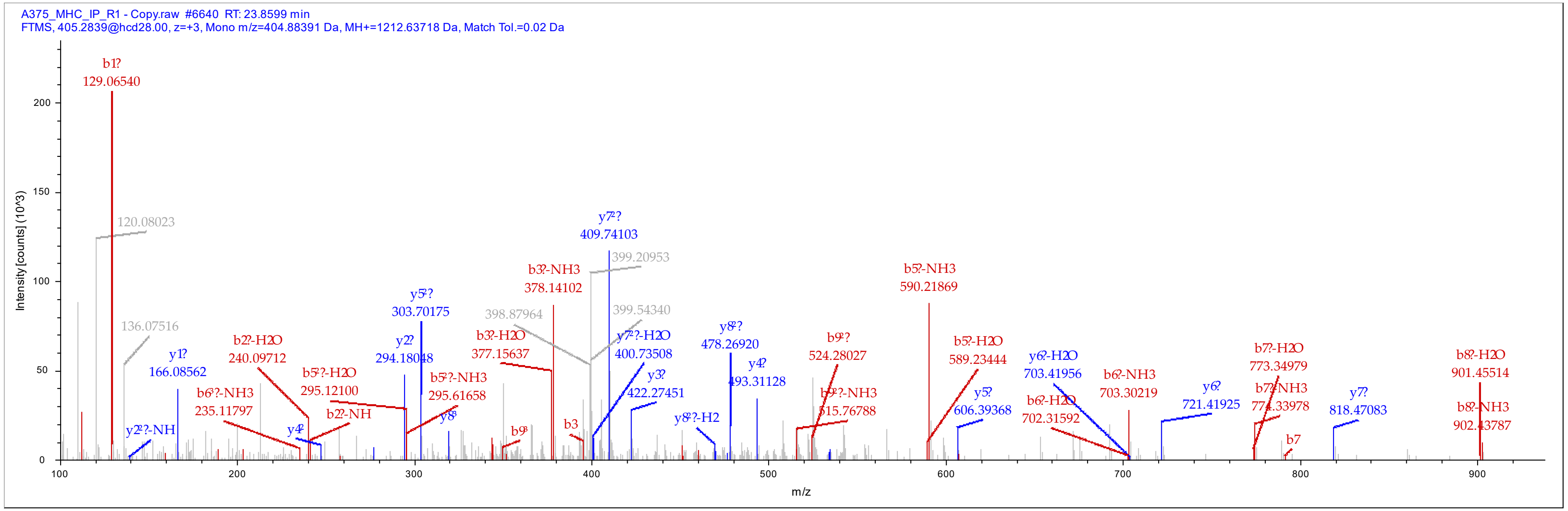

Spliced peptide - REELFERKY

Synthetic

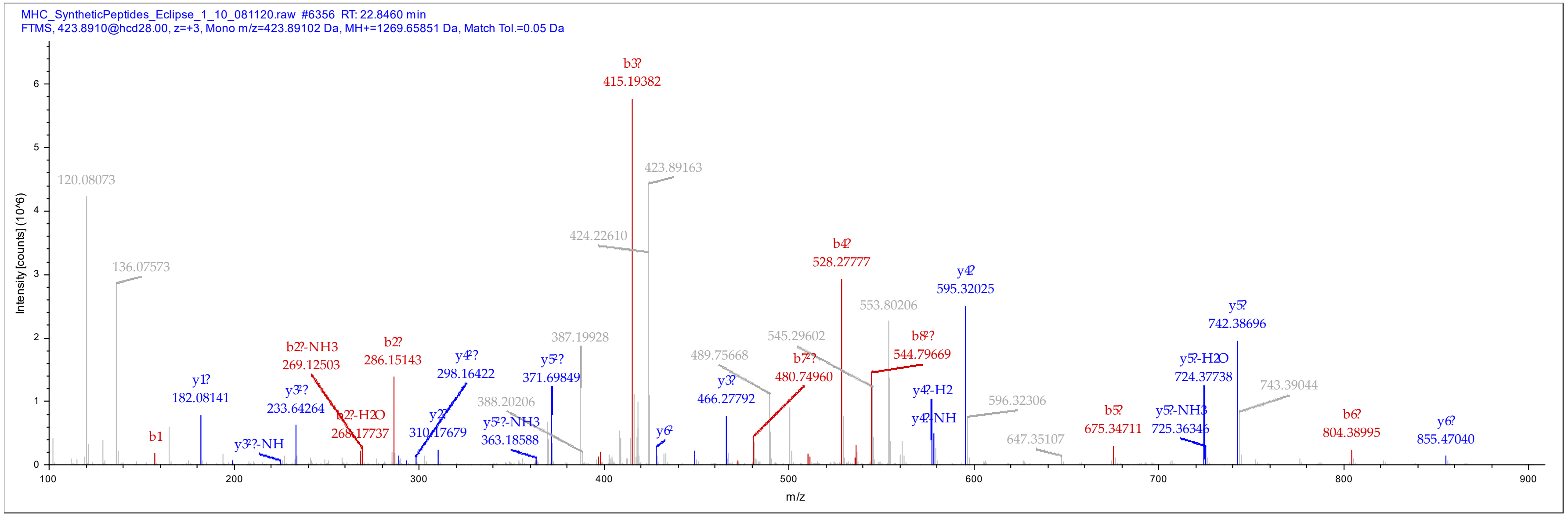

Experimental

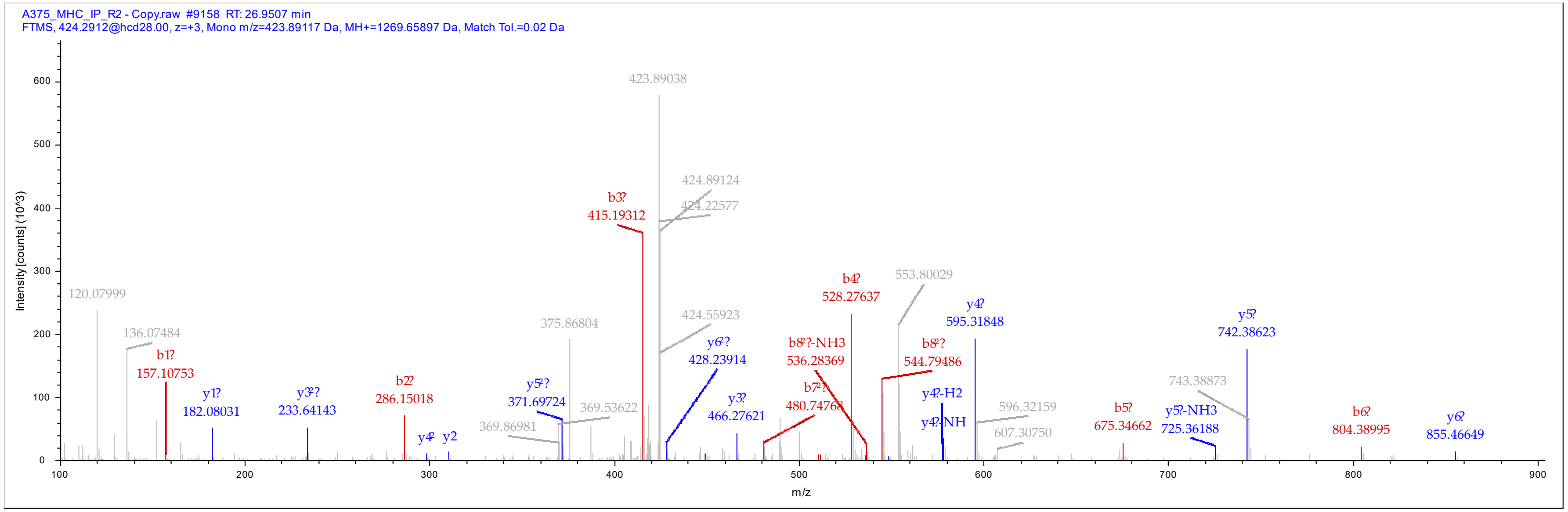

Spliced peptide - SAADPKVAF

Synthetic

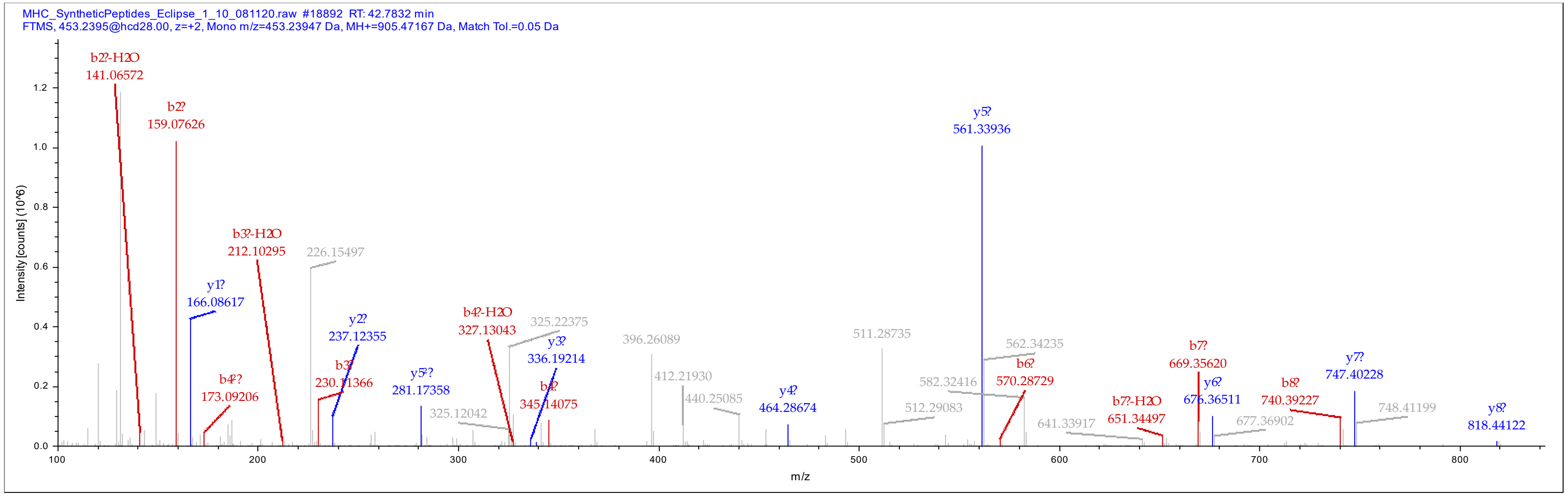

Experimental

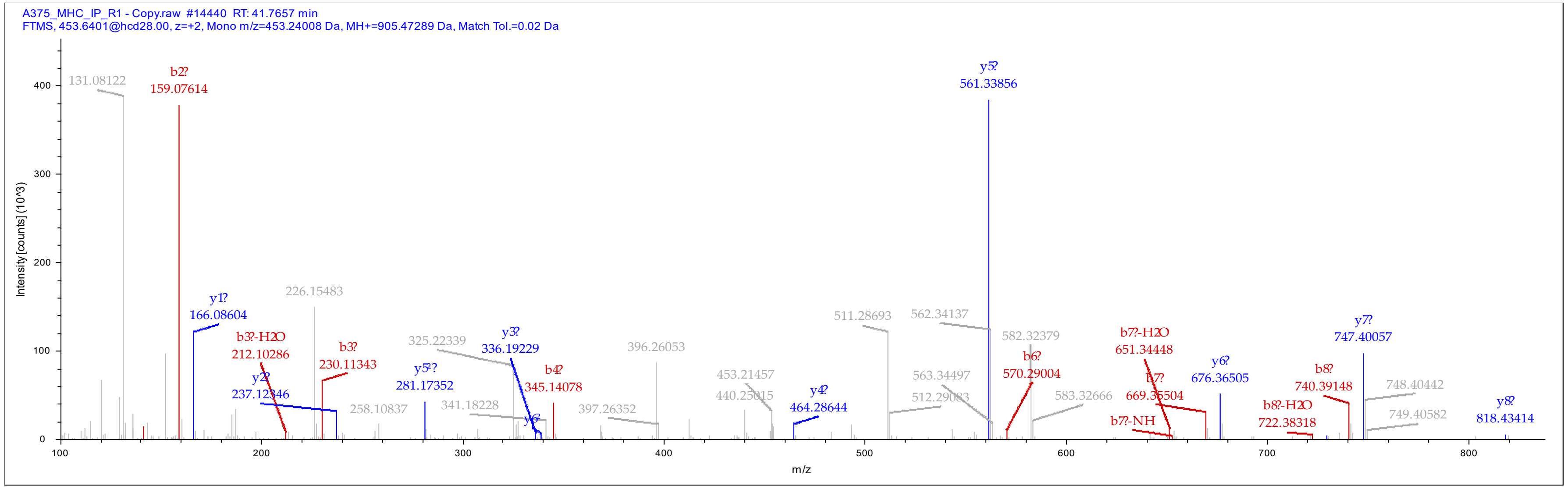

Spliced peptide - SAAERLLAF

Synthetic

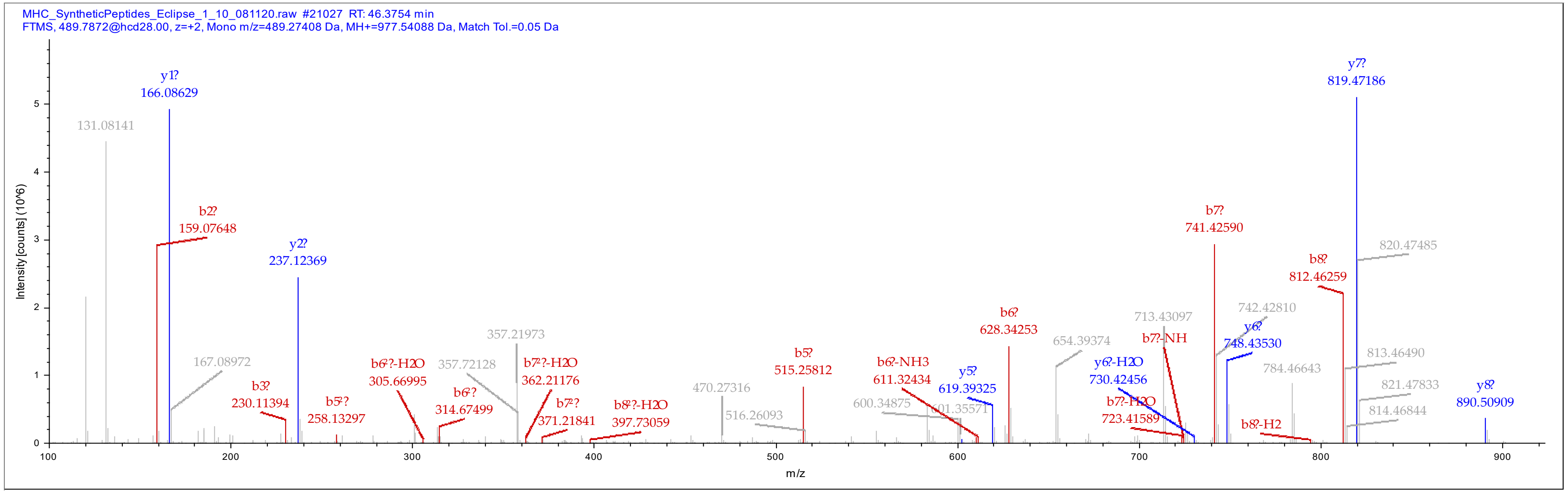

Experimental

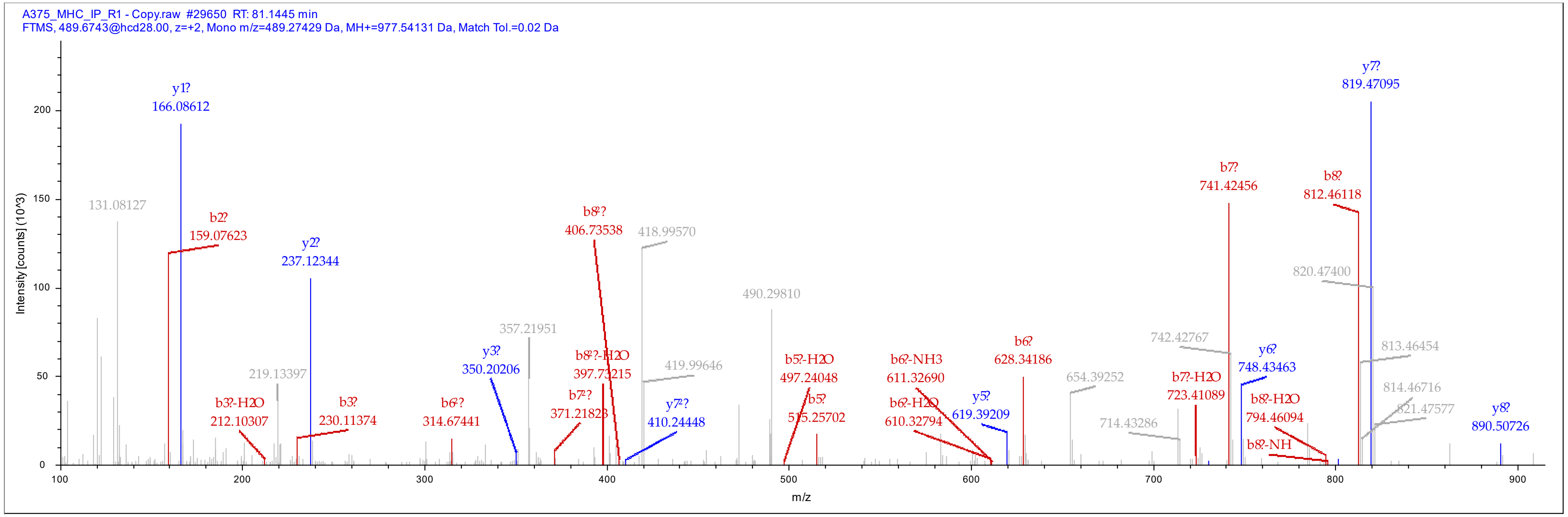

Spliced peptide - SAAWDRPPL

Synthetic

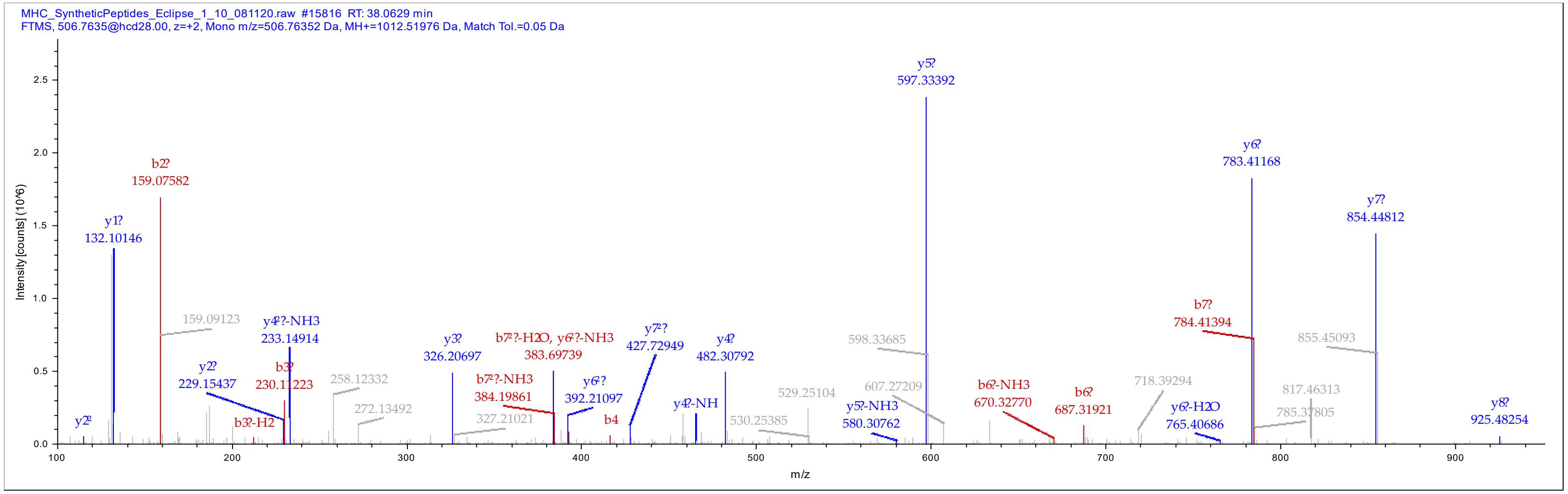

Experimental

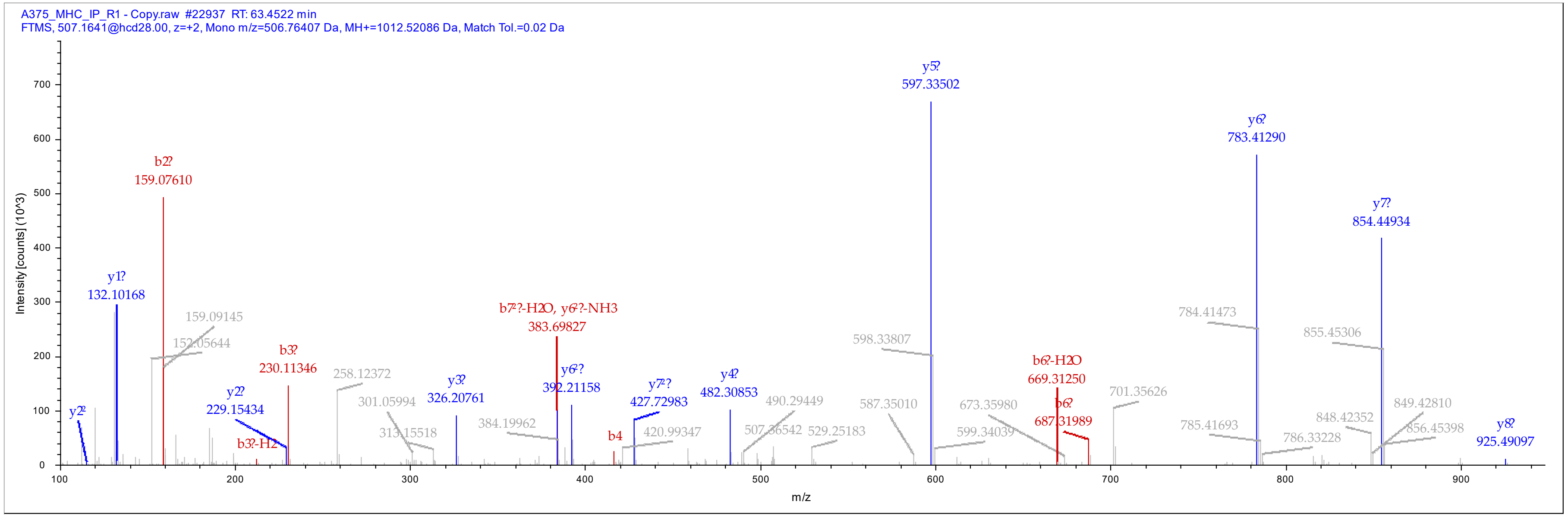

Spliced peptide - SELCGGANHSF

Synthetic

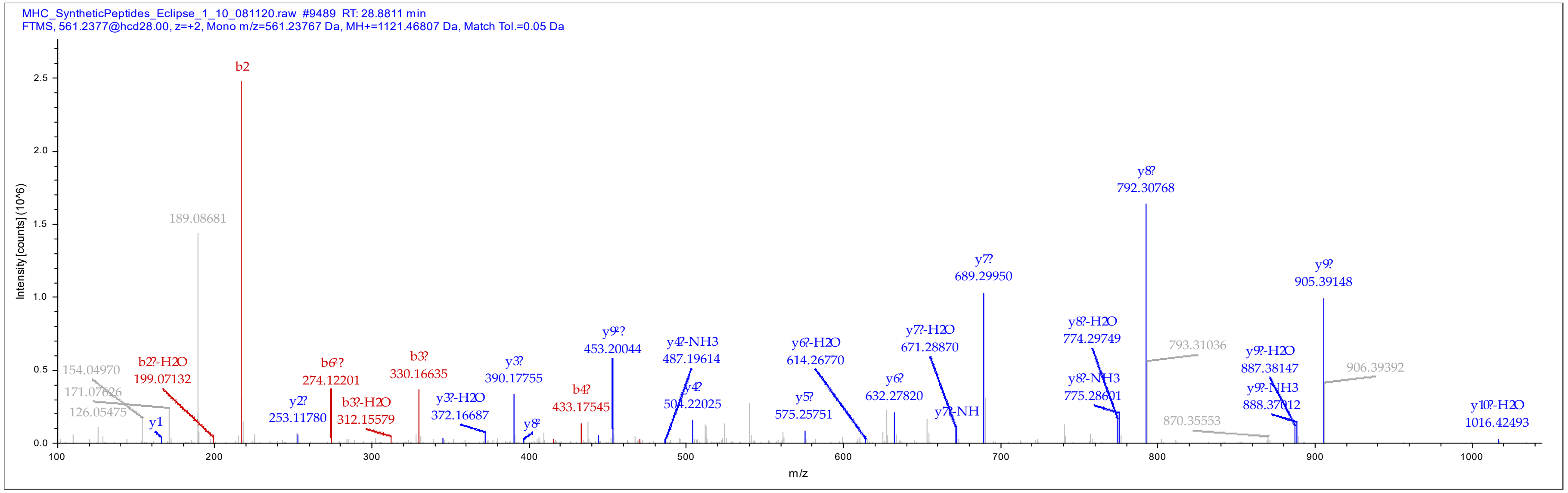

Experimental

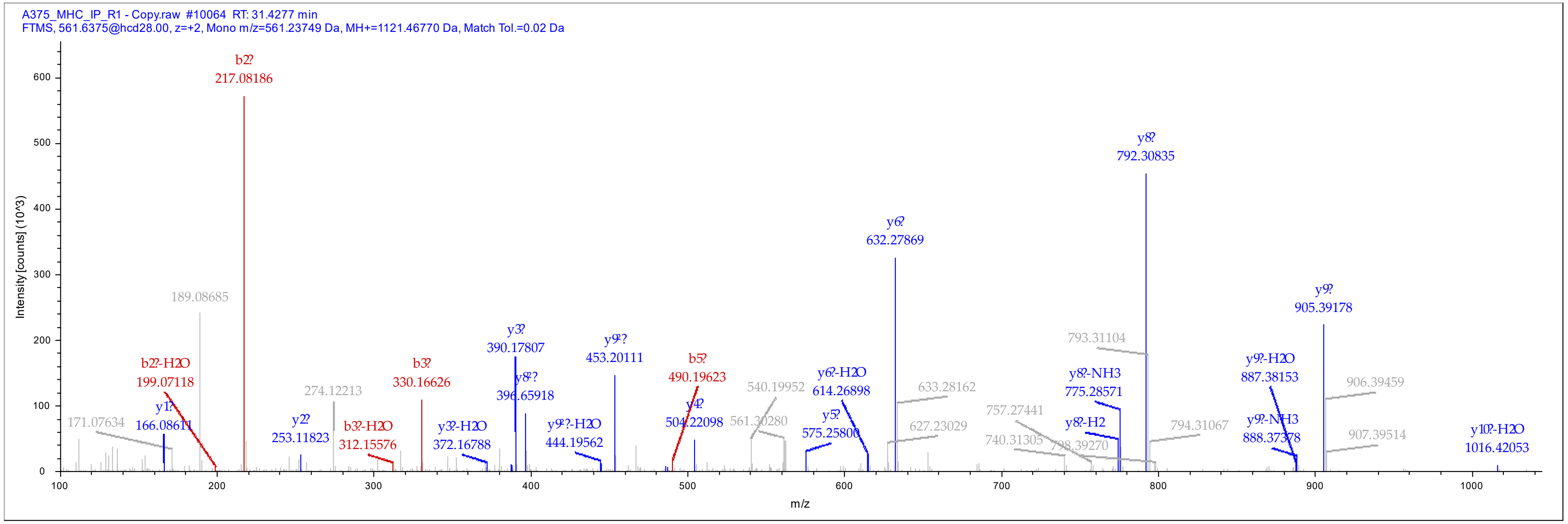

Spliced peptide - SESLLLTKKF

Synthetic

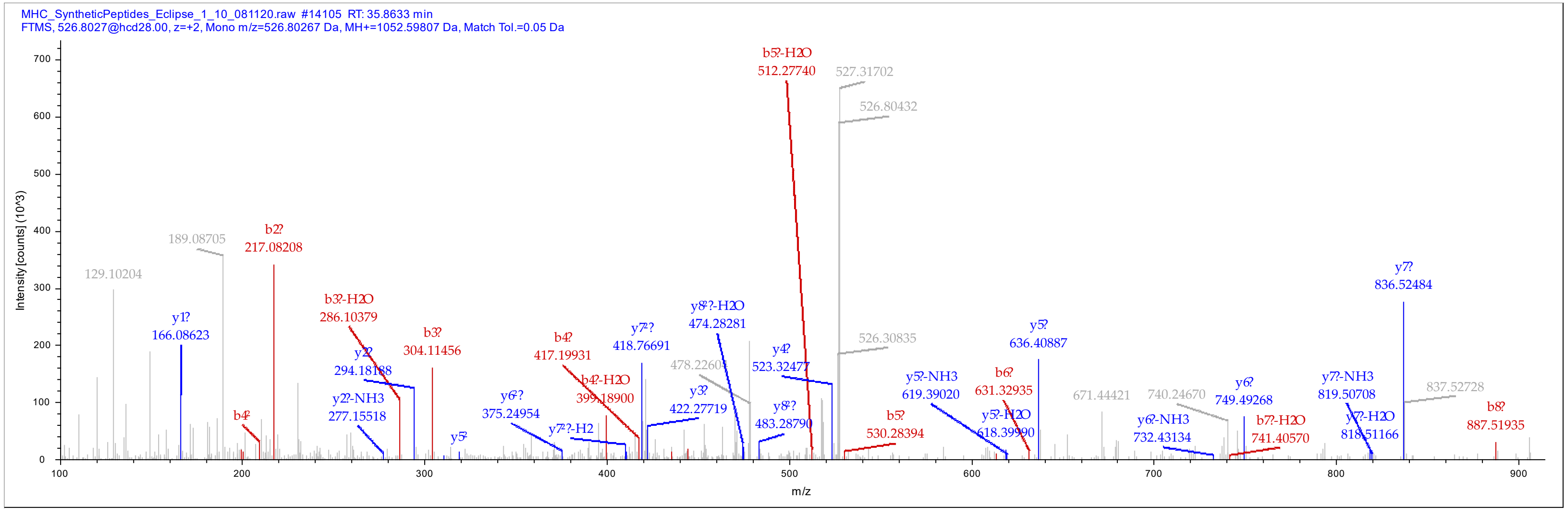

Experimental

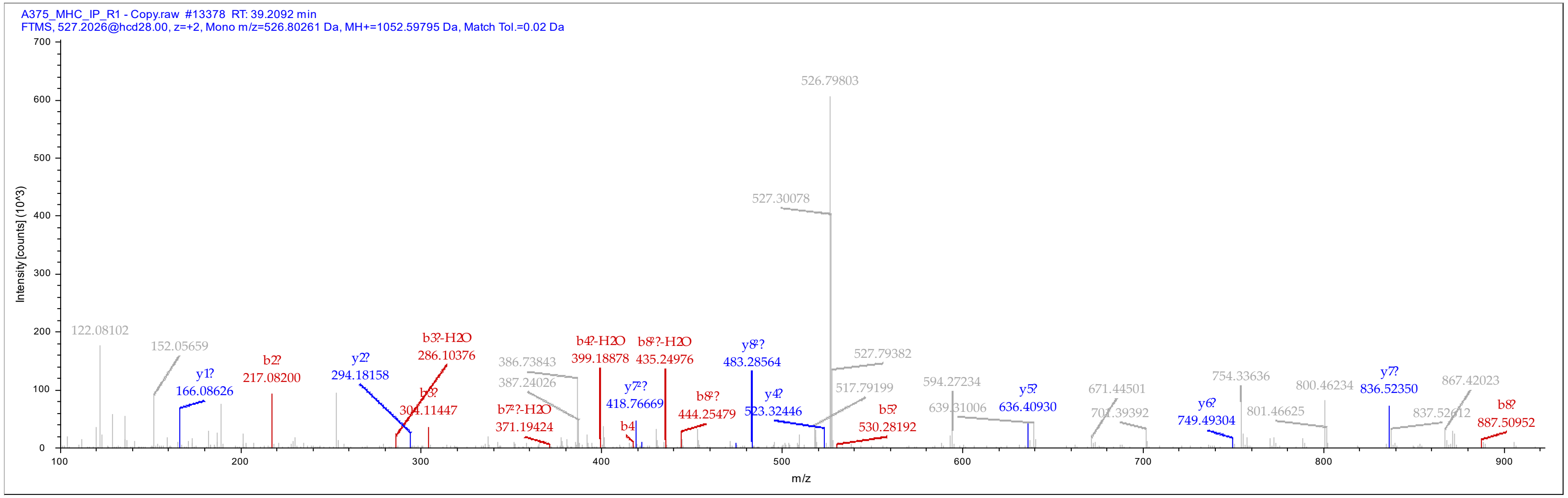

Spliced peptide - SPVQAAEVKVD

Synthetic

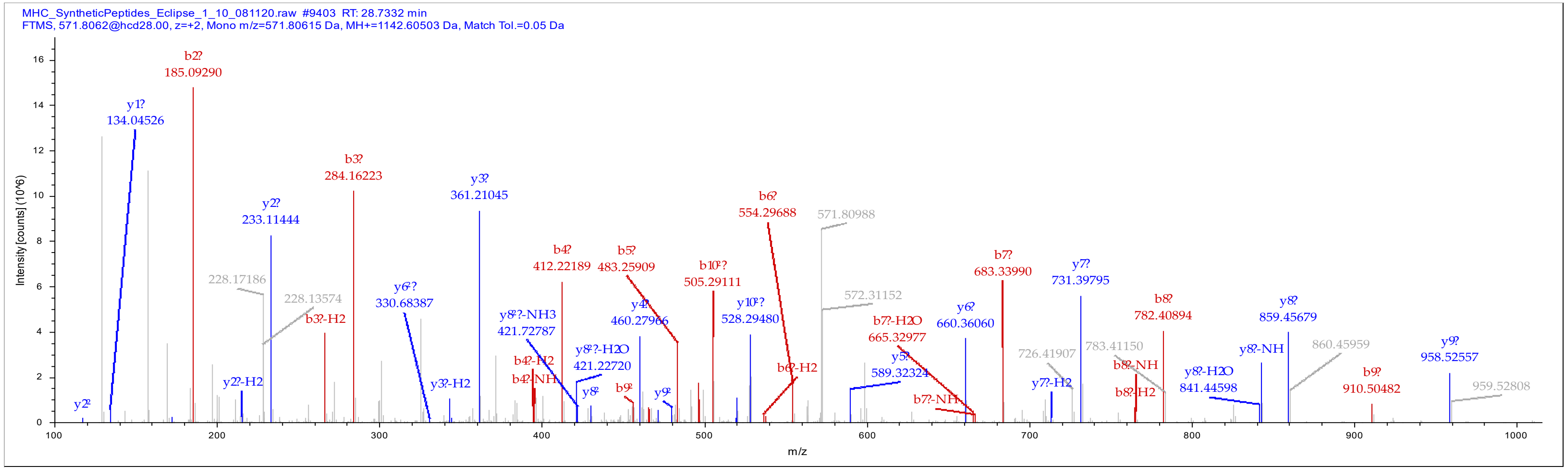

Experimental

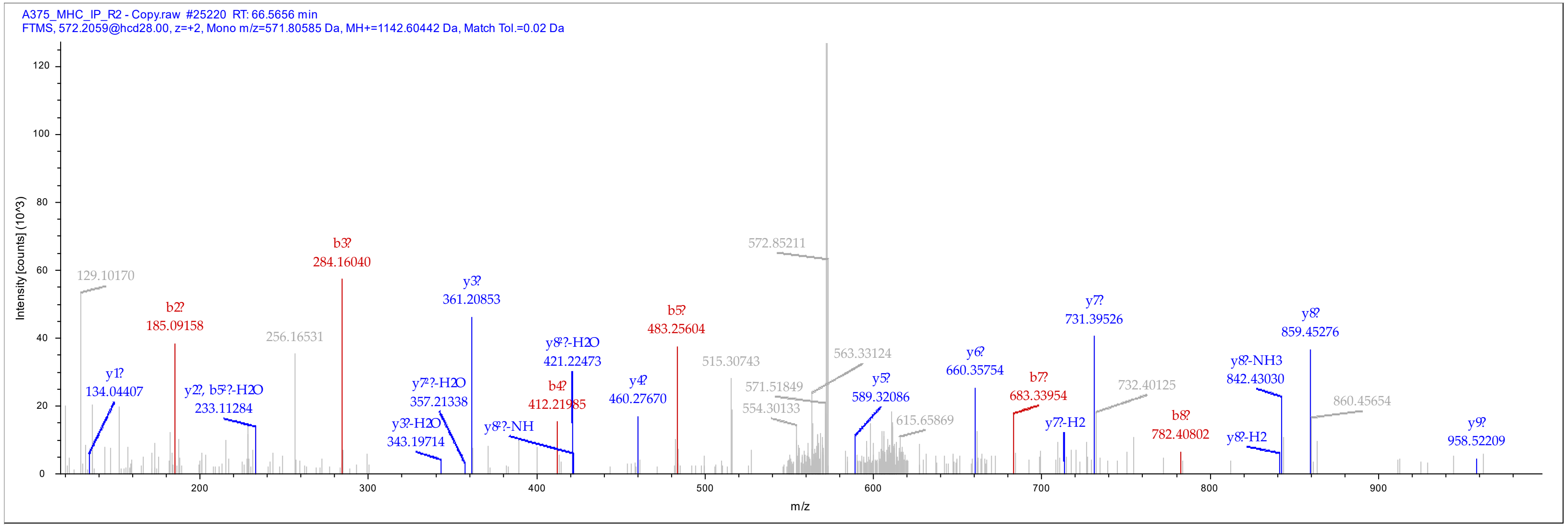

Spliced peptide - SYAVSVNHV

Synthetic

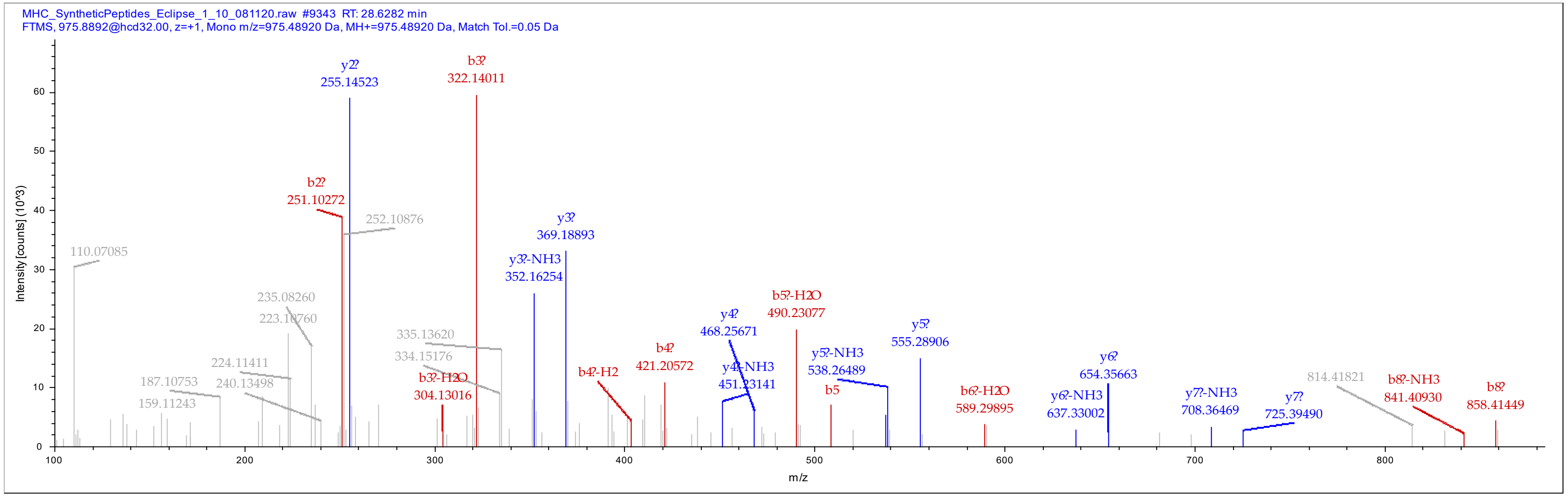

Experimental

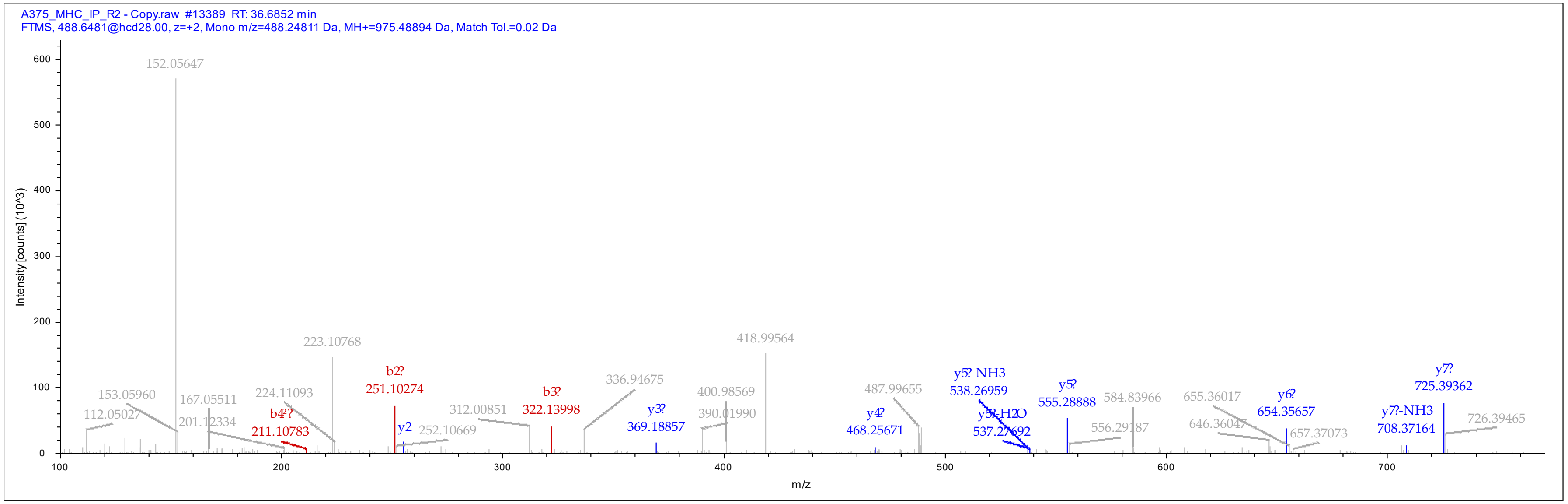

Spliced peptide - TELDVTKKY

Synthetic

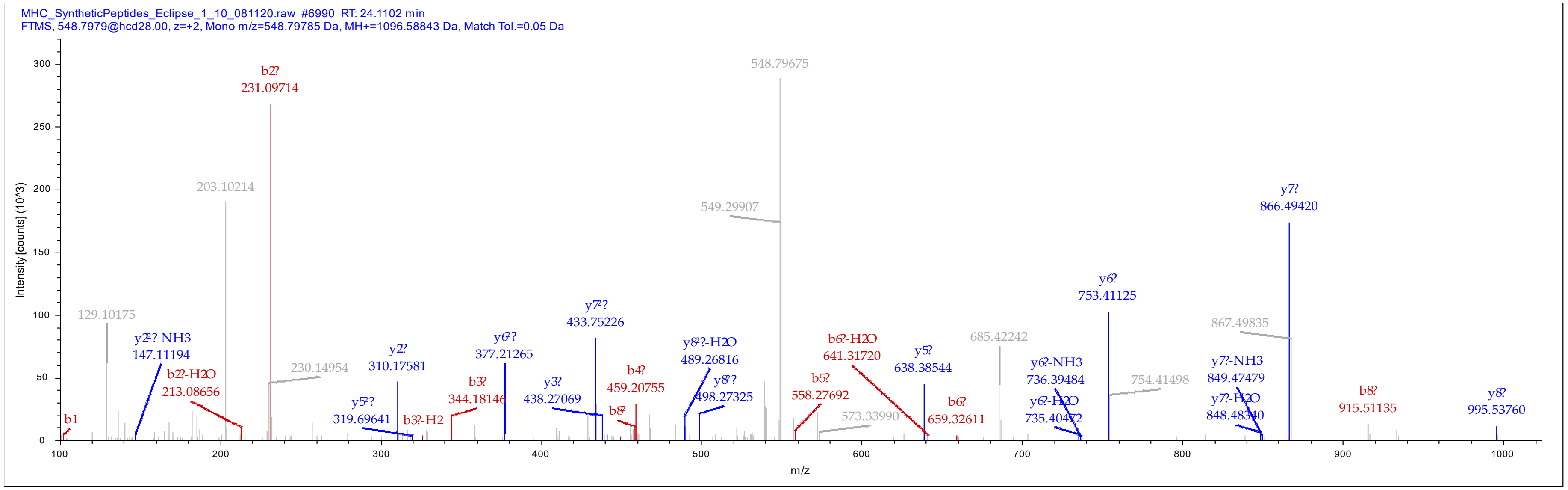

Experimental

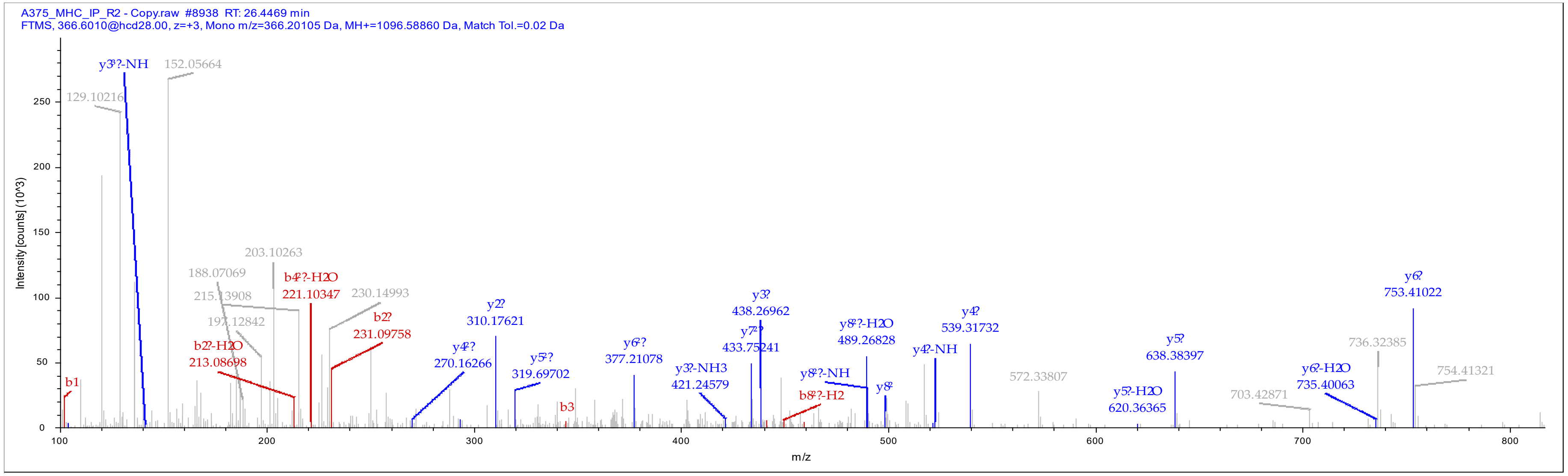

Spliced peptide - VGAGEGGLRNV

Synthetic

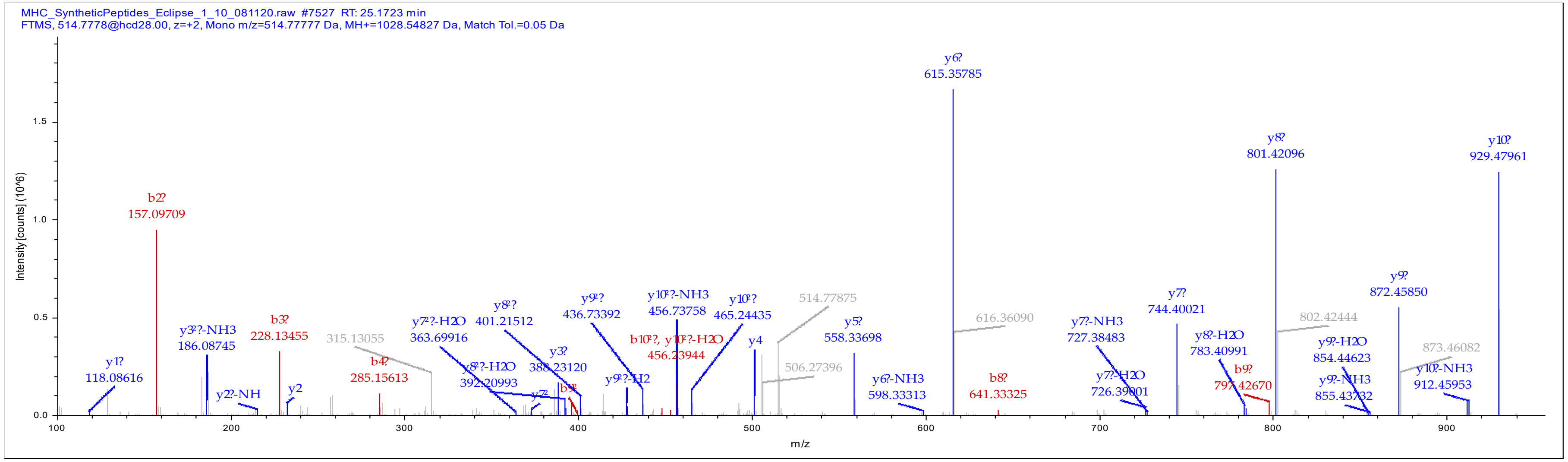

Experimental

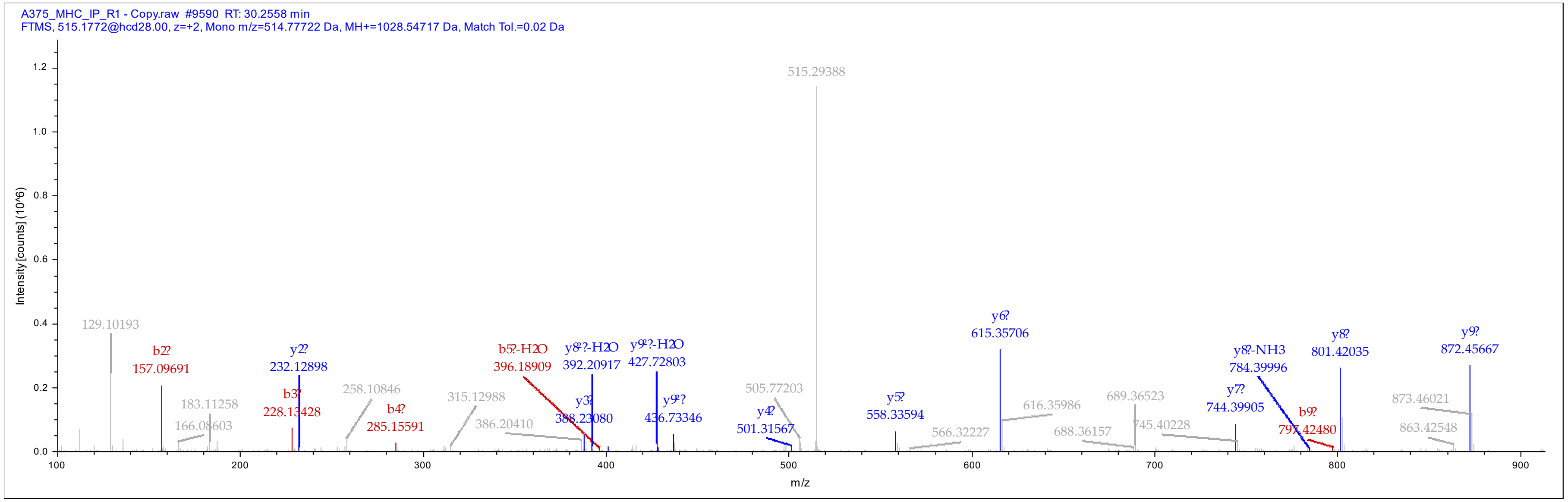

Spliced peptide - YTDDSPKYNY

Synthetic

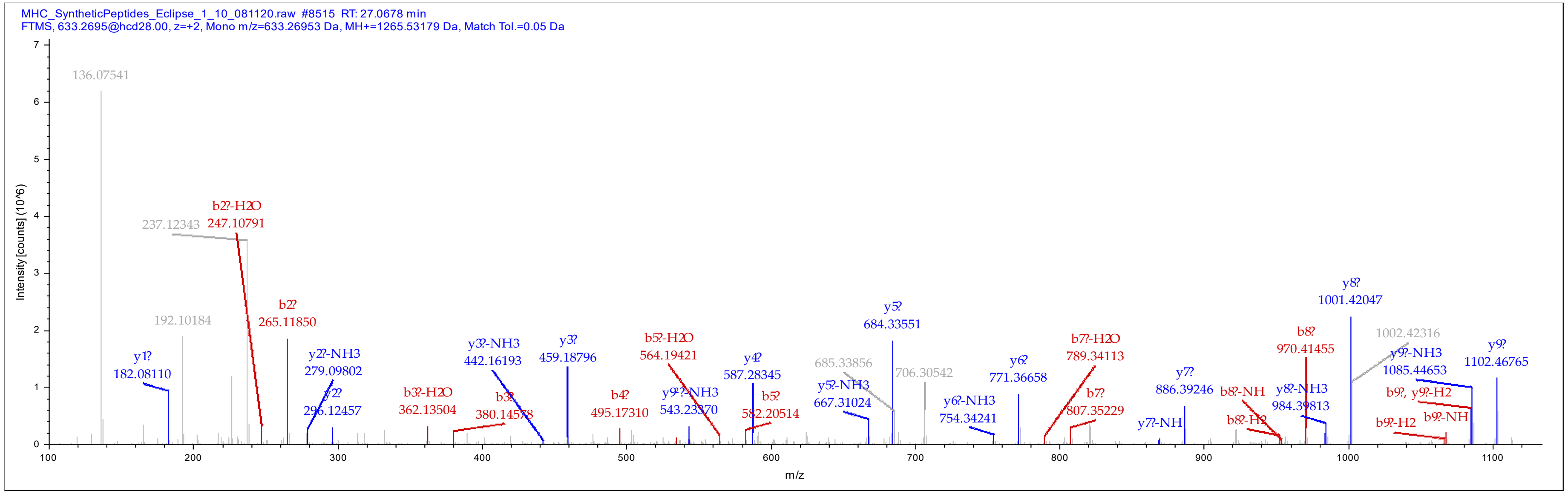

Experimental

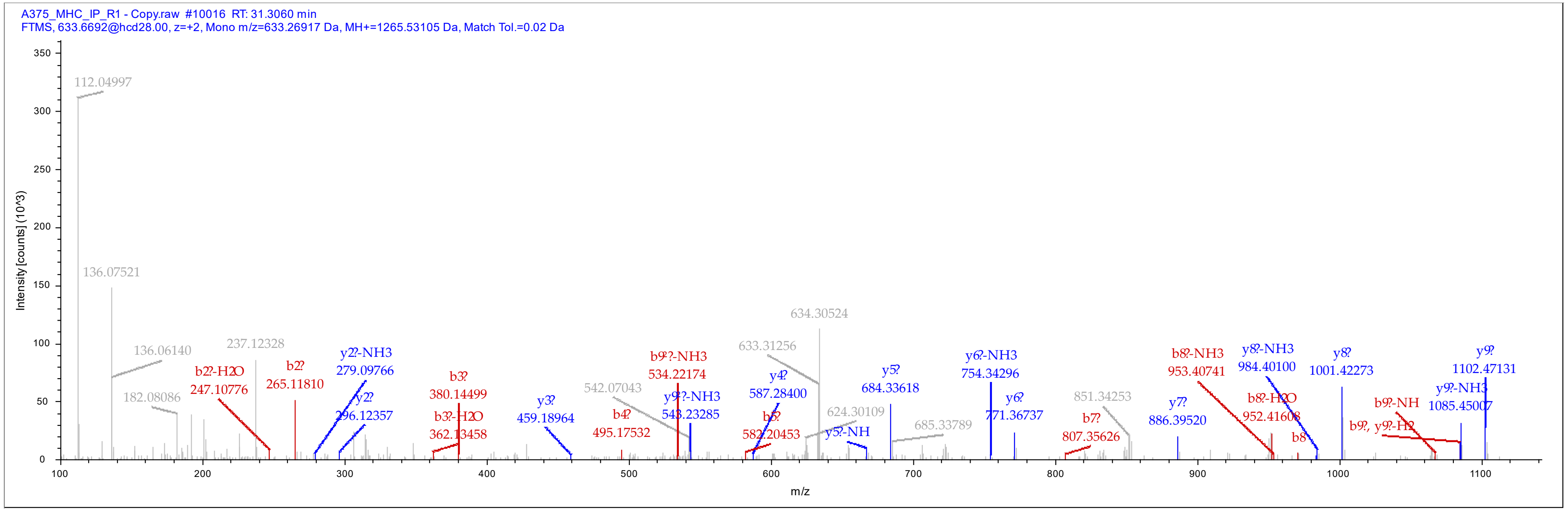

Spliced peptide - YTDFDGTHRY

Synthetic

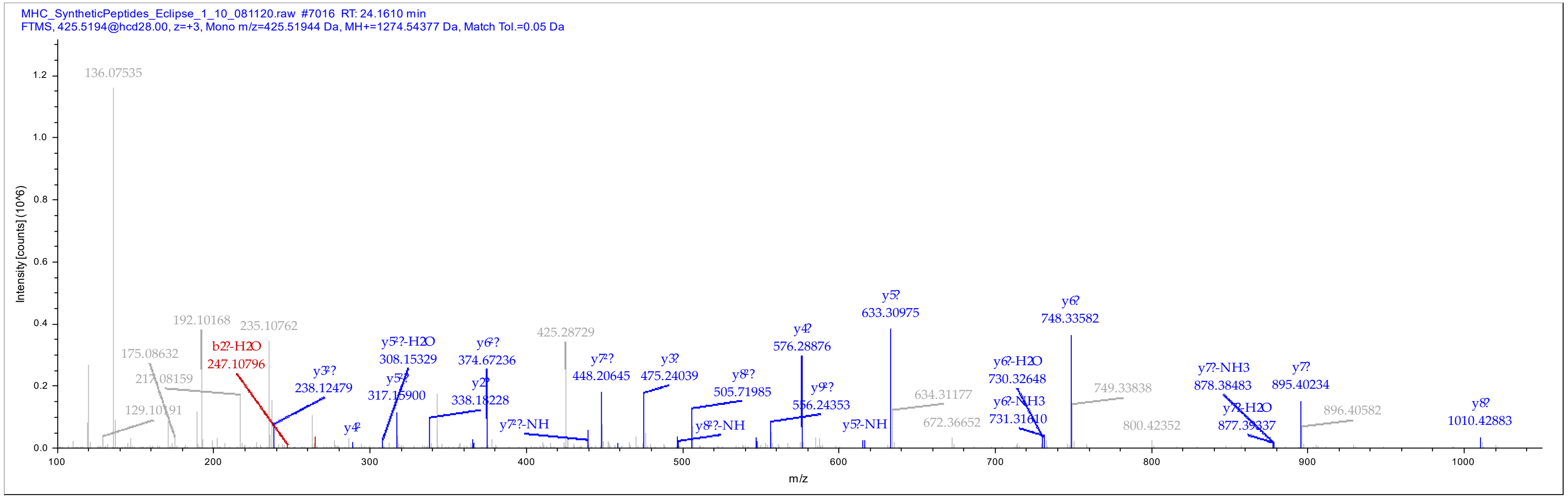

Experimental

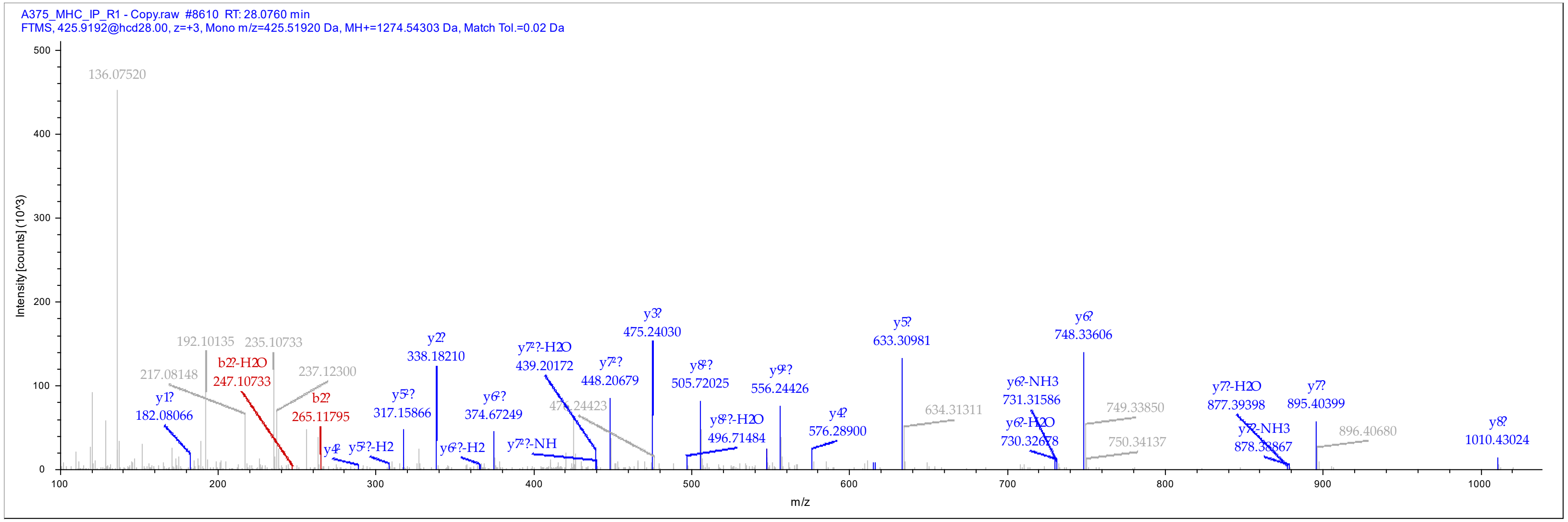

Spliced peptide - YTDNLVRVAM

Synthetic

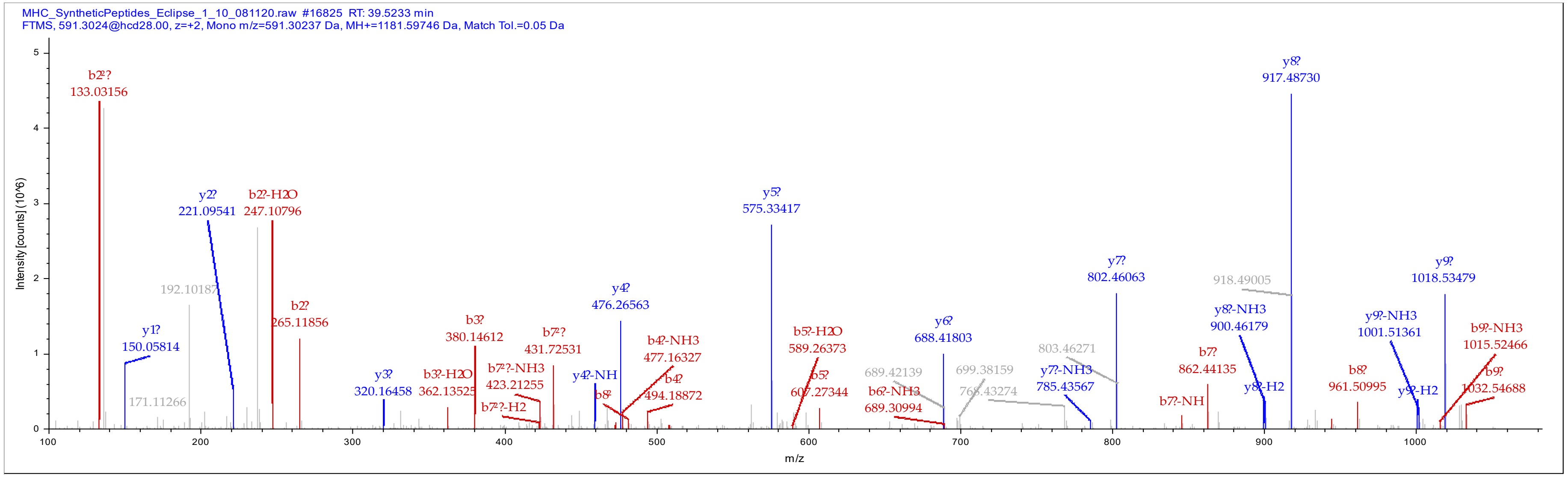

Experimental

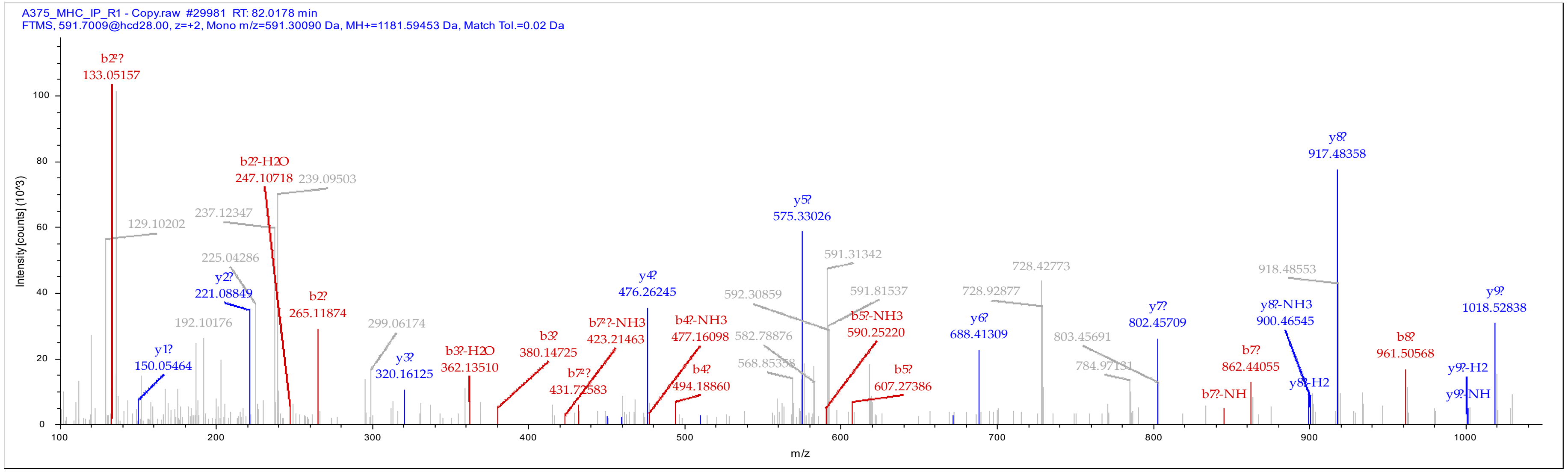

Spliced peptide - YTEFEDLKSGY

Synthetic

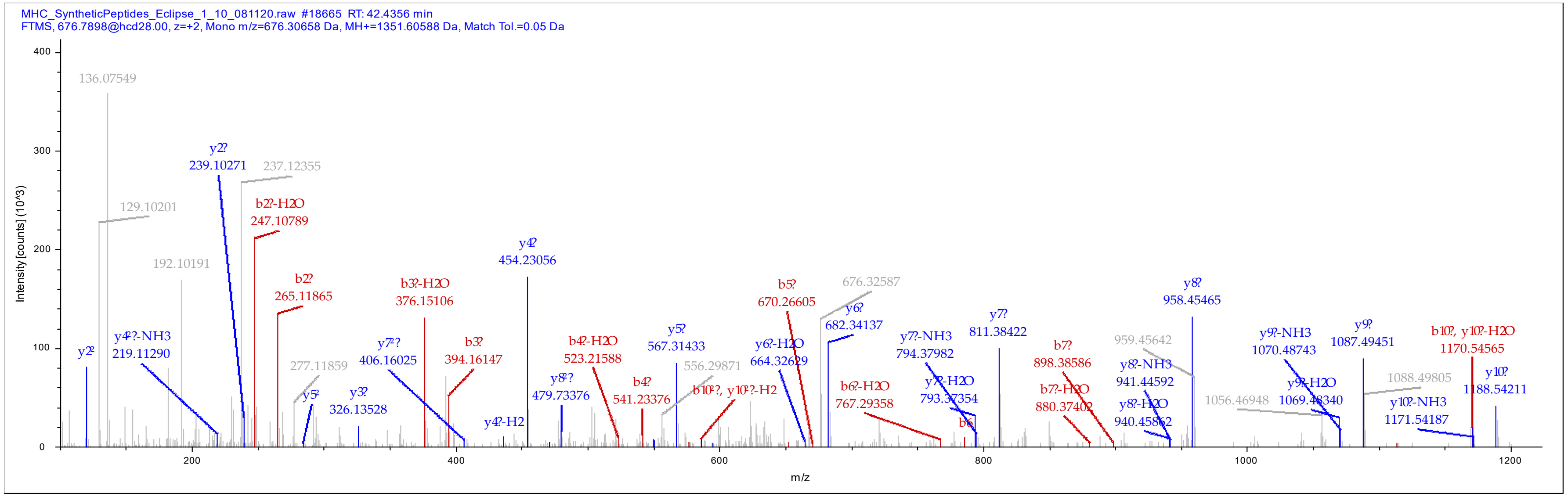

Experimental

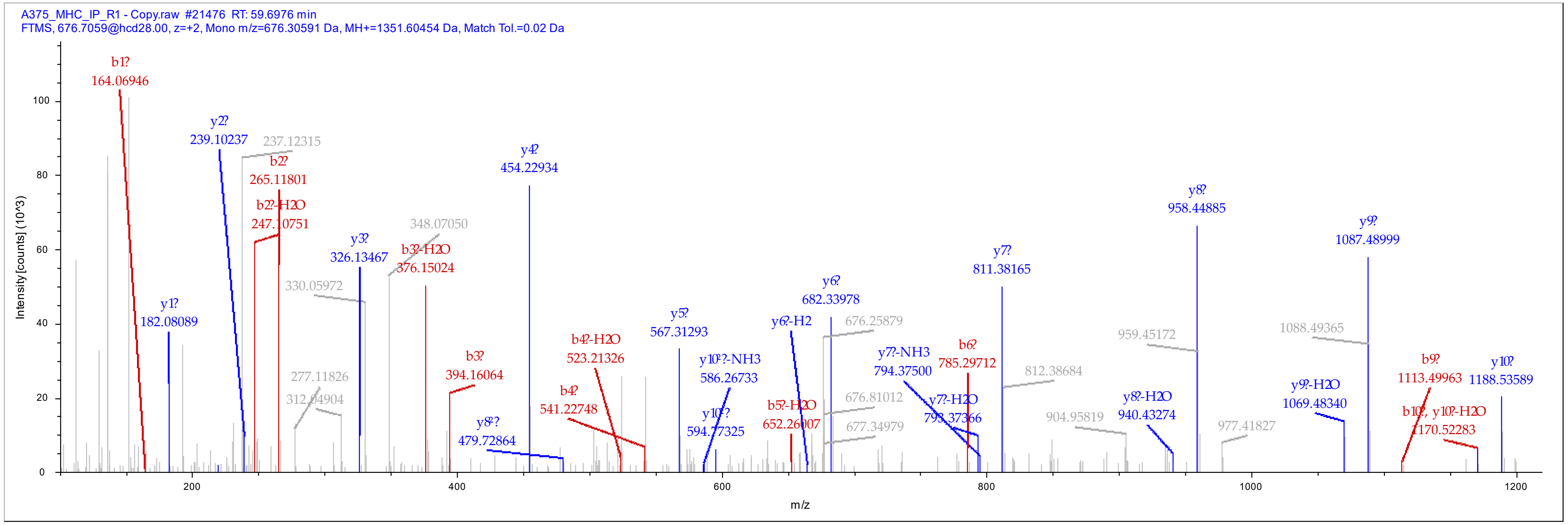

Spliced peptide - YVDCGGRNTTY

Synthetic

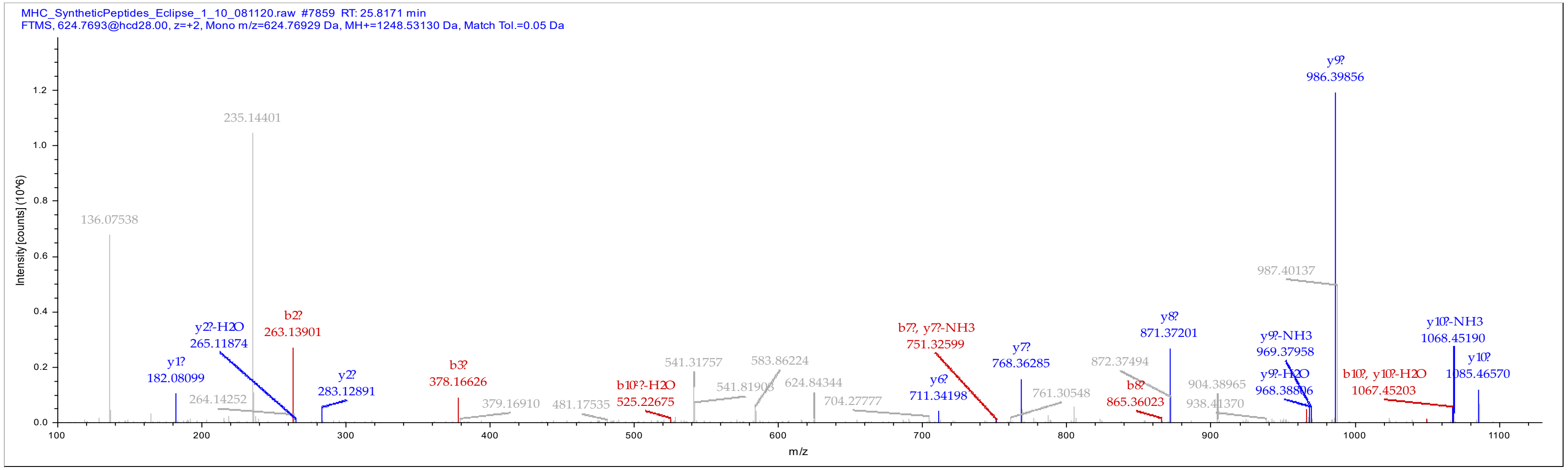

Experimental

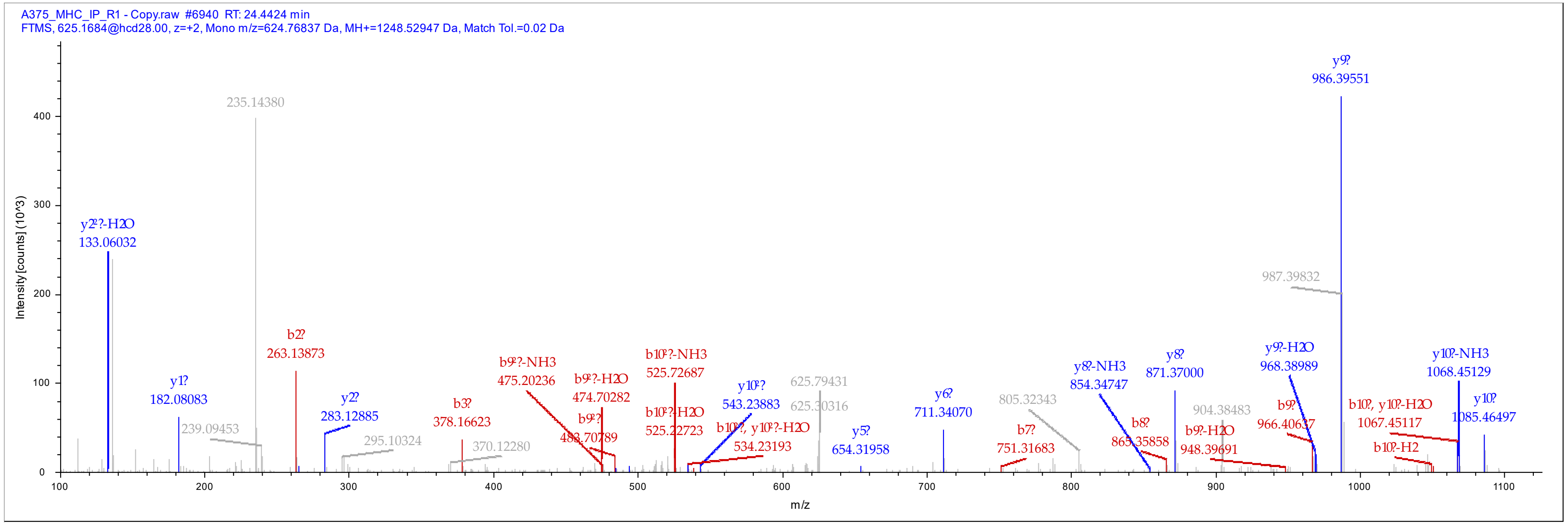

Supplement: Supplementary file 2 — Supplementary Figure 2: Overlay of the synthetic and experimental MS/MS spectrum of the peptides validated using the synthetic peptides (PDF 3068 kb) [file 42485_2021_66_MOESM2_ESM.pdf]
